# Distributional and species richness patterns of the stoneflies (Insecta, Plecoptera) in New York State

**DOI:** 10.3897/BDJ.13.e158952

**Published:** 2025-08-14

**Authors:** Luke William Myers, Boris C Kondratieff, Scott A Grubbs, Lindsey A Pett, R. Edward DeWalt, Timothy B Mihuc, Lily Veronica Hart

**Affiliations:** 1 Lake Champlain Research Institute, SUNY Plattsburgh, Plattsburgh, United States of America Lake Champlain Research Institute, SUNY Plattsburgh Plattsburgh United States of America; 2 Colorado State University, Fort Collins, United States of America Colorado State University Fort Collins United States of America; 3 Western Kentucky University, Bowling Green, United States of America Western Kentucky University Bowling Green United States of America; 4 Norwich University, Northfield, United States of America Norwich University Northfield United States of America; 5 University of Illinois, Champaign, United States of America University of Illinois Champaign United States of America

**Keywords:** Ecoregion, watershed, elevation, rare species, conservation

## Abstract

**Background:**

There is a 187-year history of stonefly (Insecta, Plecoptera) research in New York State. In total, 29 current valid species have a type locality in this state. Despite several new species' descriptions and numerous other papers discussing stoneflies in general from New York, a comprehensive treatment of the state's fauna is lacking. In this treatment we provide a comprehensive approach to assessing distribution and diversity patterns across multiple dimensions, focusing on adult flight periods, habitat associations, elevation gradients, United States Geological Survey Hierarchical Unit Code (HUC8) drainages, and United States Environmental Protection Agency (USEPA) Level IV Ecoregions.

**New information:**

This work is based on recent fieldwork, exhaustive searches of museums and research collections for specimens and accumulation of specimen data from peer-reviewed literature. Our analyses of 6,538 records from 1375 unique locations confirm the presence of 127 species in 42 genera across nine families, representing 58 of the 62 counties of the state. Nine new state records are presented with three known only from historical collections prior to 1970. Further analyses produced for all species include adult flight periods, elevational ranges, and distributional affinities across HUC8s and USEPA Level IV Ecoregions. This research will provide the basis for future conservation decisions in the state, identify gaps in our current knowledge, and elucidate needs for future research. A specimen data set has been associated with this document to aid in future assessments.

## Introduction

The rapidly changing climate and continued expansion of the human footprint is leading to dramatic declines in the entomofauna of the world (Sánchez-Bayo and Wyckhuys 2019, Wagner et al. 2021). Assessing the baseline of a fauna is difficult because of the lack of taxonomic expertise and the massive effort needed to accumulate pertinent historical and contemporary occurrence data. Often, studies fall short in these respects and depend entirely on contemporary data, frequently leading to a lack of historical context and a baseline that is biased toward recent sampling. This entails a shifted-baseline result (Soga and Gaston 2018).

**Figure 1.**
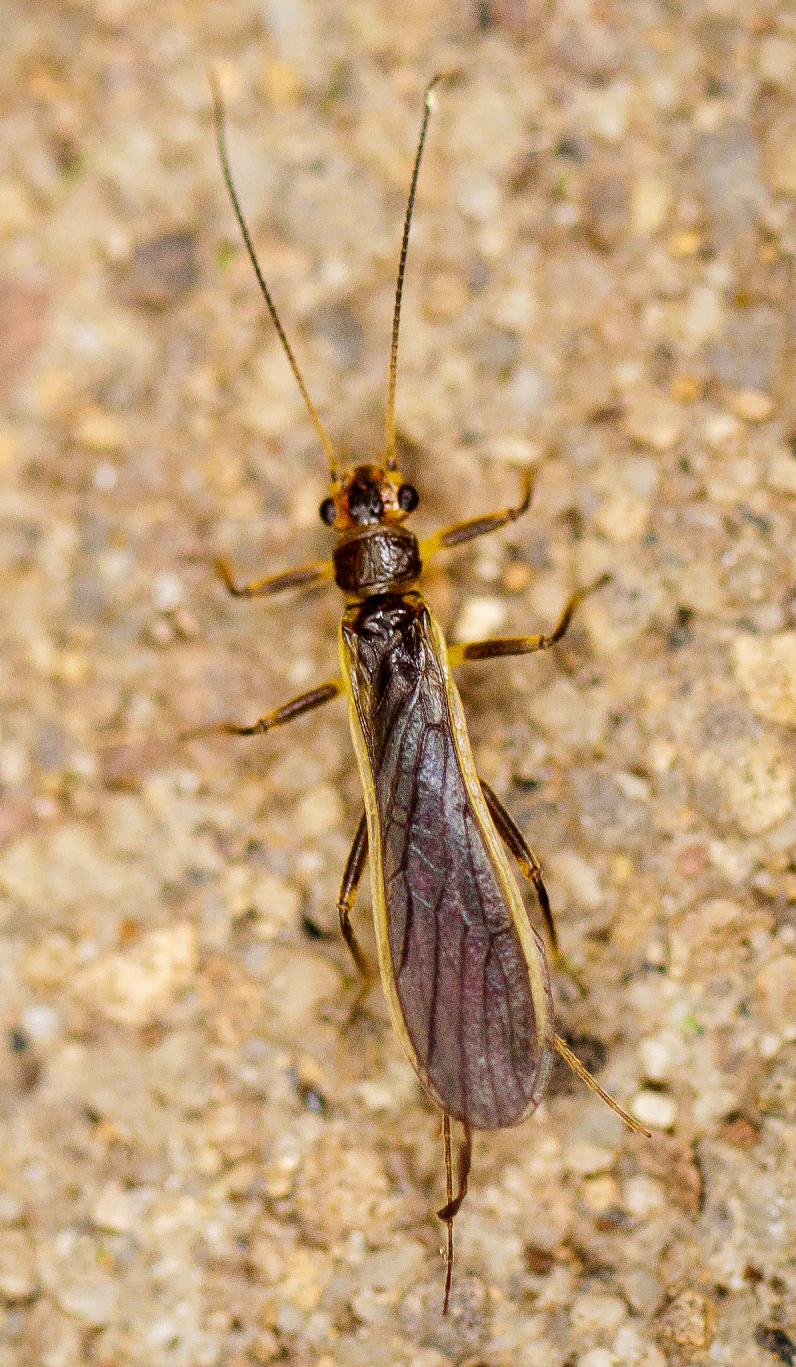
Adult male of *Perlestamihucorum* from Hannacroix Creek in Greene County New York

We present a monograph of the Plecoptera (stoneflies) (Fig. 1) of New York State that is predicated on balancing the historical and contemporary data sources through re-examination of historical museum specimens, contemporary sampling of the entire state, and accumulation of trusted literature records into a single occurrence data set. This data set is provided to aid New York State conservation organizations in their efforts to assess the conservation status of this environmentally sensitive order of insects. In addition, we recount the history of stonefly research conducted in New York, discuss the known sensitivity of stoneflies to changes in climate and water quality, and summarize the pertinent physical attributes of New York state as they pertain to stonefly distribution and macroecology. We provide an updated checklist and discussion of each species, conduct analyses of completeness of sampling, assess stonefly diversity relationships to drainages and ecological classifications, and provide detailed distributional maps for all species. It is unfortunate that this paper is being published posthumously for one of its authors, Dr. Boris C. Kondratieff (DeWalt 2023), who contributed greatly to this work.

The state of New York has served a pivotal role during the early years of aquatic insect taxonomy in North America, including Plecoptera or stoneflies. The New York State Museum (Albany) and Cornell University (Ithaca) were some of the earliest institutions in the country supporting research in aquatic insect taxonomy and ecology in North America. The first New York stoneflies were described and documented before 1840 (Newman 1838, Newman 1839). Soon after, other early and important descriptions of stonefly taxa from New York were published by Fitch (1847), Banks (1897), Banks (1911), Smith (1917), Wu (1923), Claassen (1923), Claassen (1924), Needham and Claassen (1925), Claassen (1931), Claassen (1937). Needham and Claassen (1925) published a summary of the number of species that included 56 from New York State. In a curious reversal, Needham and Claassen (1926) published the first list of insects known from New York State, that included 40 species of stoneflies, 38 of which are currently valid (DeWalt et al. 2024). Of the 770 known North American stonefly species through 2022 (DeWalt et al. 2024), 29 (= 3.7%) have type localities in New York State (Table 1).

Historically, the vicinity of Cornell University was a heavily collected area. Many of these early collections from and near Ithaca and other entomological field stations across the state were conducted by the well-known aquatic entomologists J. G. Needham and P. W. Claassen. Other sporadic collections and historical records of stoneflies are available from near the Saranac Inn in Adirondack Park and some from what is now included in the New York metropolitan area and Long Island. Other early collections of stoneflies were by E. Doubleday, W. T. Davis, and C. P. Alexander. Later, collecting efforts by H. Dietrich, T. H. Frison, H. H. Ross, L. L. Pechuman, T. L. McCabe, and R. W. Baumann yielded numerous additional descriptions and records.

As part of a broader study focusing on mayflies (Ephemeroptera), stoneflies, and caddisflies (Trichoptera) of Adirondack Park, Myers et al. (2011) reported 100 species of stoneflies including 23 documented from New York State for the first time. The Adirondack Park is the largest protected natural area in the lower 48 USA states at 2,428,114 ha (New York State 2023) and encompasses approximately one-third of the total land area of New York State. However, complete distributional data was only presented for species of general conservation status and others reported from the state for the first time. In the same year, Kondratieff and Myers (2011) described *Perlestamihucorum* from the Hudson River Valley. More recently, Szczytko and Kondratieff (2015) described two new species of *Isoperla* Banks, 1906 from New York plus providing several new state records.

Stoneflies are sensitive to environmental change (Baumann 1979, Rosenberg et al. 1986, Rosenberg and Resh 1993, Bojková et al. 2012, Giersch et al. 2017, Sánchez-Bayo and Wyckhuys 2019) and anthropogenic disturbances may have led to species-level extirpations in New York State, as has been reported previously for USA Midwestern states of Illinois (DeWalt et al. 2005), Indiana (DeWalt and Grubbs 2011, Newman et al. 2021), Michigan (Grubbs et al. (2012), and Ohio (DeWalt et al. 2012, Grubbs et al. 2013). Illinois has been the USA state with the greatest loss, with two extinctions and 20 extirpations. Wilcove and Master (2005) indicated that at least 21% of all North American stoneflies are imperiled. According to the Nature Conservancy and the Association for Biodiversity Information, stoneflies are ranked as one of the most imperiled groups of freshwater organisms in the United States. Over 43% of the current North American stonefly fauna are classified as “Vulnerable”, “Imperiled”, or “Presumed or Possibly Extinct” (Stein et al. 2000). This is problematic for stoneflies because recovery from disturbances is often slow because many taxa have low vagility (Peterson et al. 2004) and dispersal is often restricted to suitable habitats within a catchment (Bunn and Hughes 1997, Briers et al. 2002, Peterson et al. 2004).

### Physical setting

**Figure 2.**
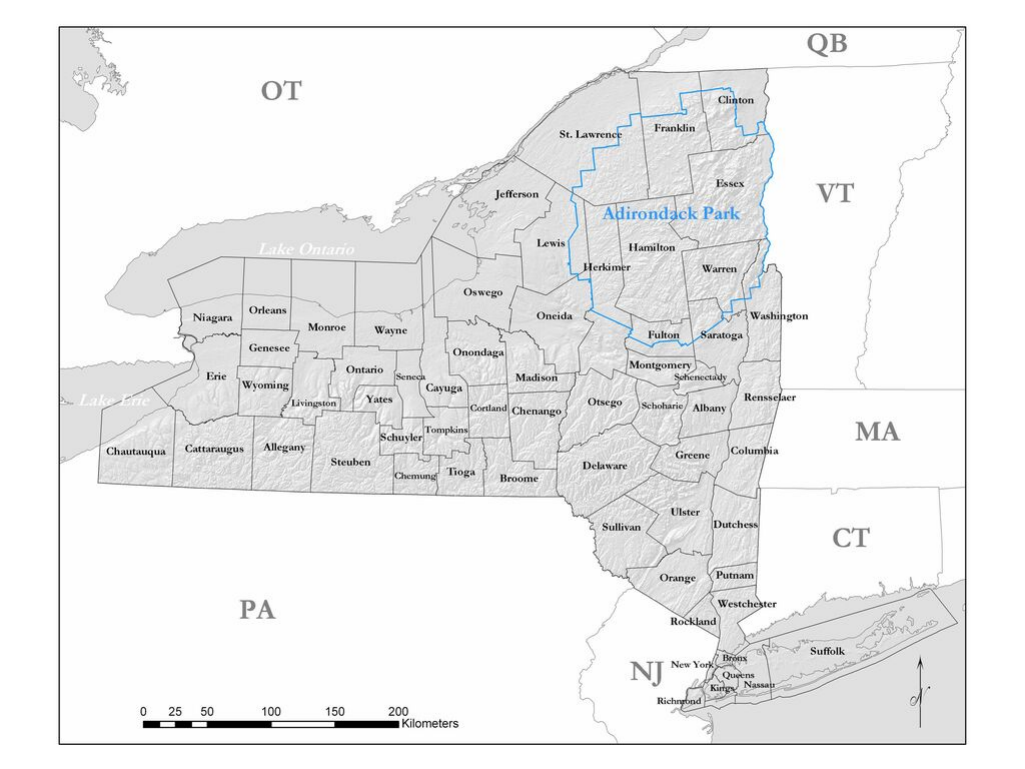
Map showing 62 counties in New York State and the location of Adirondack Park in the light blue outline. Canada: OT = Ontario, QB = Quebec; USA: CT = Connecticut, MA = Massachusetts, NJ = New Jersey, PA = Pennsylvania, VT = Vermont.

**Figure 3.**
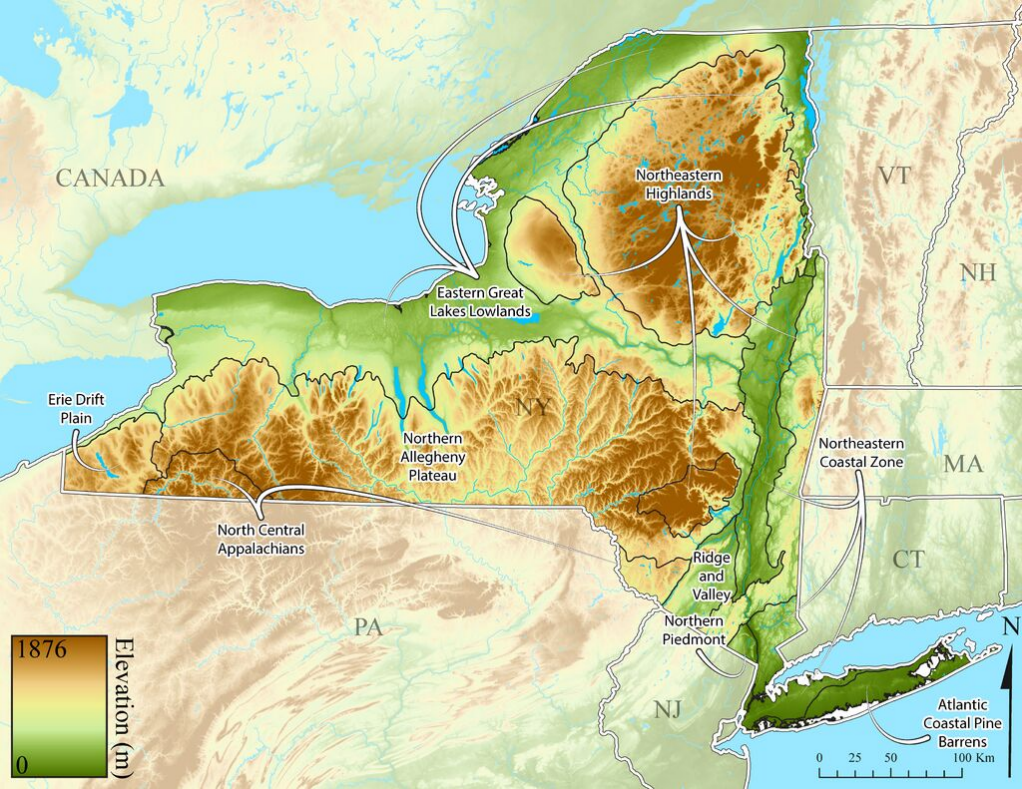
USEPA Level III ecoregions in New York State with elevation overlay for the region.

**Figure 4.**
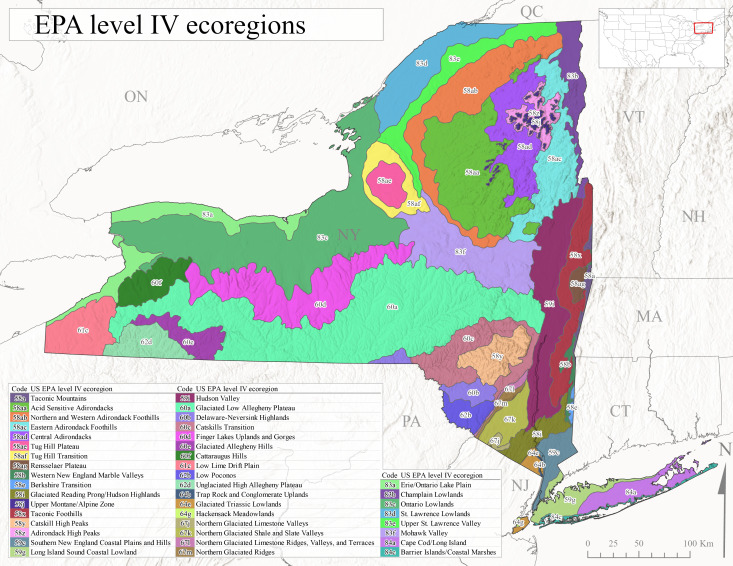
Map of USEPA Level IV Ecoregions in New York State.

New York State is comprised of 62 counties (Fig. 2) encompassing approximately 87,000 km^2^ of terrain (Thompson 1980), nine USEPA Level III Ecoregions (Fig. 3), and 42 Level IV Ecoregions (Fig. 4). Elevation ranges from sea level along several km of tidal flat coastline to 1,628 m at the summit of Mount Marcy in Adirondack Park (United States Geological Survey 2005). The Adirondacks in the north and the Catskills in the south are the highest and most extensive mountain ranges in the state (Fig. 4). Between these two mountain ranges, and on the northern and eastern borders of the state, lies a network of lowlands, including the Great Lakes Plain, the Hudson, Mohawk, Lake Champlain, and St. Lawrence River valleys, and the coastal plain areas of New York City and Long Island (Fig. 4). Less prominent upland regions of the state include the Appalachian Plateau, the Finger Lakes Highlands, the Taconic Highlands, the Tug Hill Plateau on the western edge of the Adirondacks, and a series of smaller mountain ranges flanking the Hudson River Valley in the southern and central portions of the state (Fig. 4).

New York State's varied topography, elevation, and proximity to several large bodies of water results in variable precipitation and climate patterns (Carter 1980, New York State Climate Office 2008). During the summer months the state is dominated by warm, moist air originating mostly over the Atlantic Ocean. A mixture of warm moist air from the Atlantic and cool dry air from the north and west are common in autumn and spring. During the summer and autumn, temperatures remain constant across the state. In the winter and early spring, cold air masses originating from the interior of the continent bring the greatest spatial variations in temperature across the state (Carter 1980). During winter, coastal areas experience temperatures that remain around freezing point with very little snow accumulation. In contrast, areas in the northeastern portion of the state average > 4.4 m of snow each year (New York State Climate Office 2008). Average annual mean temperatures range from 4.5 ºC in the Adirondacks to 12.8 ºC in New York City (New York State Climate Office 2008).

Northern hardwood forests cover much of the forested land area of the state (Richardson et al. 2006). This forest type occurs primarily at low to mid-elevations and on well-drained soils (De Laubenfels 1980). Characteristic species include *Fagusgrandifolia* Ehrh. (American beech), *Acerrubrum* L. (red maple) *A.saccharum* Marsh (sugar maple), *A.pennsylvanicum* L. (striped maple), *Fraxinusamericana* L. (white ash), *Tsugacanadensis* (L.) (eastern hemlock), *Thujaoccidentalis* L. (northern white cedar), and *Pinusstrobus* (white pine). At lower elevations of the St. Lawrence, Lake Champlain and Lake Ontario valleys, *Quercusrubra* L. (northern red oak), *Q.alba* L. (white oak), and *Q.macrocarpa* Michx. (burr oak) mix with northern hardwood species (De Laubenfels 1980, Kudish 1992). Pitch pine barrens and various southern oak species replace the northern hardwood forest in the southern tier of the state, from the lower Hudson River south to the New York metropolitan area and Long Island. At higher elevations, primarily in the Tug Hill Plateau and the Adirondack and Catskill mountains, characteristic northern hardwood species become less abundant and give way to the more dominant boreal forest species, namely *Picearubens* Sarg. (red spruce), *Abiesbalsamea* (L.) (balsam fir), *Betulapapyrifera* Marsh (white birch), *B.alleghaniensis* Britton (yellow birch), and *Acerspicatum* Lam. (mountain maple) (De Laubenfels 1980, Kudish 1992). Today, some of the most extensive tracts of this forest type in northeastern USA occur in the Adirondack and Catskill state parks. These two parks are a unique mixture of private and public lands and consititute >12,000 km^2^ of state-owned Forest Preserve that are protected by the “Forever Wild” Clause (Article XIV) of the State Constitution.

Anthropogenic disturbances have undoubtedly affected the current distributional patterns of forest types (Swaney et al. 2006). From the establishment of the first European colonist settlements in New York at the start of the 17th century until the early 20th century, much of the forest landscape of the state was initially cleared by timber harvest, leading to agricultural industrial development and urbanization, all impacting aquatic habitats (Fox 1902, Harrison 2002, Swaney et al. 2006). Substantial industrial and urban development has occurred throughout much of the state since the turn of the 19th century. Extensive forest regrowth has occurred following the abandonment of agricultural lands in the less productive and rocky soils of upland areas of the state (Klepeis et al. 2013). Agriculture is currently a dominant land use for lowland areas of the state, while large tracts of forest remain in the Catskills, Alleghany Plateau, and Adirondacks.

**Figure 5.**
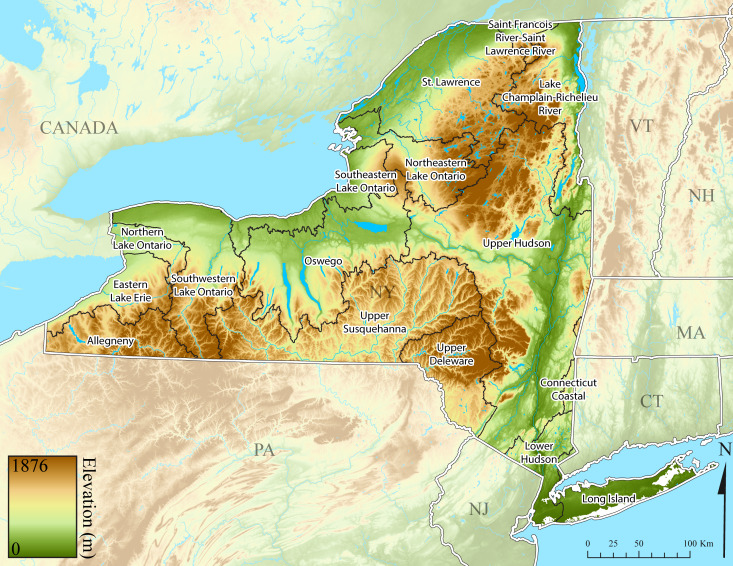
Map of 12 USGS HUC 6 watersheds in New York State with elevation overlay for the region. USA: CT = Connecticut, MA = Massachusetts, NH = New Hampshire, NJ = New Jersey, PA = Pennsylvania, VT = Vermont.

**Figure 6.**
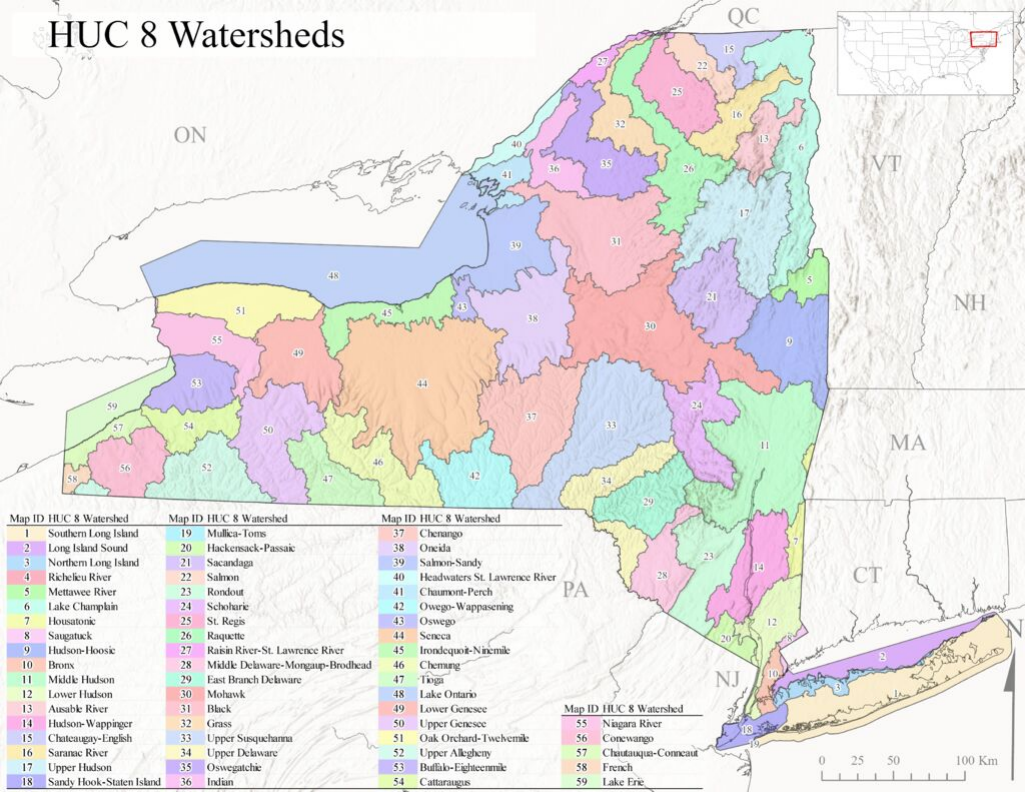
Map of 52 HUC 8 subwatersheds in New York State.

The state’s approximately 113,000 km of streams provides a broad diversity of lotic habitats ranging from high elevation seeps and springs to the Hudson River, one of the largest river systems in the eastern United States (Thompson 1980, Zembrzuski and Gannon 1986). Lentic habitats are also plentiful in the state, with >24,000 lakes and ponds and >40 recognized wetland types (Johnson and Smith 2006). The state contains all or part of 12 HUC6 watersheds (Fig. 5) that contribute to three of the 18 major watersheds delineated in the continental USA by the U.S. Geological Survey 2-digit Hydrologic Unit Codes (HUC) 02 (Mid-Atlantic), 04 (Great Lakes), and 05 (Ohio River) (Seaber et al. 1987, Endreny 2005). These 12 HUC6 drainage basins (Fig. 5) encompass all or part of 51 smaller HUC8 drainages (Fig. 6). An elaborate canal system constructed from the 1820’s to the mid-1800’s, including the famous Erie Canal, has connected nearly all the major drainages of the state for the past 125 years (Finch 1925, Thompson 1980, Zembrzuski and Gannon 1986). Combined with the increased recreational use, this has greatly aided the spread of many invasive species (Endreny 2005, Marsden and Hauser 2009).

Continuous threats to aquatic habitats in the state, include the effects of urbanization and development, non-point source pollution, organic and industrial pollution, use of de-icing agents on roadways, climate warming, impoundments, siltation, agriculture, forest management and the utilization, excavation, and filling of springs and small seeps (Blasius and Merritt 2002, Morse et al. 2006, Reiss et al. 2016, Smith et al. 2018). A comprehensive assessment of water quality using biological indicators by the New York State Department of Environmental Conservation Stream Biomonitoring unit concluded that 45% of streams and rivers assessed of New York State were classified as non-impacted, 41% as slightly impacted, 13% as moderately impacted, and 1% as severely impacted (Bode et al. 2004). A more recent update on the status of surface water quality trends over a fourty year period in New York State indicates a switch from point to non-point sources of pollution (Smith et al. 2018).

## Materials and methods

### Field methods

Fieldwork conducted mainly by the first two authors spanned from 2008 through 2023 and was scheduled to coincide with adult presence of targeted taxa. Standard collection methods included the use of beating sheets, sweep nets, aerial nets to dislodge adults from riparian vegetation, visual searching along bridges to obtain winter-emergent species, ultraviolet light traps on warm summer evenings mainly for Perlidae, and rearing of immatures to the adult stage in a Frigid Units Living Stream (Frigid Units, Inc., Toledo Ohio, USA) (see below; DeWalt et al. 2015, Jackson et al. 2019). Location coordinates were formatted to latitude and longitude decimal degrees using a Garmin GPS unit. Most field-collected specimens were preserved on site with 70-95% ethanol. Adult male stoneflies are usually required for accurate species identification (Stark and Armitage 2000, Stark and Armitage 2004, Grubbs and Baumann 2023, Ross and Ricker 1971). Because males of several genera of Perlidae (i.e., *Perlesta* Banks, 1906 and *Acroneuria* Pictet, 1841) and *Isoperla* have an aedeagus that needs to be fully everted to ensure positive identification, they were often kept alive for several hours to several days prior to processing in the laboratory.

### Lab methods

Because adults of many taxa can be difficult to locate in the field, late instar field collected larvae were reared in the laboratory at Colorado State University and SUNY Plattsburgh. Date of emergence was recorded for all reared taxa. Whenever possible, adult males and females were kept alive for several days in small containers to enable mating and egg production in females to enhance identifications of *Acroneuria*, *Isoperla*, *Perlesta*, and *Neoperla* Needham, 1905. The containers were kept at room temperature (10-22ºC) and a small amount of stream water (1-2 drops) was replenished daily as drinking water and humidity source. The aedeagus of live *Acroneuria*, *Isoperla*, and *Perlesta* were partially to fully-extruded under a stereo microscope. Once the eversion was completed, specimens were then transferred to a hot water bath to prevent retraction before subsequent preservation in ethanol.

When possible, specimens were identified to species using pertinent literature and by comparison to existing museum specimens for verification. If necessary, specimens were sent to taxonomic experts for verification. Changes in taxonomic nomenclature (i.e., new combinations, synonymies, etc.) were checked against Plecoptera Species File (DeWalt et al. 2024). Additional specimens were borrowed and examined from 23 institutional collections and government agencies to verify literature records of stoneflies from the state (Table 2).

### Data management, mapping, and faunistic analyses

Occurrence data were also integrated from museum specimen records and valid literature records (Table 3). Collection localities from these sources were georeferenced using Acme Mapper 2.2 (https://mapper.acme.com). Specimen data integrated from several sources and cleaned using Google Sheets, Microsoft Excel, and OpenRefine (Delpeuch et al. 2024), confirming that all data fit the DwC-A standard format (Wieczorek et al. 2012). Data were validated and mapped to this format before being published to the Global Biodiversity Information Facility (Myers L, Kondratieff B, Grubbs S, Pett L, DeWalt R E, Mihuc T, Hart L (2025). Distributional and species richness patterns of the stoneflies (Insecta, Plecoptera) in New York State: occurrence dataset. Version 1.1. Biodiversity Data Journal. Occurrence dataset https://doi.org/10.15468/hkum7k accessed via GBIF.org on 2025-08). Data also included HUC8, HUC12, and USEPA Level III and Level IV Ecoregions.

Species records were spatially joined with USGS HUC8 watershed boundaries (Fig. 5; Steeves and Nebert 1994) and USEPA Level IV Ecoregions (Fig. 3; US Environmental Protection Agency 2013). Elevation data were extracted from 30-m Digital Elevation Models using the zonal statistics as a table function in ArcGIS Pro 3.1.1. Distributional maps were prepared using ArcGIS 10.8.1. Specimen data accrued during this project are available (Suppl. material 1).

### Species accumulation curves

To better understand richness patterns and to evaluate our sampling efforts across the state, we developed species accumulation curves at multiple spatial scales including the total extent of New York State, HUC8 watersheds, 100-meter elevation bands, and USEPA Level IV Ecoregions. Species accumulation curves illustrate the relationship between observed species' richness and each subsequent sampling event (Gotelli and Colwell 2010). When repeated sampling events produced marginal increases in observed richness, we deemed sampling effort sufficient. Conversely, when repeated sampling events produce significant increases in observed richness, more sampling was warranted. The significance of these increases was interpreted through confidence intervals calculated from several permutations that randomize sampling sites and subsequent observed increases in species' richness.

To calculate the statewide accumulation curve and confidence intervals, we first compiled an abundance matrix where each row was a unique collection site (1375 sites), each column was a species (127 species), and each cell contained its presence or absence. We defined the number of permutations as 100 and then used the *poolacum* function in the R Package *vegan* (Oksanen et al. 2022) to produce 100 estimates of species richness at each growing level of sample size. This species accumulation analysis was repeated for each unit of sampling area in several different spatial scales (HUC8 watersheds, 100 m elevation bands, and USEPA Level IV Ecoregions). The specpool function in the R package vegan (Oksanen et al. 2022) was used to calculate the Chao2 nonparametric estimation of species richness. The estimation is appropriate for incidence data (Gotelli and Colwell 2010) and accounts for undetected species using the frequency of singletons and doubletons (Chao 1987).

## Checklists

### A checklist of stoneflies (Plecoptera) in New York State

#### Capniidae

Banks, 1900

B3F89F15-36C6-5347-9BEA-76E6BDB3E3B4

##### Notes

Capniidae are commonly referred to as Snowflies (Stark et al. 1998, Stark et al. 2012). They are amongst the first to emerge as adults during the late autumn and winter, and together with Taeniopterygidae are referred to historically as "winter stoneflies". Eighteen species and three genera of Capniidae are known from New York, some of which have been infrequently reported. A recent (since 2000) taxonomic treatment of the eastern North American species of Capniidae does not yet exist. Instead, we have to rely on a combination of recent and older literature. For example, Ross and Ricker (1971) provided the most recent taxonomic review of the common winter stonefly genus, *Allocapnia* Claassen, 1928, including evolutionary and post-Pleistocene dispersal hypotheses, but several species have been described during the intervening 50+ years. Additional important taxonomic references to identify New York taxa include Hitchcock (1974), Nelson and Baumann (1987), Stark and Baumann (2004), Stark and Kondratieff (2012), and Burton (2019). It is likely that all *Allocapnia* larvae undergo a diapause deep in the sediments of streams, a condition that breaks in autumn allowing growth to continue through the winter (Pugsley and Hynes 1985).

**Figure 7.**
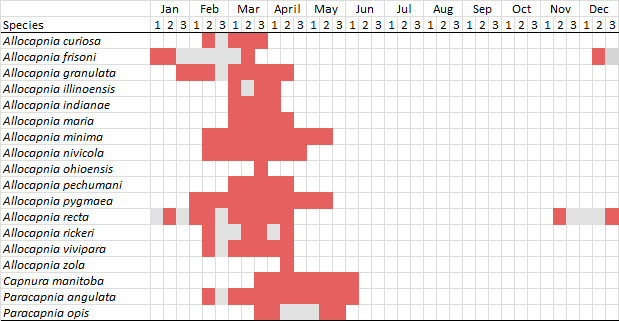
Adult flight period for 18 Capniidae species in New York State. Red fill indicates positive adult collections while gray shaded areas indicate adults are likely present but not yet reported.

**Figure 8.**
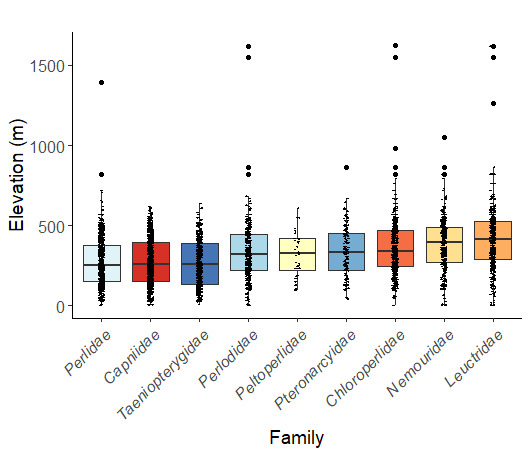
Elevation box plot for stonefly families in New York State. Boxes indicate interquartile range, horizontal line in box represents median elevation, and outlliers are depicted with large circles.

**Figure 9.**
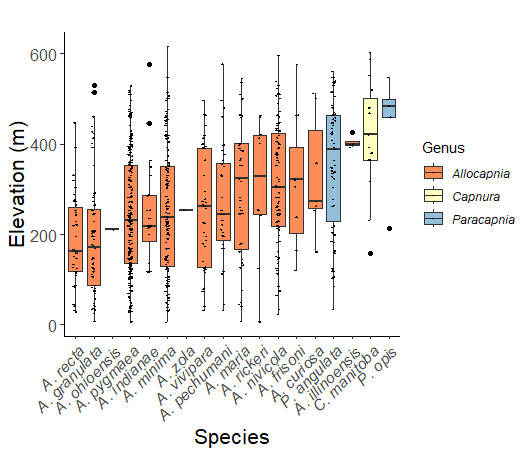
Elevation box plot for 18 Capniidae species in New York State. Boxes indicate interquartile range, horizontal line in box represents median elevation, and outliers are depicted with large circles.

Adult collection dates for this family range from mid-November through early June (Fig. 7). Capniidae in New York occupy a wide range of elevations from 5-617 m (Fig. 8, Fig. 9). *Allocapnia* are generally present at lower elevations, including *Allocapniarecta* and *A.granulata*, throughout the state (Fig. 9). The widest ranging elevations are reported for the most commonly collected species include *A.minima*, *A.nivicola*, and *A.pygmaea* (Fig. 9). The narrow range of reported elevations for *A.ohioensis*, *A.zola*, and *A.illinoensis* are due to the limited number of available records for each of these uncommon species (Fig. 9).

#### Allocapnia curiosa

Frison, 1942

D806A793-08B1-5B79-9E23-A19C46FF7901

##### Notes

*Allocapniacuriosa* is commonly referred to as the Peculiar Snowfly (Stark et al. 2012). This species has been reported from New York south to Kentucky and Virginia (Ross and Ricker 1971, DeWalt et al. 2024). Ross and Ricker (1971) reported this species from high-gradient, cool semi-montane streams of moderate size, including seven New York localities (their fig. 98) with emergence dates ranging from late January to mid-April but with no detailed collection data. In New York, adults have been collected from mid-February through late March (Fig. 7). This species has been reported in New York from streams at lower elevations (161-512 m asl; Fig. 9) from Level IV Ecoregions Catskill High Peaks (58y), Catskills Transition (60c), and Unglaciated High Allegheny Plateau (62d) (Fig. 10a).

#### Allocapnia frisoni

Ross & Ricker, 1964

8AFB759B-D679-5D5C-BDD6-2359B9017126

##### Notes

This species is commonly known as the Evansville Snowfly (Stark et al. 2012). The distribution of this species extends from New York southwest mainly through the Appalachian Mountains to Tennessee (Ross and Ricker 1971, DeWalt et al. 2024). Ross and Ricker (1971) recorded the presence of this species from six localities around Tompkins County (their fig. 93), however, no data was associated with these records. Adults of this species have been collected from mid-December through mid-March (Fig. 7). This species reaches its northern limits in streams of moderate elevation (121-577 m asl) in New York (Fig. 9) with reports from Level IV Ecoregions Glaciated Low Allegheny Plateau (60a), Finger Lakes Uplands and Gorges (60d), Cattaraugus Hills (60f), Low Lime Drift Plain (61c), and Unglaciated High Allegheny Plateau (62d) (Fig. 10b).

#### Allocapnia granulata

(Claassen, 1924)

C207F93C-F360-5D74-8896-02E52769B545

##### Notes

*Allocapniagranulata* is commonly referred to as the Common Snowfly (Stark et al. 2012). This widespread, common species has been recorded from southern Manitoba east to Quebec and south to Texas and USA Gulf Coastal states (Ross and Ricker 1971, DeWalt et al. 2024) and occurs in a wide range of streams and river habitats with varying degrees of flow and turbidity. This species distribution extends into northern New York along the Lake Champlain (Myers et al. 2011) and St. Lawrence River drainages. Ross and Yamamoto (1967) and Ross and Ricker (1971) each provided thorough discussions of post-Pleistocene dispersal pathways. As in other members of this genus, Harper and Hynes (1970) and Finni and Chandler (1977) found that larvae of *A.granulata* undergo an apparent diapause during the summer. In New York, adults of this species were collected late-January through early April (Fig. 7). *Allocapniagranulata* was found at a wide range of elevations (7-529 m asl) in streams and rivers throughout the state (Fig. 9, Fig. 10c).

#### Allocapnia illinoensis

Frison, 1935

C4E1E8CA-8389-5308-A599-D1416755D035

##### Notes

This species is commonly known as the Illinois Snowfly (Stark et al. 2012). Isolated populations of this rare species have been reported mainly from eastern Canada, south to Virginia, and west to Illinois (Ross and Ricker 1971, DeWalt et al. 2024). Ross et al. (1967) discussed the post-glacial colonization of this species into eastern Canada. Ross and Ricker (1971) provided a distributional map of this species that included three localities in eastern New York State (their fig. 97) yet without precise locality information. Myers et al. (2011) provided the most recent reports of this species from a first order low gradient stream with a substrate composed primarily of sand and cobble, with moss covering some of the larger in-stream substrates. Harper and Harper (1983) reported that *A.illinoensis* “dominate” in small streams of southern Quebec. Webb (2002) presented evidence that this species has been extirpated from Illinois. In New York, adults of this species have been collected from early March through early April (Fig. 7) but infrequently from streams and rivers of moderate elevation 392-427 m asl (Fig. 9). This species is known from four unique locations in Level IV Ecoregions Eastern Adirondack Foothills (58ac), Central Adirondacks (58ad), Glaciated Low Allegheny Plateau (60a), and Mohawk Valley (83f) (Fig. 10d).

#### Allocapnia indianae

Ricker, 1952

B6BEDFA4-9BB2-5185-ADF3-44939757E5C4

##### Notes

This species is commonly referred to as the Indiana Snowfly (Stark et al. 2012). *Allocapniaindianae* is known from the lower Ohio River valley region encompassing Kentucky, Indiana, Ohio, and West Virginia, plus a disjunct set of localities in New York (Ross and Ricker 1971, DeWalt et al. 2024). In New York, this species is known only from historical reports with the most recent collection in 1966. Ross and Freytag (1967) reviewed the current distribution and probable post-Pleistocene dispersal routes of this species and its closely related sister species *A.ohioensis*. In New York, adults have been collected from early March to early April (Fig. 7) from small streams at 117-577 m asl (Fig. 9). Verified reports are available from Level IV Ecoregions Catskill High Peaks (58y), Glaciated Low Allegheny Plateau (60a), Finger Lakes Uplands and Gorges (60d), Ontario Lowlands (83c), and Mohawk Valley (83f) (Fig. 10e).

#### Allocapnia maria

Hanson, 1942

51CDFD48-0685-5741-AF8F-96F0C8E6DC69

##### Notes

*Allocapniamaria* is commonly known as the Two-knobbed snowfly (Stark et al. 2012). This species has been reported from eastern Canada and south to Virginia (Ross and Ricker 1971, DeWalt et al. 2024). Ross and Ricker (1971) reported this species from several locations near Ithaca and in the Catskill Mountains (their Fig. 99) yet without precise locality data. Hanson (1960) was the first to report hybridization of this species with *A.minima* when species are sympatric. Additionally, Ross and Ricker (1971) noted that this species also hybridizes with *A.pechumani* further north in New Brunswick (their fig. 99). In New York, adults of this species are active from early March through mid-April (Fig. 7) and reported from numerous lowland localities with a distribution that extends to the north along the low valleys surrounding the Adirondack Mountains (Fig. 10f; Myers et al. 2011). This species is probably more common in the southern and western portions of the state than collections have indicated.

#### Allocapnia minima

(Barnston, 1848)

51895397-8C43-5397-99B9-699C98C93A00

##### Notes

*Allocapniaminima* is commonly referred to as the Boreal Snowfly (Stark et al. 2012). This species is a common inhabitant of the Northern Boreal Forest. Records extend in Canada from the island of Newfoundland west to Ontario and in the USA from Maine west to Minnesota (Ross and Ricker 1971, DeWalt et al. 2024). This species is known to hybridize in localized populations with closely related *A.maria* (Hanson 1960, Ross and Ricker 1971). Adults of this species are present in New York from mid-February through late May (Fig. 7). This species occupies a wide range of elevations from 6-616 m asl (Fig. 9) and is common and abundant in the Adirondacks and surrounding valleys (Fig. 11a) and often associated with *A.pygmaea* in Adirondack rivers (Myers et al. 2011). This species was also recently collected and reared from small headwater Adirondack streams.

#### Allocapnia nivicola

(Fitch, 1847)

8EFE9CA7-0424-5F76-8AFA-26A40F2BD2A1

##### Notes

This species is commonly referred to as the Brook Snowfly (Stark et al. 2012). The range of this species extends from New Brunswick, Nova Scotia, and Quebec southwest to Alabama and west to Illinois and Wisconsin (Ross and Ricker 1971, DeWalt et al. 2024). Adult flight period dates for this common and widespread species extend from mid-February to mid-April (Fig. 7). In New York, this species is common and has been reported from small rocky streams at 7-545 m asl (Fig. 9) throughout the state (Fig. 11b).

#### Allocapnia ohioensis

Ross & Ricker, 1964

EF7251DE-A6E7-5567-B488-C6ECBE8F5A9D

##### Notes

*Allocapniaohioensis* is commonly known as the Ohio Snowfly (Stark et al. 2012). The range of this species is similar to that of *A.indianae*, including disjunct New York populations (Ross and Ricker 1971, DeWalt et al. 2024). This species has been previously reported from small, gravel bottom streams (Ross and Ricker 1971). In New York, this species appears to be more uncommon than the previously mentioned sister species, *A.indianae*. A single record is available from New York, collected in late March 1960 (Fig. 7) from a low elevation (210 m asl) (Fig. 9) stream in Level IV Ecoregion 83f Mohawk Valley (Fig. 11c).

#### Allocapnia pechumani

Ross & Ricker, 1964

5A548C21-A926-524D-BC89-0008FD2D83D5

##### Notes

This species commonly referred to as the St. Lawrence Snowfly (Stark et al. 2012), has been reported patchily from Quebec and New Brunswick southwest to Ohio (Ross and Ricker 1971, DeWalt et al. 2024). This species was named in honor of the late Dr. Verne Pechuman, a tabanid fly expert who conducted research at Cornell University. Although this species is widely distributed across much of the southern areas of the state, in northern areas it appears it is restricted to lower elevations of Level IV Ecoregions Champlain Lowlands (83b), Ontario Lowlands (83c), and Mohawk Valley (83f) (Fig. 11d) and often in association with *A.maria*. In New York, adult collections occur from early March through mid-April (Fig. 7) in small streams with elevations ranging from 32-577 m asl (Fig. 9).

#### Allocapnia pygmaea

(Burmeister, 1839)

BA49702F-4C7B-5A69-8048-02A58A1D64FA

##### Notes

*Allocapniapygmaea* is commonly known as the Pygmy Snowfly (Stark et al. 2012). This widespread and often abundant species ranges from southeastern Canada west to Iowa and North Dakota and southwest to Missouri and Tennessee (Ross and Ricker 1971, DeWalt et al. 2024). This species inhabits small to medium sized streams, with gravel and rocky substrates that remain cool throughout much of the summer (Ross and Ricker 1971). The larvae apparently diapause in the hyporheic zone of streams during the summer months (Pugsley and Hynes 1985). Adult collection dates for *A.pygmaea* in New York range from early February through mid-May (Fig. 7). This species has been documented from a wide range of stream sizes and elevations (5-530 m asl, Fig. 9), and is common throughout the state (Fig. 11e).

#### Allocapnia recta

(Claassen, 1924)

CC920EA3-0281-5573-8BDD-6D037015EF08

##### Notes

This widespread species commonly referred to as the Eastern Snowfly (Stark et al. 2012), ranges from southeastern Canada, southwest to Louisiana and west to Illinois and Wisconsin (Ross and Ricker 1971, DeWalt et al. 2024). This is a common member of the genus and is found in both perennial and intermittent spring-fed streams (Hitchcock 1974). Grubbs et al. (2006) studied the life history of *A.recta* in a central Kentucky karst headwater stream and found that larvae entered an apparent diapause throughout the summer months. In New York, adults are active from early November through mid-April (Fig. 7). This species was collected in New York from small streams at elevations ranging from 33-447 m asl (Fig. 9). This species is distributed across the state but is primarily restricted in northern regions to low elevation valleys surrounding mountainous areas of the Adirondacks, Catskills, and Tug Hill Plateau (Fig. 11f).

#### Allocapnia rickeri

Frison, 1942

958AA11C-3A13-5479-8C6D-8D5D23CDFC57

##### Notes

*Allocapniarickeri* is commonly known as the Midwest Snowfly (Stark et al. 2012). This widespread species is known from Quebec and Ontario south to Georgia, Alabama, and Mississippi, southwest to Kansas and Oklahoma, and west to Minnesota. This species is common throughout its range, especially in the Midwestern USA (Ross and Ricker 1971, DeWalt et al. 2024). Historical and recent collections of adults have occurred in the state from mid-February through mid-April (Fig. 7). In New York, records are available at elevations ranging from 6 to 464 m asl (Fig. 9), from streams in Level IV Ecoregions Glaciated Low Allegheny Plateau (60a), Finger Lakes Uplands and Gorges (60d), Glaciated Allegheny Hills (60e), Cattaraugus Hills (60f), Low Lime Drift Plain (61c), Hackensack Meadowlands (64g), and Erie/Ontario Lake Plain (83a) (Fig. 12a).

#### Allocapnia vivipara

(Claassen, 1924)

D8BF5FBA-FD1F-5639-83EE-B34F8C4F43FC

##### Notes

*Allocapniavivipara* is commonly referred to as the Shortwing Snowfly (Stark et al. 2012). This species is distributed in a diagonal band from southern Ontario and Quebec west to Iowa, Kansas, Minnesota, Nebraska, and Oklahoma (Ross and Ricker 1971, DeWalt et al. 2024). Males are apterous and have been reported from a wide range of stream sizes and can be especially abundant in nutrient rich streams (Ross and Ricker 1971). In New York, adults of *A.vivipara* have been collected from mid-February through mid-April (Fig. 7) from streams and rivers in agricultural areas at elevations of 32-496 m asl (Fig. 9). This species distribution is primarily centered along the Great Lakes Plain and other lowland valleys in the state, but it is still able to colonize some areas of higher elevation in Level IV Ecoregion Northern and Western Adirondack Foothills (58ab) (Fig. 12b).

#### Allocapnia zola

Ricker, 1952

EACF22C2-FA55-5E12-A0A4-CF7921C87FCE

##### Notes

This species is commonly known as the Ash Snowfly (Stark et al. 2012), occurs in a diagonal band flanking the western edge of the Appalachian Mountains from New Brunswick southwest to Tennessee (Ross and Ricker 1971, DeWalt et al. 2024). Ross and Ricker (1971) reported this species from medium-sized streams with rocky substrates, with collection dates extending from late December to early April. In New York, this species is known from only one historical collection event on April 13, 1937 (Fig. 7) from a stream at 253 m asl (Fig. 9) from Level IV Ecoregion Erie/Ontario Lake Plain (83a) in western New York (Fig. 12c). Further collecting in western New York should reveal additional populations.

#### Capnura manitoba

(Claassen, 1924)

18F934AD-E042-55BB-BD3C-EC1B4AF81CA9

##### Notes

*Capnuramanitoba* is commonly referred to as the Manitoba Snowfly (Stark et al. 2012). This species ranges in Canada from Quebec and New Brunswick south to the USA from Maine west to Wisconsin (Nelson and Baumann 1987, DeWalt et al. 2024). Harper and Hynes (1972) suggested that larvae of *C.manitoba* undergo a summer diapause similar to *A.pygmaea*. Little is known about the habitat and microdistribution of the larvae of this species (Stewart and Stark 2002). In New York, adults were collected from mid-February through early June (Fig. 7) at 157-602 m asl (Fig. 9) from small, pristine spring-fed streams in Level IV Ecoregions Acid Sensitive Adirondacks (58aa), Northern and Western Adirondack Foothills (58ab), Eastern Adirondack Foothills (58ac), Central Adirondacks (58ad), Glaciated Reading Prong/Hudson Highlands (58i), Champlain Lowlands (83b), and Mohawk Valley (83f) (Fig. 12d).

#### Paracapnia angulata

Hanson, 1961

5D9A0AE3-B7E8-5C52-984D-0CB4E1B535B9

##### Notes

This species is commonly known as the Angulate Snowfly (Stark et al. 2012). *Paracapniaangulata* is widely distributed throughout North America ranging from Newfoundland-Labrador west to Saskatchewan, south along the Appalachian Mountains to north Georgia, and southwest across the Midwestern USA to Colorado and Wyoming (Stark and Baumann 2004, DeWalt et al. 2024). Prior to Hanson (1961), some historical records of *P.opis* and *Capniavernalis* (Newport, 1848) are referable to this species. Harper and Hynes (1972) reported a synchronous emergence of *P.angulata* in mid-April and direct hatching of the eggs in May, with first instar larvae initially appearing in mid-June. Myers et al. (2011) reported this species from all four watersheds draining the Adirondack Park. In New York, adult collection dates extend from mid-February through early June (Fig. 7). This species has been collected commonly from streams at 34-561 m asl (Fig. 9) throughout the state (Fig. 12e).

#### Paracapnia opis

(Newman, 1839)

18371517-8142-550C-9AC4-D0791631D332

##### Notes

*Paracapniaopis* is commonly known as the Northeast Snowfly (Stark et al. 2012). This species is distributed from Quebec and Newfoundand-Labrador south to an isolated locality in West Virginia and west to Minnesota (Stark and Baumann 2004, DeWalt et al. 2024). This species appears to be more common north of New York (Stark and Baumann 2004). Delucchi and Peckarsky (1989) supposedly studied the life history of *P.opis* in an intermittent stream in Tompkins County and found that larvae survive the dry season by migrating to the hyporheic zone or riffles that remain wet throughout the season. However, because the only available records for New York are from Level IV Ecoregions Acid Sensitive Adirondacks (58aa), Central Adirondacks (58ad), and Champlain Lowlands (83b) (Fig. 12f), it is likely that their study pertains to the more common *P.angulata*. In New York, adults have been reported from late March to late May (Fig. 7) from cold streams and rivers at 214-548 m asl (Fig. 9).

#### Leuctridae

Klapálek, 1905

E1EA7751-3643-5833-9897-EC1A97DF75FA

##### Notes

**Figure 13.**
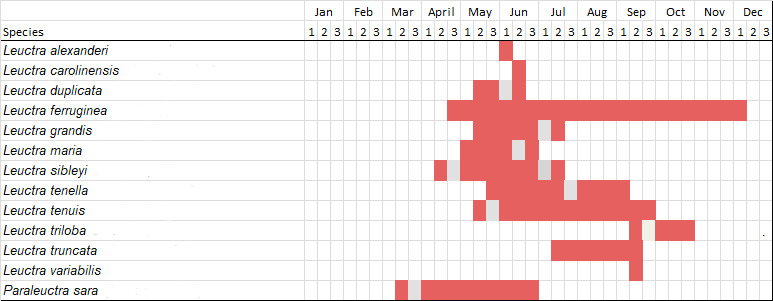
Adult flight period for 13 Leuctridae species in New York State. Red fill indicates positive adult collections while gray shaded areas indicate adults are likely present but not reported.

**Figure 14.**
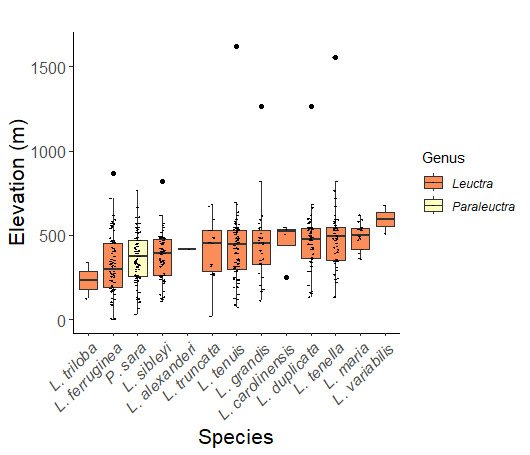
Elevation box plot for 13 Leuctrdae species in New York State. Boxes indicate interquartile range, horizontal line in box represents median elevation, and outliers are depicted with large circles.

Leuctridae are commonly referred to as Needleflies (Stark et al. 1998, Stark et al. 2012) and can be collected in large numbers as adults, especially in forested headwater streams during late spring and throughout the summer. Two genera and 13 species of Leuctridae occur in New York. Similar to Capniidae, there is not a recent review of the eastern North American species of Leuctridae, especially for species-rich *Leuctra* Stephens, 1836. The treatment of this family by Hitchcock (1974) in Connecticut, the Harper and Harper (1997) preliminary report on *Leuctra*, and the treatments of the *L.duplicata* and *L.tenuis* species groups by Grubbs and Wei (2017) and Grubbs (2015), respectively, were useful taxonomic references over the course of this study. Scott A. Grubbs is currently revising *Leuctra* and taxonomic changes are likely. Adult flight periods for this family range from mid-March through early December (Fig. 13). This family has been reported from a wide range of elevations from 2-1610 m asl (Fig. 8, Fig. 14). *Leuctraduplicata*, *L.grandis*, *L.tenuis*, and *L.tenella* have been reported from the widest range of elevations (Fig. 14). The narrowest ranges were reported for species reported from relatively few locations including, *L.alexanderi*, *L.maria*, *L.triloba*, and *L.variabilis*.

#### Leuctra alexanderi

Hanson, 1941

85E3EF6D-D3B1-584D-9C11-5D5A95AC0682

##### Notes

*Leuctraalexanderi* is commonly known as the Anakeesta Needlefly (Stark et al. 2012). This is an Appalachian-distributed species previously reported from Ohio and Pennsylvania south to Georgia (DeWalt et al. 2016, DeWalt et al. 2024). A single adult male was collected from New York on June 6, 1991 (Fig. 13) at approximately 416 m asl (Fig. 14) from a stream in Tioga County in Level IV Ecoregion Glaciated Low Allegheny Plateau (60a) (Fig. 15a). This record represents a new state record for New York and a small northward extension for this species (Fig. 15a).

#### Leuctra carolinensis

Claassen, 1923

3DED7B41-E618-5F65-B7D2-EB1525F9AD30

##### Notes

This species is commonly referred to as the Carolina Needlefly (Stark et al. 2012). Its current known range extends from Georgia and Mississippi north to Connecticut (Grubbs 2015, DeWalt et al. 2024). Adults of this species were collected in New York during mid-June (Fig. 13). Reported elevations for this species in the state range from 248-548 m asl (Fig. 14). These reports are based upon 1941 historical collections from Level IV Ecoregions Acid Sensitive Adirondacks (58aa) and Central Adirondacks (58ad) (Fig. 15b), representing a new state record.

#### Leuctra duplicata

Claassen, 1923

8F4AAC71-56DE-5770-AAEF-C055849CAF14

##### Notes

*Leuctraduplicata* is commonly known as the Atlantic Needlefly (Stark et al. 2012), and is distributed from southeastern Canada south to North Carolina (Grubbs and Wei 2017, DeWalt et al. 2024). Harper (1990a) found that larvae of *L.duplicata* displayed a fast, univoltine life cycle in an intermittent stream in southern Quebec. Turner et al. (1996) reported larvae and adults of *L.duplicata* and *L.maria* from pitcher plants (*Sarraceniapurpurea* L.), although it is likely that these individuals entered the plants during emergence from the surrounding bog habitat (Stewart and Stark 2002). All across New York, adults have been collected from mid-May to mid-June (Fig. 13) and commonly from spring seeps and smaller streams at 135-1262 m asl (Fig. 14) across the state (Fig. 15c).

#### Leuctra ferruginea

(Walker, 1852)

405E6CFE-38E6-5391-B828-5F0329FD3195

##### Notes

*Leuctraferruginea* is commonly referred to as the Eastern Needlefly (Stark et al. 2012). This widespread species is known from most Canadian provinces and USA states east of Hudson Bay and the Mississippi River, respectively. Further west, *L.ferruginea* has been reported from Saskatchewan and Minnesota south to Louisiana (DeWalt et al. 2024). This species has been reported from a wide range of stream sizes, with semivoltine life cycles in smaller streams and univoltine life cycles in larger rivers (Harper 1973a). We reported adult collection dates from the state ranging from late April to early December (Fig. 13). We documented this species from elevations ranging from 2-866 m asl (Fig. 14) and a wide range of stream sizes, including coastal streams on Long Island (Fig. 15d).

#### Leuctra grandis

Banks, 1906

E37B154B-DC63-5C6C-954B-AB5207B19245

##### Notes

This Appalachian-distributed species is commonly referred to as the Grand Needlefly (Stark et al. 2012) and has been reported from New Brunswick in Canada and in the USA from Maine southwest to Alabama (DeWalt et al. 2024). Adult collection dates for the state range from mid-May to mid-July (Fig. 13). In New York, this species was collected from medium-sized streams from a broad range of elevations from 113-1262 m asl (Fig. 14). This species is common in cold streams and rivers throughout the Level III Ecoregions Northeast Highlands (58), Northern Allegheny Plateau (60), and Eastern Great Lakes Lowlands (83) (Fig. 15e), and is often associated with the more common *L.sibleyi*.

#### Leuctra maria

Hanson, 1941

CF0E12F0-650E-58B1-9B58-8C99E3638880

##### Notes

*Leuctramaria* is commonly known as the Northeastern Needlefly (Stark et al. 2012). This is an uncommonly collected species from Ontario and Quebec south to West Virginia (Grubbs and Wei 2017, DeWalt et al. 2024). This species inhabits small to medium-sized streams and lake outlets, many of which are dry during summer and early autumn (Peter Harper, personal communication, October 24, 2007). Adult collection dates ranged from early May to late June (Fig. 13). In New York, this species was found infrequently from marshy habitats near streams at elevations of 356-621 m asl (Fig. 14) in Level IV Ecoregions Acid Sensitive Adirondacks (58aa), Northern and Western Adirondack Foothills (58ab), Eastern Adirondack Foothills (58ac), Central Adirondacks (58ad), and Mohawk Valley (83f) (Fig. 15f).

#### Leuctra sibleyi

Claassen, 1923

63B6F2F7-8224-568B-8FE2-3C1C4A2233AF

##### Notes

This species is commonly referred to as the Brook Needlefly (Stark et al. 2012). The distribution of this species is from most Canadian provinces and USA states east of the Hudson Bay and Mississippi River, respectively (DeWalt et al. 2024). Masteller (1983) reported peak emergence of this species during mid-May from Six-Mile Creek, in Erie County, Pennsylvania. In New York, adults of this species have been reported from mid-April to mid-July (Fig. 13). Reported elevations for *L.sibleyi* in the state range from 111-821 m asl (Fig. 14). This species is common in New York, with most records to date from the Level III Ecoregions Northeastern Highlands (58), Northern Allegheny Plateau (60), Erie Drift Plain (61), and Eastern Great Lakes Lowlands (83) (Fig. 16a).

#### Leuctra tenella

Provancher, 1878

01D1E345-EE7B-5FF3-9186-48083D6E26D7

##### Notes

*Leuctratenella* is commonly known as the Broad-Lobed Needlefly (Stark et al. 2012) and is known from mainland Labrador west to Minnesota and southwest along the Appalachian Mountains to Georgia (Grubbs 2015, DeWalt et al. 2024). Harper and Hynes (1971a) reported this species from small spring-fed streams. In New York, adults of *L.tenella* have been reported from mid-May to early September (Fig. 13) from a wide range of elevations (135-1551 m asl; Fig. 14). This species was frequently collected from Level III Ecoregions Northeastern Highlands (58) and the Eastern Great Lakes Lowlands (83) and is likely more common than collections indicate in western New York (Fig. 16b).

#### Leuctra tenuis

(Pictet, 1841)

4B7EA798-6CA9-53E6-B47A-FE67A4DE273E

##### Notes

*Leuctratenuis* is commonly referred to as the Narrow-Lobed Needlefly (Stark et al. 2012). This species is distributed extensively across eastern North America, except for the USA Gulf Coastal region, and west to Oklahoma (Grubbs 2015, DeWalt et al. 2024). *Leuctratenuis* has been associated with larger streams and rivers throughout Quebec and Ontario (Harper and Hynes 1971a). Masteller (1983) reported peak emergence of *L.tenuis* in July from a stream in Pennsylvania. Adults of this species are active from mid-May through late September (Fig. 13). *Leuctratenuis* has been reported from a wide range of elevations from 72-1620 m asl (Fig. 14) and is common and abundant throughout the state (Fig. 16c).

#### Leuctra triloba

Claassen, 1923

F78C4740-8DBE-5F2A-A55D-77F09494E96C

##### Notes

This species is commonly referred to as the Three-Lobed Needlefly (Stark et al. 2012), and has been previously reported from Quebec and New York south to Alabama and Florida (Grubbs 2015, DeWalt et al. 2024). Harper and Harper (1997) reported a fall emergence of this species, but no information is available on the habitat or microdistribution of this species. Our limited data from historical collections in New York indicate a similar emergence with dates ranging from mid-September through October (Fig. 13). Elevations for *L.triloba* in New York range from 125-340 m asl (Fig. 14). This species appears to be relatively uncommon at its northernmost range limits. A single record is available from southern Quebec (Harper and Hynes 1971a) and in New York State this species has not been recollected since its original description from the type locality in Ithaca and another nearby locality in the late 1800's. Both state records reside in Level IV Ecoregion Finger Lakes Uplands and Gorges (60d) (Fig. 16d). Less frequent collection efforts during autumn and early winter may account for the paucity of records from New York.

#### Leuctra truncata

Claassen, 1923

89781B9F-83F2-5516-8FD7-74C7216407F0

##### Notes

This is an Appalachian-distributed species commonly known as the Truncate Needlefly (Stark et al. 2012), and is known from Quebec and the island of Newfoundland south to North Carolina (DeWalt et al. 2024). Harper and Hynes (1971a) reported this species from small cold streams in southern Quebec, with adult emergence occurring late in the summer. Our collections from New York indicate a similar period of adult activity from mid-July through mid-September (Fig. 13). In New York, *Leuctratruncata* has been reported from elevations ranging from 20-684 m asl (Fig. 14) from Level IV Ecoregions Acid Sensitive Adirondacks (58aa), Northern and Western Adirondack Foothills (58ab), Eastern Adirondack Foothills (58ac), Central Adirondacks (58ad), Hudson Valley (59i), and Catskills Transition (60c) (Fig. 16e).

#### Leuctra variabilis

Hanson, 1941

6670B28C-3DE9-5EF0-A1B9-89EBB7AD356A

##### Notes

*Leuctravariabilis* is commonly referred to as the Variable Needlefly (Stark et al. 2012), and is known from Maine south to North Carolina and Tennessee (Grubbs 2015, DeWalt et al. 2024). Turner et al. (1996) reported this species from a bog in Maryland, with collection dates ranging from mid-November to mid-December. Hitchcock (1974) reported the emergence of this species from Connecticut streams ranged from August to late November. In New York, adults have been collected in mid-September (Fig. 13) at elevations ranging from 509-677 m asl (Fig. 14) from small streams and seeps in Level IV Ecoregion Central Adirondacks (58ad) and Catskill High Peaks (58y) (Fig. 16f).

#### Paraleuctra sara

(Claassen, 1937)

85622A1E-28C5-5DA5-9CF6-062570C1E6EF

##### Notes

*Paraleuctrasara* is commonly known as the Appalachian Needlefly (Stark et al. 2012). This species is known from Ontario and southeastern Canada south along the Appalachian Mountains to Georgia and Alabama, and also with a westward extension through Ohio to Indiana (DeWalt and Grubbs 2011, DeWalt et al. 2016,Stark and Kyzar 2001, DeWalt et al. 2024). Harper and Hynes (1971a) reported this species from streams and smaller rivers in Quebec and Ontario. This species is commonly collected in the early spring. Adults are present in New York from mid-March through mid-June (Fig. 13). Reported elevations for this species in New York ranged from 33-764 m asl (Fig. 14) with a broad state-wide distribution (Fig. 17a).

#### Nemouridae

Billberg, 1820

2C44055D-54E3-5FA9-8F1D-08E5F0E143D2

##### Notes

**Figure 18.**
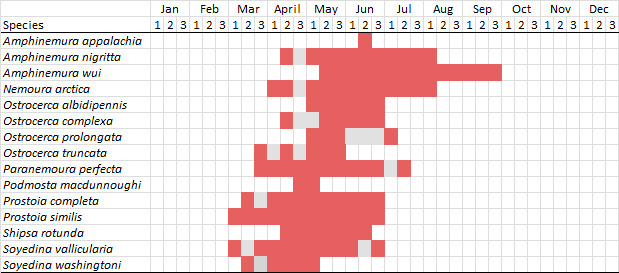
Adult flight period for 15 Nemouridae species in New York State. Red fill indicates positive adult collections while gray shaded areas indicate adults are likely present but not reported.

**Figure 19.**
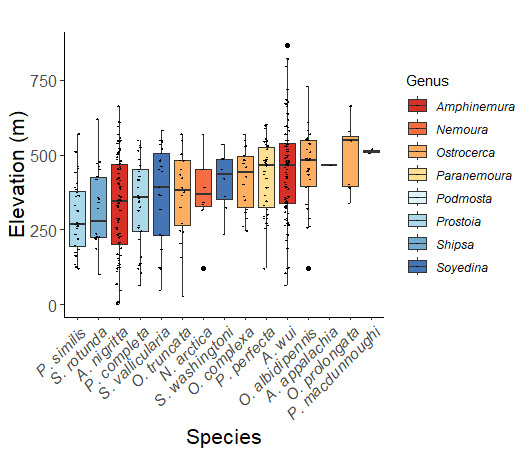
Elevation box plot for 15 Nemouridae species in New York State. Boxes indicate interquartile range, horizontal line in box represents median elevation, and outliers are depicted with large circles.

Nemouridae are commonly referred to as Forestflies (Stark et al. 1998, Stark et al. 2012). Eight genera and 15 species of Nemouridae occur in New York. Although there is a recent review of the Nemourinae of eastern North America (Grubbs and Baumann 2023), identification of the subfamily Amphinemurinae must rely on older literature, namely Ricker (1952), Hitchcock (1974), and Baumann (1996b) to correctly identify *Amphinemura* Ris, 1902 adults to species. Baumann (1975) conducted a global revision of the genera of this family. Adults of this family have been reported from early May to late September (Fig. 18). Nemouridae in New York occupy a wide range of elevations from 2-866 m asl (Fig. 8, Fig. 19). *Amphinemurawui* and *A.nigritta* exhibit the widest range of reported elevations in the state (Fig. 19). In contrast, the species with the narrowest reported elevation ranges are *Amphinemuraappalachia* and *Podmostamacdunnoughi*, presumably due to low limited records of these two species (Fig. 19).

#### Amphinemura appalachia

Baumann, 1996

FC9AAA77-8CE9-5D60-9933-B85008785FDD

##### Notes

*Amphinemuraappalachia* is currently referred to as the Appalachian Forestfly (Stark et al. 2012). The current known distribution of this species extends from Pennsylvania south to Georgia (Baumann 1996b, DeWalt et al. 2024). Baumann (1996b) indicated that this species is less commonly collected than its closely related and later emerging sister species *A.wui* (Claassen, 1936). This single record in New York from Level IV Ecoregion Adirondack High Peaks (58z) (Fig. 17a) represents a northward range extension for this species and a new state record. The New York specimen was collected in mid-June (Fig. 18) from a small headwater stream at 466 m asl (Fig. 19).

#### Amphinemura nigritta

(Provancher, 1876)

80178288-9871-5FF3-8A25-F4B72779DFE8

##### Notes

This species is commonly referred to as the Little Black Forestfly (Stark et al. 2012). The distribution of *A.nigritta* extends from mainland Labrador and the Canadian Maritime Provinces west to Michigan and southward to Alabama and Mississippi, with western extensions to Missouri and Arkansas (DeWalt et al. 2024). Harper (1973a) reported a univoltine life cycle for *A.nigritta* in an intermittent stream in southern Ontario, with emergence dates ranging from mid-May to early July. In New York, adults have been collected from mid-April to early August (Fig. 18). This species was collected commonly from coastal streams on Long Island and smaller streams and rivers throughout the state (Fig. 17c), occupying a wide range of elevations from 2-663 m asl (Fig. 19). Larvae were often found in submerged leaf packs and other in-stream organic material.

#### Amphinemura wui

(Claassen, 1936)

857F13D6-6988-5C57-BE0C-458B569EA839

##### Notes

*Amphinemurawui* is commonly known as the Spiked Forestfly (Stark et al. 2012). The distribution of this common Appalachian species extends from Quebec and the Canadian Maritime Provinces south to Georgia and Alabama (DeWalt et al. 2024). Previous studies in Quebec have suggested a complex univoltine life cycle for this species with two larval cohorts, indicated by an extended emergence period of the adults (Harper and Pilon 1970, Harper 1990b). In New York, this species has been commonly reported from eastern portions of the state including Level III Ecoregions Northeastern Highlands (58), Northeastern Coastal Lowlands (59), Northern Allegheny Plateau (60), and Eastern Great Lakes Lowlands (83) (Fig. 17d). Collection dates of this species from New York also indicate an extended emergence period, with adult collections from mid-May through late September (Fig. 18). This species occupies a wide range of elevations from 65-866 m asl (Fig. 19).

#### Nemoura arctica

Esben-Petersen, 1910

082F79CF-7797-5A7F-BF0F-A5C4B400D02E

##### Notes

This species is commonly referred to as the Arctic Forestfly (Stark et al. 2012). Grubbs et al. (2018) provided evidence with scanning electron microscopy that *N.arctica* is a circumpolar, northern Holarctic species. This species was previously recognized as *N.trispinosa* Claassen, 1923 in eastern North America. The North American range of this species is extensive, reported from Alaska, east across all Canadian provinces, and in the USA from Wyoming east across the Laurentian Great Lakes region to New England states (Grubbs et al. 2018, DeWalt et al. 2024). Harper (1973a) found that larvae of *N.arctica* (as *N.trispinosa*) displayed a slow univoltine life cycle in a southern Ontario stream, with peak emergence occurring in mid-June. New York records are available from Level IV Ecoregions Eastern Adirondack Foothills (58ac), Central Adirondacks (58ad), Adirondack High Peaks (58z), Finger Lakes Uplands and Gorges (60d), and Champlain Lowlands (83b) (Fig. 17e). Adult collection dates range from early April to early August (Fig. 18). Reported elevations for this species in the state range from 119-469 m asl (Fig. 19).

#### Ostrocerca albidipennis

(Walker, 1852)

276C3AF8-9904-5E67-BA6A-2869BBD00183

##### Notes

This species is commonly referred to as the Whitetailed Forestfly (Stark et al. 2012). The distribution of *O.albidipennis* extends from Nova Scotia west to Michigan and southwest along the Appalachian Mountains to Ohio, West Virginia, and Virginia (DeWalt et al. 2024, Grubbs and Baumann 2023). Mackay (1969) reported a fast univoltine life cycle from southern Quebec and the possibility of a summer-autumn egg diapause, with adult emergence during May and June and larval recruitment beginning in early December. This species has been previously reported from smaller streams and spring-fed seeps many of which are dry during the summer months (Harper and Hynes 1971b). New York records are mainly from Level III Ecoregions Northeast Highlands (58), Northern Allegheny Plateau (60), and Eastern Great Lakes Lowlands (83) (Fig. 17f) with adult collection dates ranging from early May to late June (Fig. 18). Elevations in New York range from 119-729 m asl (Fig. 19).

#### Ostrocerca complexa

(Claassen, 1937)

BAA3F9E3-9A2B-5ACB-AE7F-A1D2F16DE071

##### Notes

*Ostrocercacomplexa* is commonly referred to as the Notched Forestfly (Stark et al. 2012). This species ranges from Quebec and the Canadian Maritime Provinces south along the Appalachian Mountains to Virginia and West Virginia (DeWalt et al. 2024, Grubbs and Baumann 2023). In New York, adult collection dates range from mid-April to late June (Fig. 18). This species has been collected from elevations spanning 247-569 m asl (Fig. 19) from small spring-fed streams and seeps mainly in Level III Ecoregions Northeastern Highlands (58) and Northern Allegheny Plateau (60) (Fig. 20a).

#### Ostrocerca prolongata

(Claassen, 1923)

628E3A1E-FDBF-5CEE-B49A-BCCFD4DA6DC4

##### Notes

This is an infrequently reported species commonly known as the Bent Forestfly (Stark et al. 2012). Its current known range extends from Quebec and New Brunswick south along the Appalachian Mountains to Virginia and West Virginia (DeWalt et al. 2024, Grubbs and Baumann 2023, Young et al. 1989). Harper (1990a) studied the life cycle of this species in a small, intermittent Laurentian stream and reported emergence of adults from May to June and suggested a diapause of the eggs during the summer. In New York, adults were collected from early May through early July (Fig. 18). This species was collected from smaller streams and spring-fed seeps at elevations ranging from 337-633 m asl (Fig. 19) from scattered localities in the Level IV Ecoregions Acid Sensitive Adirondacks (58aa), Northern and Western Adirondack Foothills (58ab), Eastern Adirondack Foothills (58ac), Central Adirondacks (58ad), and Adirondack High Peaks (58z) (Fig. 20b).

#### Ostrocerca truncata

(Claassen, 1923)

439012E4-E319-58E9-A2EE-663603B8E25F

##### Notes

*Ostrocercatruncata* is commonly referred to as the Truncate Forestfly (Stark et al. 2012). The distribution of this species extends from Maine west to Ontario and south along the Appalachian Mountains to Alabama, with a westward extension to Kentucky and Indiana (DeWalt et al. 2024, Grubbs and Baumann 2023). Harper et al. (1991b) and Grubbs et al. (2005) both reported a fast univoltine life cycle with a summer egg diapause for *O.truncata*. In New York, adults of this species have been collected from late March through mid-May (Fig. 18). This species occupies a wide range of elevations (26-570 m asl; Fig. 19) and occurs in smaller streams and springs in Level III Ecoregions Northeast Highlands (58), Northern Allegheny Plateau (60), and Eastern Great Lakes Lowlands (83) (Fig. 20c).

#### Paranemoura perfecta

(Walker, 1852)

F546E3E9-A164-5D23-8BE8-F0AE1F91CF01

##### Notes

This species is commonly known as the Spotted Forestfly (Stark et al. 2012). The distribution of *P.perfecta* extends from the Canadian Maritime Provinces west to Michigan and southwest along the Appalachian Mountains to North Carolina and Tennessee (DeWalt et al. 2024, Grubbs and Baumann 2023). No detailed studies have been conducted on the biology or life history attributes of this species. In Canada and northern New England states, the range *P.perfecta* and *P.claasseni* Baumann, 1996 overlap, and in some instances both species have been found in the same stream (Baumann 1996a). In New York, adults of this species were collected early April through mid-July (Fig. 18) from small to medium sized streams at elevations ranging from 119-602 m asl (Fig. 19). This species was commonly collected in Level III Ecoregion Northeastern Highlands (58), with other sporadic reports in the Eastern Great Lakes Lowlands (83) and North Central Appalachians (62) (Fig. 20d). Because a historical record for *P.claasseni* occurs from nearby New Hampshire (Baumann 1996a), this species may eventually be collected in New York.

#### Podmosta macdunnoughi

(Ricker, 1947)

97A267E6-71B4-5644-9090-BDD67BA4F29A

##### Notes

*Podmostamacdunnoughi* is commonly referred to as the Maritime Forestfly (Stark et al. 2012). This species has been reported from Newfoundland-Labrador and Quebec southwest to New York and with an adjunct distribution in Minnesota (DeWalt et al. 2024, Grubbs and Baumann 2023). Harper et al. (1993) found that *P.macdunnoughi* displayed a univoltine-fast life cycle in a Laurentian stream and suggested a summer egg diapause. Our limited collections of this species in New York have indicated adults are active from late April to early May (Fig. 18). Reported elevations in New York range from 505-520 m asl (Fig. 19) from only two sites in Level IV Ecoregion Central Adirondacks (58ad) (Fig. 20e), which is apparently at the southern limits of its range, since this species becomes increasingly more common further north in northeastern Canada (Harper 1990b, Kondratieff and Baumann 1994).

#### Prostoia completa

(Walker, 1852)

B61F0523-D338-5D50-B3D7-EA3CE7F2F4B9

##### Notes

This species is commonly known as the Ozark Forestfly (Stark et al. 2012). The distribution of this common, widespread species extends from eastern Canada west and southwest across much of the eastern USA (DeWalt et al. 2024, Grubbs and Baumann 2023). However, populations from southern Illinois west to Oklahoma now refer to *P.ozarkensis* Baumann and Grubbs, 2014 (Grubbs et al. 2014). Several studies have been conducted on the life history and ecology of this species, all documenting univoltine-fast life cycles for larvae and a summer-autumn egg diapause (Harper 1973a, Ernst and Stewart 1985, Harper et al. 1991b). In New York, adults of *P.completa* were collected from mid-March through late June (Fig. 18). This species was recorded from a wide range of elevations (63-548 m asl; Fig. 19), and appears to be common and abundant with numerous records from Level III Ecoregions Northeastern Highlands (58), Northeastern Coastal Zone (59), and Eastern Great Lakes Lowlands (60) (Fig. 20f).

#### Prostoia similis

(Hagen, 1861)

E185CF2E-0699-5DDC-BFBD-FAB82D21656E

##### Notes

*Prostoiasimilis* is commonly referred to as the Longhorn Forestfly (Stark et al. 2012). This widespread species is known from Ontario and Quebec, south to South Carolina, and west to Missouri (DeWalt et al. 2024, Grubbs and Baumann 2023). Krueger and Cook (1981) studied *P.similis* in central Minnesota and found that larvae displayed a univoltine-fast life cycle similar to previous studies of *P.completa*. Hitchcock (1974) reported collections of this species from Connecticut streams during April. In New York, adults of this species were collected from early March through late June (Fig. 18), from elevations ranging from 119-569 m asl (Fig. 19) and from a wide range of stream sizes across the state (Fig. 21a).

#### Shipsa rotunda

(Claassen, 1923)

A81619C1-D25D-572F-90D3-07CBA4CA3765

##### Notes

*Shipsarotunda* is commonly known as the Intrepid Forestfly (Stark et al. 2012). This species has a very broad distribution extending from mainland Labrador and New Brunswick west through Alaska, in parallel manner from the New England states west through the Laurentian Great Lakes region, and from Maryland south to Georgia and west to Arkansas (Grubbs and Baumann 2021, DeWalt et al. 2024, Grubbs and Baumann 2023). Harper (1973a) examined the life history of *S.rotunda* in Ontario and found that larvae exhibited a univoltine-fast life cycle. Emergence began in May and first instar larvae were present in November, growing steadily until emergence the following spring. Barton (1980) found a similar fast univoltine life cycle for this species in Alberta, except larvae experienced a slowed growth period during the winter months. Adult collection dates from this study range from mid-April through mid-June (Fig. 18). In New York, *S.rotunda* was collected from elevations ranging from 101-621 m asl (Fig. 19) from larger streams and rivers in Level III Ecoregions Northestern Highlands (58) and Eastern Great Lakes Lowlands (83) (Fig. 21b). Nymphs were commonly encountered in leaf packs including those found in seasonally inundated floodplain habitats.

#### Soyedina vallicularia

(Wu, 1923)

30DC8310-5EB4-5261-879E-64A90F4F09AB

##### Notes

This species is commonly referred to as the Valley foestfly (Stark et al. 2012). *Soyedinavallicualaria* is known in Canada from mainland Labrador and Nova Scotia west to Ontario and in the USA from Maine west to Wisconsin and Iowa and southwest to North Carolina and Tennessee (DeWalt et al. 2024, Grubbs and Baumann 2023). Mackay (1969) and Harper (1973a) documented a univoltine-slow life cycle for *S.vallicularia* from streams from Quebec and southern Ontario, respectively. Adults were present in May and larval recruitment began in June indicating a direct hatching of the eggs. In New York, adults of this species were active from early March through late June (Fig. 18). *Soyedinavallicularia* was recorded from a wide range of elevations ranging from 47-582 m asl (Fig. 19). Although this species was collected frequently in the eastern half of the state (Fig. 21c), we anticipate that it is more common in western New York.

#### Soyedina washingtoni

(Claassen, 1923)

5F4DF3C9-B7E9-58E7-BE38-4D64EC07556C

##### Notes

*Soyedinawashingtoni* is commonly known as the Vernal Forestfly (Stark et al. 2012). This species has been reported from the Canadian Maritime Provinces southwest to West Virginia (DeWalt et al. 2024, Grubbs and Baumann 2023). No life history or ecological information is available regarding this species. Adult collection dates for *S.washingtoni* in the state range from mid-March through early May (Fig. 18). In New York, this species was collected at higher elevations (234-536 m asl; Fig. 19) from springs and seeps in Level IV Ecoregions Taconic Mountains (58a), Taconic Foothills (58x), Catskill High Peaks (58y), Adirondack High Peaks (58z), 58ac Eastern Adirondack Foothills (58ac), and Central Adirondacks (58ad) (Fig. 21d).

#### Taeniopterygidae

Klapálek, 1905

CE2F678C-44E6-5F41-ADFD-61BA62FE7EBA

##### Notes

Taeniopterygidae are commonly referred to as Willowflies (Stark et al. 1998, Stark et al. 2012). Similar to Capniidae, adults of this family also emerge during winter and early spring months and together are known historically as "winter stoneflies" (Stewart and Stark 2002). Six genera and 11 species of Taeniopterygdae occur in New York. Stewart (2000) provided the most recent review of the adults of eastern North American species of this family. Larvae of eastern North American species appear to undergo a summer diapause similar to many capniids (Stewart and Stark 2002). Verdone et al. (2025) recently published an updated key to genera of adults and larvae for eastern North American taeniopterygids and a key to males, females and larvae of species of *Oemopteryx*, including two new species, from North America.

**Figure 22.**
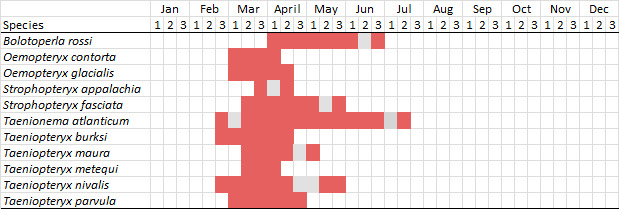
Adult flight period for 11 Taeniopterygidae species in New York State. Red fill indicates positive adult collections while gray shaded areas indicate adults are likely present but not reported.

**Figure 23.**
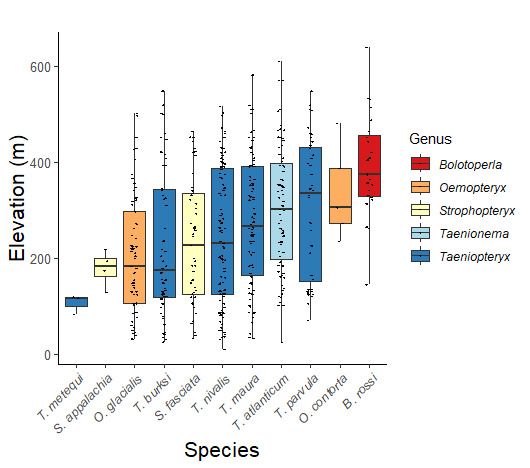
Elevation box plot for 11 Taeniopterygidae species in New York State. Boxes indicate interquartile range, horizontal line in box represents median elevation, and outlliers are depicted with large circles.

Published adult collection dates for this family range from late February to mid-July (Fig. 22). Taeniopterygidae in New York have been reported from elevations ranging from 9-639 m asl (Fig. 23). Nearly all of the species reported from New York are present at lower elevations below 100 m asl, with the exception of *Oemopteryxcontorta* and *Bolotoperlarossi*. *Taeniopteryxmetequi* was collected from both the lowest and most narrow range of reported elevations in the state.

#### Bolotoperla rossi

(Frison, 1942)

AAC541DA-E0EC-591C-83DB-DE194D1BB3E7

##### Notes

*Bolotoperlarossi* is commonly referred to as the Smoky Willowfly (Stark et al. 2012). This is an Appalachian-distributed species reported from Quebec and Maine south to South Carolina and Tennessee (Stewart 2000, Stark et al. 2016, DeWalt et al. 2024.) Prior reports of this species from New York by Stewart (2000) are based on a misinterpretation of the holotype locality “Near Woodstock, Bog Brook, NH”. Myers et al. (2011) reported this species from the Adirondack Park but without locality data. Larvae of this species were previously documented from 2nd- to 4th-order Appalachian streams with peak emergence occurring in March and early April (Kirchner and Harper 1983). In New York, this species appears to be common and has been reported from larger streams and rivers in Level IV Ecoregions Adirondack High Peaks (58z), Acid Sensitive Adirondacks (58aa), Eastern Adirondack Foothills (58ac), Central Adirondacks (58ad), and Champlain Lowlands (83b) (Fig. 21e). Adult collection dates ranged from early April to the end of June (Fig. 22), at elevations ranging from 144-639 m asl (Fig. 23).

#### Oemopteryx contorta

(Needham & Claassen, 1925)

E711CF72-009B-546A-95AA-BBA382037521

##### Notes

This species is commonly referred to as the Dark Willowfly (Stark et al. 2012). *Oemopteryxcontorta* is distributed along the Appalachian Mountains from Maine southwest to North Carolina and Tennessee (Stewart 2000, DeWalt et al. 2024, Verdone et al. 2025). Nelson (1982) studied the life history of this species in Tennessee and found that larvae exhibited a univoltine-slow life cycle. Larvae were first collected in October, and adult emergence occurred in April. In New York, this species is uncommon and apparently restricted to smaller streams with records available from Level IV Ecoregions Glaciated Reading Prong/Hudson Highlands (58i), Taconic Foothills (58x), Adirondack High Peaks (58z), Central Adirondacks (58ad), and Catskills Transition (60c) (Fig. 21f). Adults have been collected from early March through late April (Fig. 22) from small, cold streams at elevations ranging from 234-480 m asl (Fig. 23).

#### Oemopteryx glacialis

(Barnston, 1848)

DD8FDE5A-E2A7-57A7-8CEB-8422EF7725AF

##### Notes

*Oemopteryxglacialis* is commonly known as the Canadian Willowfly (Stark et al. 2012). This species is common in large rivers, ranging from Quebec west though the Laurentian Great Lakes region to Minnesota, south to New York and Connecticut, and with a disjunct distribution southward to West Virginia (Stewart 2000, DeWalt et al. 2024, Verdone et al. 2025). In Quebec, this species exhibits a univoltine-fast life cycle with direct hatching of eggs, larval diapause during summer and early autumn months, and emergence of adults occurring in April (Harper et al. 1991a). In New York, adults are active from early March through mid-April (Fig. 22). Reported elevations for this species in the state range from 30-502 m asl (Fig. 23) with a widespread distribution in larger rivers north of the Mohawk River Valley (Fig. 24a).

#### Strophopteryx appalachia

Ricker & Ross, 1975

A832D5EA-2F68-5135-A722-BCE376312398

##### Notes

*Strophopteryxappalachia* is commonly referred to as the Appalachian Willowfly (Stark et al. 2012). This is an Appalachian-distributed species known from New York southwest to Tennessee and Georgia (Stewart 2000, DeWalt et al. 2024, Verdone et al. 2025). Larvae were described by Earle and Stewart (2008). In New York, adults of this species have been collected from late March through mid-April (Fig. 22). *Strophopteryxappalachia* has been reported from elevations ranging from 128-218 m asl (Fig. 23) from only four locations in Level IV Ecoregions Taconic Foothills (58x), Hudson Valley (59i), and Northern Glaciated Limestone Ridges, Valleys, and Terraces (67l) (Fig. 24b). Further collection efforts in the southern tier of the state may yield additional records.

#### Strophopteryx fasciata

(Burmeister, 1839)

876ED9D0-E762-56A0-964B-B462371FEE85

##### Notes

This species is commonly referred to as the Mottled Willowfly (Stark et al. 2012). *Strophopteryxfasciata* is a widespread and common late winter to late spring emerging species (Stewart 2000, DeWalt et al. 2024). The North American distribution of this species spans from Manitoba and North Dakota to the eastern coast, south to the Ouachita Mountains of Arkansas and east to Georgia (DeWalt et al. 2024, Stewart 2000, Verdone et al. 2017). Harper and Hynes (1971c) reported *S.fasciata* from large streams and rivers in southern Quebec and Ontario. Previous studies of this species have indicated a fast univoltine life cycle with larvae undergoing a summer autumn diapause similar to *Oemopteryx* (Harper and Hynes 1972, Harper et al. 1991a). In New York, adults of this species have been collected from mid-March through late May (Fig. 22), from elevations ranging from 33-464 m asl (Fig. 23) and are widely distributed across the state (Fig. 24c).

#### Taenionema atlanticum

Ricker & Ross, 1975

D63B053D-99AF-5C50-9669-91AFAAC47AAD

##### Notes

This species is commonly referred to as the Atlantic Willowfly (Stark et al. 2012), and is known from Quebec and Newfoundland-Labrador southwestward mainly through the Appalachian Mountains to Tennessee and South Carolina (Stewart 2000, DeWalt et al. 2024). Harper et al. (1991a) reported a univoltine life cycle from a small Laurentian Highlands stream in Quebec. In New York, adults of *T.atlanticum* have been collected from late February to mid-July (Fig. 22), from elevations ranging from 25-609 m asl (Fig. 23). This species is widely distributed across the state and is commonly collected from small streams from the Appalachian Plateau east to the Adirondacks and surrounding lowlands, and south to the Lower Hudson Valley near the northern edge of New York City (Fig. 24d).

#### Taeniopteryx burksi

Ricker & Ross, 1968

CF32DA19-7CFA-592B-AC8A-53629F119944

##### Notes

*Taeniopteryxburksi* is commonly known as the Eastern Willowfly (Stark et al. 2012). This common species is distributed extensively across eastern North America, with records as far west as Colorado, Oklahoma, and Texas (Stewart 2000, DeWalt et al. 2024). Previous studies of this species indicate a univoltine life cycle with a long period of larval diapause (Harper and Magnin 1969, Harper and Hynes 1972). In New York, adults of *T.burksi* have been collected from early March to late May (Fig. 22) at elevations ranging from 25-548 m asl (Fig. 23) and from larger streams and rivers across the state (Fig. 24e).

#### Taeniopteryx maura

(Pictet, 1841)

2BABC8CE-95EB-5D3E-B51F-6C429D6F4321

##### Notes

*Taeniopteryxmaura* is commonly referred to as the Spinyleg Willowfly (Stark et al. 2012). The distribution of this species extends from southeastern Canada southwest to Tennessee and Mississippi, and further west to Oklahoma and Texas (Stewart 2000, DeWalt et al. 2024). In southern Quebec, this species displays a univoltine-fast life cycle with a diapause period during the summer and early autumn (Harper et al. 1991b). Adults of this species are commonly encountered from mid-March to early May (Fig. 22) from a wide range of elevations (31-582 m asl; Fig. 23) in both small streams and large rivers across the state (Fig. 24f).

#### Taeniopteryx metequi

Ricker & Ross, 1968

7A9C56CE-F373-588C-9F74-7590D500E060

##### Notes

This species is commonly known as the Shortwing Willowfly (Stark et al. 2012). *Taeniopteryxmetequi* is distributed from Ontario, south to Alabama and Virginia, and southwest to Kansas and Oklahoma (Stewart 2000, DeWalt et al. 2024). No studies pertaining to the life history or ecology of this species are available. Adults were collected in New York from mid-March through early April (Fig. 22). This species occupies low elevation (83-119 m asl; Fig. 23) and low gradient streams, with records available from Level IV Ecoregions Champlain Lowlands (83b) and Ontario Lowlands (83c) (Fig. 25a). Further collections in western New York may provide additional distributional records.

#### Taeniopteryx nivalis

(Fitch, 1847)

DD1AAF63-3FEC-54CE-9022-196D845D05D6

##### Notes

*Taeniopteryxnivalis* is commonly referred to as the Boreal Willowfly (Stark et al. 2012). This species has an unusual distribution in North America. In Canada, *T.nivalis* has been reported from mainland Newfoundland and Nova Scotia west to Alberta. In the USA, records extend from Maine west across the Laurentian Great Lakes region to Minnesota and Iowa and southwestward to Maryland and West Virginia. There is also a disjunct band from Idaho and Utah west to Oregon and Washington (Stewart 2000, DeWalt et al. 2024). Harper and Hynes 1972, Knight et al. (1976), and Sephton and Hynes (1984) have each reported univoltine-fast life cycles for *T.nivalis* nearly identical to that of *T.maura*. In New York, adults of *T.nivalis* are present from late February to the end of May (Fig. 22) . This species is common in large rivers and streams ranging in elevation from 9-515 m asl (Fig. 23) and is widespread across the state (Fig. 25b).

#### Taeniopteryx parvula

Banks, 1918

443F7F78-4BF1-51D2-8210-F58D34FE5CDF

##### Notes

*Taeniopteryxparvula* is commonly known as the Hooked Willowfly (Stark et al. 2012). Similar to *T.nivalis*, this species also has an unusual distribution in North America. In Canada, records exist from Nova Scotia and Quebec west to Manitoba and Alberta. In the USA, *T.parvula* has been reported from most states east of the Mississippi River plus Minnesota, Missouri, and Arkansas. There is also a disjunct band from Wyoming south to New Mexico (Stewart 2000, DeWalt et al. 2024). This species was commonly collected from larger streams and rivers in the Adirondacks (Myers et al. 2011). Harper and Harper (1983) similarly reported *T.parvula* from rivers in Quebec, and reported a univoltine-fast life cycle for *T.parvula*, with seasonal diapause similar to that of *T.maura* and *T.nivalis*. Adults have been collected in New York between early March and late April (Fig. 22) from large streams and rivers at elevations ranging from 69-548 m asl (Fig. 23) and are broadly distributed across much of the state (Fig. 25c).

#### Chloroperlidae

Okamoto, 1912

3DD53D9F-EF0F-5ABC-9800-BB832694CB90

##### Notes

**Figure 26.**
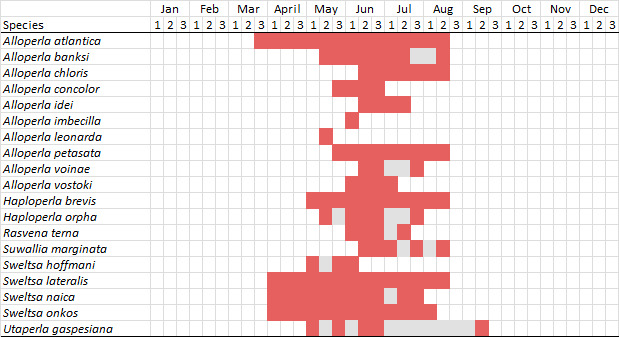
Adult flight period for 19 Chloroperlidae species in New York State. Red fill indicates positive adult collections while gray shaded areas indicate adults are likely present but not reported.

**Figure 27.**
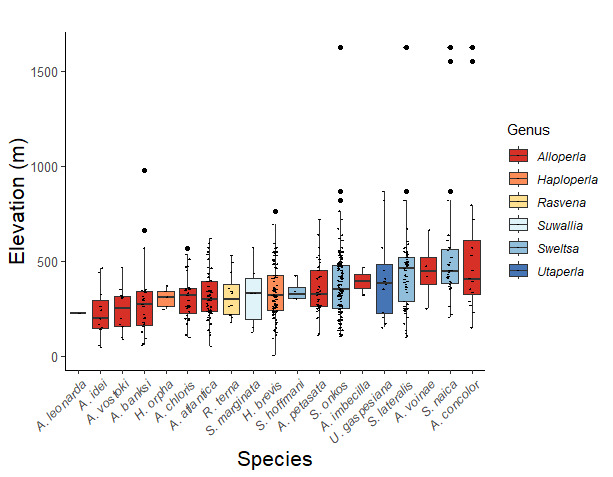
Elevation box plot for 19 Chloroperlidae species in New York State. Boxes indicate interquartile range, horizontal line in box represents median elevation, and outlliers are depicted with large circles.

Chloroperlidae are commonly referred to as Sallflies (Stark et al. 1998, Stark et al. 2012). Surdick (2004) provided the most recent review of the eastern North American species of this family. Adults are easily recognized in the field, with coloration ranging from a subtle, pale yellow to bright green. In New York, this family is comprised of 19 species and six genera, several of which are rare or infrequently collected. Adult collection dates range from early April through mid-September (Fig. 26) across a very broad elevation range (3-1623 m asl; Fig. 27). Species occupying lower elevations include *Alloperlaleonarda*, *A.idei*, *A.vostoki*, and *A.banksi*. The widest range of reported elevations was recorded for common species, including *Haploperlabrevis*, *Sweltsalateralis*, *S.onkos*, *S.naica*, and *A.concolor*. Narrow ranges of elevation are reported for *A.leonarda*, *H.orpha*, *S.hoffmani*, *and A. imbecilla*.

#### Alloperla atlantica

Baumann, 1974

A0459DD7-02DA-5F0F-A6D5-BE365B963EF2

##### Notes

*Alloperlaatlantica* is commonly referred to as the Atlantic Sallfly (Stark et al. 2012). The distribution of this species extends from the Canadian Maritime Provinces and Quebec west through the northern Laurentian Great Lakes region to Minnesota and southwest to Tennessee and Alabama (Surdick 2004, DeWalt et al. 2024). Harper and Pilon (1970) reported a short synchronous emergence from late May to mid-June in Quebec. This species was commonly collected in the eastern half of the state (Fig. 25d). Further surveys in western New York should yield additional records. Adults in New York had an extended flight period with adults present from late May through early August (Fig. 26) at elevations from 49-620 m asl (Fig. 27).

#### Alloperla banksi

Frison, 1942

ACAC1EB0-0467-56F4-9C56-E784D9871C6A

##### Notes

This species is commonly known as the Tufted Sallfly (Stark et al. 2012). *Alloperlabanksi* is known from Michigan, New York, and southeastern Canada, with a disjunct distribution in Virginia (Surdick 2004, DeWalt et al. 2024). As in most other members of the genus, larvae of *A.banksi* are currently unknown and there is little published life history information beyond adult phenology (Stewart and Stark 2002). In New York, this species was collected frequently from larger streams and rivers across much of the eastern portion of the state (Fig. 25e). Adults have been reported from mid-May through early August (Fig. 26) from elevations ranging from 57-978 m asl (Fig. 27).

#### Alloperla chloris

Frison, 1934

ECBAFB1F-7F9A-5C02-8BC5-9C360A177F72

##### Notes

*Alloperlachloris* is commonly referred to as the Triangular Sallfly (Stark et al. 2012). This species has an Appalachian distribution from southeastern Canada south to Alabama and Georgia (Surdick 2004, DeWalt et al. 2024). Fishbeck (1987) and DeWalt et al. (2016) reported this species from small streams in the glaciated northeastern corner of Ohio where adults were taken from May to August. Most records of this species are from eastern areas of New York (Fig. 25f), but it is likely more abundant and widespread than current collections indicate. In New York, adults have been collected between early June and mid-August (Fig. 26) from upland streams and rivers at elevations ranging from 97-568 m asl (Fig. 27).

#### Alloperla concolor

Ricker, 1936

646BA1BC-559C-5966-916D-EA60C4570995

##### Notes

*Alloperlaconcolor* is commonly known as the Duckhead Sallfly (Stark et al. 2012). The distribution of this species extends in Canada from Labrador west to Ontario and southeast along the Appalachian Mountains to Virginia and West Virginia (Surdick 2004, DeWalt et al. 2024). Previous reports of *A.neglecta* Frison, 1935 from New York pertain to *A.concolor* (Kondratieff and Kirchner 1993). Harper and Pilon (1970) reported emergence of adults from mid-May to early June in a Quebec stream. Adults have been collected frequently from New York between early June and mid-August (Fig. 26) from a broad range of elevations from 148-1623 m asl (Fig. 27). This species is widespread in Level III Ecoregions Northeastern Highlands (58) and Northern Allegheny Plateau (60) (Fig. 28a) and is likely more common in western areas of the state.

#### Alloperla idei

(Ricker, 1935)

B8D8CE4F-C452-5E6A-B106-352D6E27A230

##### Notes

This species is commonly referred to as the Vernal Sallfly (Stark et al. 2012). *Alloperlaidei* has been reported from New Brunswick west to Ontario and patchily southwest to Alabama and Georgia (Surdick 2004, DeWalt et al. 2024). DeWalt et al. (2016) reported adults from three small streams in May in the southeastern Ohio portion of the Appalachian Plateau. Harper and Pilon (1970) documented peak emergence of this species in late May and early June from a Quebec stream. In New York, adults were collected from mid-June to mid-July (Fig. 26) at elevations ranging from 43-563 m asl (Fig. 27). This species can be common in Level IV Ecoregions Acid Sensitive Adirondacks (58aa), Eastern Adirondack Foothills (58ac), Central Adirondacks (58ad). Hudson Valley (59i), Catskills Transition (60c), and Ontario Lowlands (83c) (Fig. 28b).

#### Alloperla imbecilla

(Say, 1823)

3233D9C0-649C-5CFF-A79A-633729CC2883

##### Notes

*Alloperlaimbecilla* is commonly known as the Ohio Sallfly (Stark et al. 2012). This species has been reported in Canada from New Brunswick and in the USA from New York southwest to Virginia and Kentucky, primarily with an Ohio River Valley distribution (Baumann 1974, Surdick 2004, DeWalt et al. 2024). Adults have been collected infrequently from the state in early June (Fig. 26) at elevations ranging from 321-467 m asl (Fig. 27) and appears to be restricted to Level IV Ecoregions Glaciated Low Allegheny Plateau (60a) and Finger Lakes Uplands and Gorges in western New York (60d) (Fig. 28c). These records originated from material collected by the late Verne Pechumann at Cornell University in the late 1970's. Further collections in the western portion of the state will likely yield additional records.

#### Alloperla leonarda

Ricker, 1952

62626F5D-53BE-5445-A595-E9FBE3CA6EF3

##### Notes

This species is commonly referred to as the Truncate Sallfly (Stark et al. 2012). *Alloperlaleonarda* is one of the least commonly collected species in eastern North America, with geographic clusters in southeastern Canada, Michigan, and Minnesota, plus a single disjunct locality in southern Missouri (Surdick 2004, Willett and Stark 2009, DeWalt et al. 2024). A single adult male of this species was collected on May 22, 2008 (Fig. 26) at 225 m asl (Fig. 27) from the Black River in Level IV Ecoregion Upper St. Lawrence Valley (83e) (Fig. 28d), representing a new state record for New York.

#### Alloperla petasata

Surdick, 2004

56FB970E-2A93-5BB7-A424-F9F9E0FDF084

##### Notes

*Alloperlapetasata* is commonly referred to as the Woodlands Sallfly (Stark et al. 2012). This is a common species known from Labrador east to Ontario and southwest to Tennessee and Georgia (Surdick 2004, DeWalt et al. 2024). Previously, this species was known as *A.caudata* Frison, 1934 (Surdick 2004), a smaller species of the unglaciated states west of New York. Fishbeck (1987) reported peak emergence in mid-June from Gray’s Run in Northeastern Ohio. In New York, adults of *A.petasata* have been collected frequently from late May to mid-August (Fig. 26), often in association with *A.atlantica* and *A.chloris*. Documented occurrences of *A.petasata* from the study area range in elevation from 111-720 m asl (Fig. 27) from numerous streams and rivers in Level III Ecoregions Northeast Highlands (58), Northern Allegheny Plateau (60), and Eastern Great Lakes Lowlands (83) (Fig. 28e).

#### Alloperla voinae

Ricker, 1947

21680E9D-06C4-524D-B9F4-1DB371B3AC61

##### Notes

*Alloperlavoinae* is commonly known as the Lawrence Sallfly (Stark et al. 2012). This species is considered rare in Canada and the USA and is subsequently listed as vulnerable to extirpation or extinction throughout its range (NatureServe 2024). Records are available for Canada from Nova Scotia and Quebec and in the USA only from Maine southeast to Massachusetts and New York (Surdick 2004, DeWalt et al. 2024). Adults have been collected in New York between mid-June and late July (Fig. 26) from cold, small to medium sized streams at elevations ranging from 248-663 m asl (Fig. 27) in Level IV Ecoregions Adirondack High Peaks (58z), Eastern Adirondack Foothills (58ac), and Central Adirondacks (58ad) (Fig. 28f).

#### Alloperla vostoki

Ricker, 1947

C402375E-9C8C-5CC1-981B-846C8CA15E9E

##### Notes

This species is commonly referred to as the Scotia Sallfly (Stark et al. 2012). Similar to *A.voinae*, this rare species is also considered vulnerable to extirpation or extinction (NatureServe 2024). *Alloperlavostoki* has been reported infrequently in Canada from Nova Scotia and New Brunswick and in the USA from Maine, New York, and Pennsylvania (Surdick 2004, DeWalt et al. 2024). This species was collected most efficiently with beating sheets and UV light trapping methods. Adults of this species have been collected in New York from early June through early July (Fig. 26) at elevations ranging from 88-466 m asl (Fig. 27) from sporadic locations in Level IV Ecoregions Catskill High Peaks (58y), Tug Hill Transition (58af), Hudson Valley (59i), Glaciated Low Allegheny Plateau (60a), Finger Lakes Uplands and Gorges (60d), Ontario Lowlands (83c), and Mohawk Valley (83f) (Fig. 29a). This species was encountered in small to medium sized streams and rivers with underlying shale formations.

#### Haploperla brevis

(Banks, 1895)

86E8A5E3-F858-5B84-8949-11A893B77540

##### Notes

*Haploperlabrevis* is commonly known as the Least Sallfly (Stark et al. 2012). This widespread and common species is known from the Canada Atlantic Maritime Provinces west to British Columbia and Nunavut and in the USA from Maine west to Oklahoma and Arkansas, south to Florida, and southwest to Oklahoma (Surdick 2004, DeWalt et al. 2024, Hart et al. 2025). Previous studies on the biology and life history of this species have indicated a two-to-five-month egg diapause and a slow univoltine life cycle in Quebec and Oklahoma, respectively (Harper and Magnin 1969, Ernst and Stewart 1985). In contrast, Barton (1980) indicated a univoltine-slow life cycle for *H.brevis* in Alberta. Adults have been collected in New York from early May to mid-August (Fig. 26) from elevations ranging from 3-763 m asl (Fig. 27), and from a wide range of stream sizes across the state (Fig. 29b).

#### Haploperla orpha

(Frison, 1937)

75D90D86-D36B-5093-A4E9-32B56A679A4C

##### Notes

*Haploperlaorpha* is commonly referred to as the Quadrate Sallfly (Stark et al. 2012). The range of this uncommon species extends in Canada from New Brunswick west to Ontario and in the USA from Maine west across the Laurentian Great Lakes region to North Dakota (Surdick 2004, DeWalt et al. 2024). Scarce biological information exists for this species other than adult phenology and its collection in larger rivers (Surdick 2004). Adults have been collected infrequently in New York between mid-May through late July (Fig. 26) from streams and rivers at elevations ranging from 246-369 m asl (Fig. 27) in Level IV Ecoregions Northern and Western Adirondack Foothills (58ab), Eastern Adirondack Foothills (58ac), and Champlain Lowlands (83b) (Fig. 29c). It is probably more common than is presented here. Use of beating sheets and sweepnets along large rivers in June and July will likely increase the number of known locations.

#### Rasvena terna

(Frison, 1942)

C0CAE281-0795-5694-A4F4-AC36B4FC8ABC

##### Notes

This rare species is commonly referred to as the Vermont Sallfly (Stark et al. 2012), distributed mainly along the Appalachian Mountains from southern Quebec south to north Georgia (Surdick 2004, Grubbs and Singai 2018, DeWalt et al. 2024). Grubbs and Singai (2018) provided scanning electron micrographs of diagnostic adult and larval characteristics together with a discussion of the distribution of this species. No larval biology is known. Adults have been collected in New York from mid-May to late June (Fig. 26) at elevations ranging from 175-529 m asl (Fig. 27) with few records available from Level IV Ecoregions Taconic Mountains (58a), Taconic Foothills (58x), Adirondack High Peaks (58z), Eastern Adirondack Foothills (58ac), Central Adirondacks (58ad), and Champlain Lowlands (83b) (Fig. 29d).

#### Suwallia marginata

(Banks, 1897)

F23AF613-41F3-52D6-AFEB-99BBE57E0EE1

##### Notes

*Suwalliamarginata* is commonly known as the York Sallfly (Stark et al. 2012). This species has been reported in Canada from New Brunswick and mainland Labrador west to Ontario and in the USA from Maine south to Georgia (Alexander and Stewart 1999, Surdick 2004, DeWalt et al. 2024). Surdick (2004) reported adult collections of this species from June to September. Our records from New York indicate a flight period extending from early June to late August (Fig. 26). This species has been recorded from streams ranging from 125-570 m asl (Fig. 27) from Level IV Ecoregions Catskill High Peaks (58y), Northern and Western Adirondack Foothills (58ab), Finger Lakes Uplands and Gorges (60d), and Cattaraugus Hills (60f) (Fig. 29e). This species is likely more common in the state than current collections indicate.

#### Sweltsa hoffmani

Kondratieff & Kirchner, 2009

D9AA19D9-17FF-547B-A3A6-15E24E2C8F2A

##### Notes

This species is commonly referred to as the Plateau Sallfly (Stark et al. 2012), and is known from unglaciated portions of the Alleghany and Cumberland Plateaus from New York southwest to Alabama and west across Ohio (DeWalt et al. 2016) to Indiana (DeWalt and Grubbs 2011, Kondratieff and Kirchner 2009, DeWalt et al. 2024). Fishbeck (1987) reported an early June to mid-July emergence of this species (as *S.onkos*) from Gray’s Run in northeastern Ohio. In New York, adults of this species have been recorded from early May through early June (Fig. 26) at elevations ranging 300-422 m asl (Fig. 27) from small streams in Level IV Ecoregions Glaciated Low Allegheny Plateau (60a), Low Lime Drift Plain (61c), and Finger Lakes Uplands and Gorges (60d) (Fig. 29f).

#### Sweltsa lateralis

(Banks, 1911)

7D613CD0-7E19-5F24-9DD6-AE01AF083D54

##### Notes

*Sweltsalateralis* is commonly known as the Curved Sallfly (Stark et al. 2012). This common and widespread species is distributed in Canada from New Brunswick west to Ontario and in the USA mainly along the Appalachian Mountains from Maine south to Tennessee (Surdick 2004, DeWalt et al. 2024). Previous life history studies of this species have indicated a multivoltine-slow two-year life cycle (Huryn and Wallace 1987.) Fishbeck (1987) reported the adult flight period for *S.lateralis* from early June to mid-July in northeastern Ohio. Adults are present in New York from early May through mid-August (Fig. 26) at elevations ranging from 98-1623 m asl (Fig. 27). This species was widely distributed in small streams and rheocrenes, with distributional records available from upland areas across the state (Fig. 30a).

#### Sweltsa naica

(Provancher, 1876)

8D396FCF-44A0-55B3-A89A-39E95F9B02BB

##### Notes

This species is commonly referred to as the Northeastern Sallfly (Stark et al. 2012). The distribution of *S.naica* extends from Newfoundland-Labrador, Quebec, and the Canadian Maritime Provinces south to Virginia and West Virginia (Surdick 2004, DeWalt et al. 2024). No life history studies have been conducted on *S.naica*. Adults have been collected in New York from May to mid-June (Fig. 26) from small upland streams and rheocrenes at elevations ranging from 204-1623 m asl (Fig. 27). This species was commonly collected from Level IV Ecoregions Upper Montane/Alpine Zone (58j), Catskill High Peaks (58y), Adirondack High Peaks (58z), Acid Sensitive Adirondacks (58aa), Eastern Adirondack Foothills (58ac), and Central Adirondacks (58ad) (Fig. 30b).

#### Sweltsa onkos

(Ricker, 1936)

F848A2AC-527C-52B5-9E92-E41B527A90EA

##### Notes

*Sweltsaonkos* is commonly known as the Ontario Sallfly (Stark et al. 2012). The distribution of this species extends from across southeastern Canada south to North Carolina (Surdick 2004, Kondratieff and Kirchner 2009, DeWalt et al. 2024). Museum specimens and both published and unpublished reports of *S.onkos* from unglaciated sections of several USA states (e.g., Alabama, Kentucky, and Tennessee) likely refer to *S.hoffmani. Sweltsaonkos* is one of the most abundant species in New York. Previous life history studies of *S.onkos* have indicated a slow two-year life cycle with most growth occurring in the second year (Harper 1973b, Harper et al. 1991b). Harper and Pilon (1970) reported the emergence of this species from late May to early July in Quebec. Adults have been collected in New York from mid-May through early August (Fig. 26) from a wide range of elevations from 104-1623 m asl (Fig. 27) and numerous locations across the state (Fig. 30c).

#### Utaperla gaspesiana

Harper and Roy, 1975

084F1605-B78F-560C-AEF1-C7FB58CA77FF

##### Notes

*Utaperlagaspesiana* is commonly referred to as the Gaspe Sallfly (Stark et al. 2012). This is a rare species reported from Quebec south to West Virginia (Surdick 2004, DeWalt et al. 2024, NatureServe 2024). Harper et al. (1991b) examined the life history of *U.gaspesiana* in Quebec, suggesting a multivoltine-slow, two-year life cycle similar to that of *S.onkos*. Adults have been collected in New York from early May to mid-September (Fig. 26) at 149-866 m asl (Fig. 27), but infrequently from coarse, open substrated, medium-sized streams and small rivers from Level IV Ecoregions Catskill High Peaks (58y), Eastern Adirondack Foothills (58ac), Glaciated Low Allegheny Plateau (60a), Catskills Transition (60c), and Champlain Lowlands (83b) (Fig. 30d). They are likely to utilize hyporheic habitats as done by other chloroperlids, especially those in the subfamily Paraperlinae (South et al. 2021).

#### Peltoperlidae

Claassen, 1931

1492C556-6955-51B6-B647-9A0F85E74E01

##### Notes

**Figure 31.**
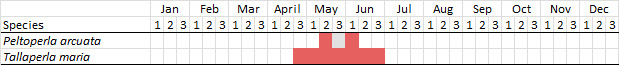
Adult flight period for two Peltoperlidae species in New York State. Red fill indicates positive adult collections while gray shaded areas indicate adults are likely present but not reported.

**Figure 32.**
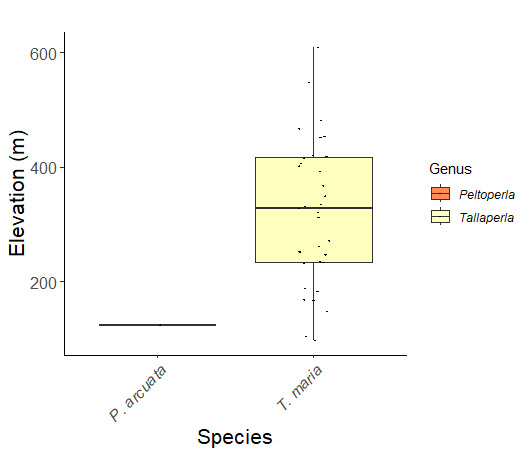
Elevation box plot for two Peltoperlidae species in New York State. Boxes indicate interquartile range, horizontal line in box represents median elevation, and outlliers are depicted with large circles.

Peltoperlidae are commonly referred to as Roachflies (Stark et al. 1998, Stark et al. 2012). Larvae can be easily recognized in the field by their ovoid, roach-like shape (Stewart and Stark 2002, see their fig. 12.1). Only two genera and two species have been reported from the state. Stark (2000) provided the most recent review of the eastern North American species of this family. Adult collection dates range from late April through the end of June (Fig. 31). Peltoperlidae in New York occupy a wide range of elevations at 98-609 m asl (Fig. 32), with *Peltoperlaarcuata* restricted to a single historical collection location at 125 m asl. *Tallaperlamaria* is more common and widespread and is reported from elevations ranging from 98-609 m asl.

#### Peltoperla arcuata

Needham, 1905

B3FBF8F1-A0B8-55C4-A2DB-B4EDB865D64C

##### Notes

This species is commonly referred to as the Appalachian Roachfly (Stark et al. 2012), and is known from Quebec south mainly along the Appalachian Mountains to Tennessee (Stark 2000, DeWalt et al. 2024). DeWalt et al. (2016) found this species to be relatively common in headwater streams of eastern Ohio. Several larval records from southern and central New York were excluded from our study since positive separation of larval *Peltoperla* and *Talloperla* can be problematic, especially in older material. Stark (2017) provided new characters that need to be evaluated with New York material of both genera. Yokum et al. (1995) documented a semivoltine life cycle for a population of *P.arcuata* in West Virginia. Eggs underwent a six-month diapause period followed by 18 months of continuous larval growth, with emergence of adults from late May through July. In contrast, Miller and Kovalak (1979) report a univoltine life cycle for *P.arcuata* in Pennsylvania. The sole report of this species in New York is based on the historical type material collected in June 1891 (Fig. 31) at approximately 125 m asl (Fig. 32) from Level IV Ecoregion Finger Lakes Uplands and Gorges (60d) in the vicinity of Ithaca (Fig. 30e). We suspect that this species is more common than our sampling suggests. Rearing of larvae from lower elevation headwater streams would likely yield several populations.

#### Tallaperla maria

(Needham & Smith, 1916)

80549CF2-5B8B-5997-A7A4-856CF9910497

##### Notes

*Tallaperlamaria* is commonly known as the Common Roachfly (Stark et al. 2012). This common species ranges from Quebec and Maine south along the Appalachian Mountains to Georgia and Alabama (Stark 2000, DeWalt et al. 2024). Life history studies of *T.maria* have indicated a two-year life cycle (Harper et al. 1991a, Grubbs and Cummins 1996). In New York, adult collection dates for *T.maria* range from early April to late June (Fig. 31). This species was collected commonly from elevations ranging from 98-609 m asl (Fig. 32), with numerous records available from small headwater streams and across upland and foothill areas of the state (Fig. 30f).

#### Perlidae

Latreille, 1802

2379F6A6-EA6E-50D2-8799-AD1BB30BF183

##### Notes

**Figure 33.**
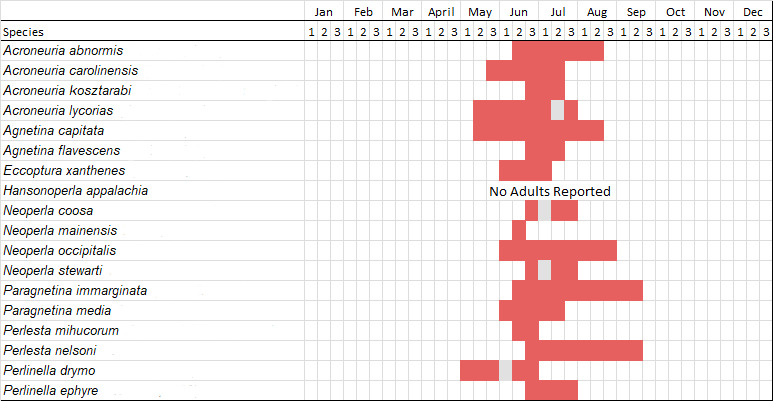
Adult flight period for 18 Perlidae species in New York State. Red fill indicates positive adult collections while gray shaded areas indicate adults are likely present but not reported.

**Figure 34.**
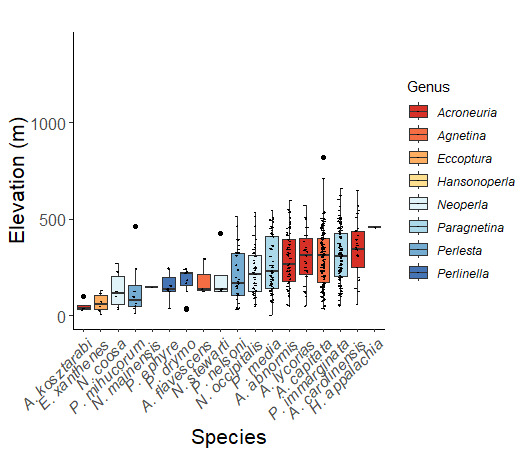
Elevation box plot for 18 Perlidae species in New York State. Boxes indicate interquartile range, horizontal line in box represents median elevation, and outlliers are depicted with large circles.

Perlidae are commonly referred to as the Stones (Stark et al. 1998, Stark et al. 2012). Nine genera and 18 species are known from the state. Stark (2004) provided the most recent review of the taxonomy of this family in eastern North America, but several species have been described during the past 20+ years and most notably in *Perlesta* (e.g., Kondratieff and Myers 2011). Stark (2017) presented larval keys to a limited number of species known from the southeastern USA, many of which are known from New York State. Adults have been collected in New York from early May through mid-September (Fig. 33) at elevations ranging from 0-821 m asl (Fig. 34). *Acroneuriakosztarabi*, *Eccopturaxanthenes*, and *Neoperlacoosa* appear to be restricted to lower elevations in the state (Fig. 34). Species known from smaller streams and rivers of higher elevations include *Acroneuriacarolinensis*, *Agnetinacapitata*, *Hansonoperlaappalachia*, and *Paragnetinaimmarginata*.

#### Acroneuria abnormis

(Newman, 1838)

4CF0E580-993C-5E76-9012-F72A747736D3

##### Notes

*Acroneuriaabnormis* is commonly referred to as the Common Stone (Stark et al. 2012). This is amongst the most widely distributed stonefly species in North America. In Canada, *A.abnormis* has been reported from the island of Newfoundland west to Alberta. In the USA, this species has been recorded from 37 of the 48 conterminous states (Stark 2004, DeWalt et al. 2024). For additional context, the lack of records from New Hampshire, New Jersey, and Vermont are likely due to information deficiency and not true absence. Peckarsky (1979) documented this species from large to medium streams across a wide geographical area. Bottorff and Knight (1987) conducted a detailed ecological study *A.abnormis*, suggesting a two-year life cycle for larvae in Michigan streams. Kondratieff and Despins (1983) reported a late May to mid-July flight period in Virginia. Our records from New York indicate a flight period extending from mid-June to mid-August (Fig. 33) at elevations from 51-597 m asl (Fig. 34). Although this species has been collected frequently from large streams and rivers across the eastern half of the state (Fig. 35a), further surveys in western New York should yield additional records.

#### Acroneuria carolinensis

(Banks, 1905)

F0319721-C621-5F9A-BE08-4C12AFF6194B

##### Notes

This species is commonly known as the Carolina Stone (Stark et al. 2012). The distribution of *A.carolinensis* extends from Quebec west to Manitoba and south to Alabama and South Carolina (Stark 2004, DeWalt et al. 2024). Sheldon (1985) reported this species from low elevations (500-700 m) in the Little River drainage in east Tennessee. Schmidt and Tarter (1985) reported a two-year life cycle, for *A.carolinensis* from a stream in West Virginia. In New York, adults of this species have been collected from early June through mid-July (Fig. 33), with a presence mainly in smaller streams and rivers at elevations ranging from 59-647 m asl (Fig. 34). This species is likely more common than current collections indicate, with most records from elevated regions of the state (Fig. 35b).

#### Acroneuria kosztarabi

Kondratieff & Kirchner, 1993

8CB948CE-39E4-59C2-AC0F-5702D7DF7742

##### Notes

*Acroneuriakosztarabi* is commonly referred to as the Virginia Stone (Stark et al. 2012). This species was known from a relatively small range from Ohio south to North Carolina and Tennessee (Verdone et al. 2022, DeWalt et al. 2024). Verdone et al. (2022) recently provided evidence that *Acroneuriakirchneri* Stark & Kondratieff, 2004 is a junior subjective synonym of this species. Historical reports of *A.trijuncta* (Walker, 1852) by Needham (1925) pertain to this species. In New York, adult collection dates for this species range from late June to mid-July (Fig. 33). These records represent a new state record and a significant northward range extension. Our records are from large, low elevation lakes (see below) at elevations ranging from 28-99 m asl (Fig. 34) from Level IV Ecoregions Eastern Adirondack Foothills (58ac) and Champlain Lowlands (83b) in northeastern New York (Fig. 35c). No recent collections of this species have been made in Lake George. We collected pre-emergent nymphs from the undersides of large rocks on south-facing exposed shorelines of Lake Champlain. Our failed attempts to collect this species from accessible areas of the shoreline in May and June over the course of several years suggest that the nymphs of this species may spend much of their life cycle in deeper waters that are inaccessible to standard collection techniques. In Virginia and North Carolina Verdone et al. (2022) found larvae of *A.kosztarabi* in pools and runs of rivers in areas of lower velocity.

#### Acroneuria lycorias

(Newman, 1839)

01A794E2-909C-5CC8-AB2D-80717B1CB660

##### Notes

This species is commonly referred to as the Boreal Stone (Stark et al. 2012). *Aconeurialycorias* has been reported in Canada from the Maritime Provinces west to Saskatchewan and in the USA from Maine west to Iowa and North Dakota plus south to Tennessee and North Carolina, with a disjunct distribution in Florida (Pescador et al. 2000, Stark 2004, DeWalt et al. 2024). Previous observations of this species have suggested a two to three year life cycle (Harper and Magnin 1969, Hitchcock 1974, Peckarsky 1979, Barton 1980). Peckarsky (1979) documented this species from a wide range of stream sizes, with adult emergence dates ranging from mid-April to early August. In New York, this species has been collected from mid-May to late July (Fig. 35d) from elevations ranging from 49-569 m asl (Fig. 34). This species is common in streams and large rivers across the state (Fig. 35d).

#### Agnetina capitata

(Pictet, 1841)

476FEB44-2B39-57A9-9933-FFAA0AA60B11

##### Notes

*Agnetinacapitata* is commonly known as the Northern Stone (Stark et al. 2012). The range of this widespread and common species extends in Canada from Newfoundland-Labrador and the Atlantic Maritime Provinces west to Manitoba and in the USA from Maine west to Minnesota and Iowa, and further south from Pennsylvania and Virginia west to Oklahoma (Stark 2004, DeWalt et al. 2024). Moreira and Peckarsky (1994) documented complex variation of a delayed egg hatch, resulting in cohort splitting into two and three years for a population in Tompkins County, New York. Harper (1973b) reported the emergence of this species from early June to late July in southern Ontario. In New York, adults of *A.capitata* have been collected between mid-May to mid-August (Fig. 33). This species was common in streams and rivers at elevations ranging from 25-821 m asl (Fig. 34) and is widely distributed and common across the state (Fig. 35e).

#### Agnetina flavescens

(Walsh, 1862)

B73226B5-17D7-5481-BE13-996E8D69B3C8

##### Notes

*Agnetinaflavescens* is commonly referred to as the Midwestern Stone (Stark et al. 2012). This species overlaps extensively in the USA with *A.capitata*, except it can be found further south to Alabama and Georgia (Stark 2004, DeWalt et al. 2024). Previous studies of this species indicated a two-year life cycle similar to *A.capitata* (Ernst and Stewart 1985). The flight period for *A.flavescens* in New York ranges from mid-June to mid-July (Fig. 33). Records are available from lower elevations ranging from 124-293 m asl (Fig. 34) only from Level IV Ecoregion Finger Lakes Uplands and Gorges (60d) (Fig. 35f). Further collections in the western portion of the state will likely yield additional distributional records.

#### Eccoptura xanthenes

(Newman, 1838)

590EBD3C-5E46-5507-8109-59352B13A4A7

##### Notes

This species is commonly known as the Yellow Stone (Stark et al. 2012), and has been reported from New York and Rhode Island south to Mississippi, Alabama, and Florida (Stark 2004, DeWalt et al. 2024). Previous life history studies have indicated a two-year life cycle for *E.xanthenes* in Kentucky (Allen and Tarter 1985). Kondratieff and Despins (1983) collected adults over a two-month period from early June to late July from a Virginia stream. Adults have been collected in New York from early June through early July (Fig. 33). Southern New York appears to be the northern range extent of this species, with records available from lowland areas at 7-131 m asl (Fig. 34) from Level IV Ecoregions Glaciated Reading Prong/Hudson Highlands (58i), Southern New England Coastal Plains and Hills (59c), Trap Rock and Conglomerate Uplands (64b), and Hackensack Meadowlands (64g) (Fig. 36a). With increased development and urbanization in these areas much of the suitable habitat for this species has and will likely continue to be degraded or eliminated.

#### Hansonoperla appalachia

Nelson, 1979

3CDF1FC7-3713-519B-BCBD-0DEF14D2C07E

##### Notes

*Hansonoperlaappalachia* is currently referred to as the Appalachian Stone (Stark et al. 2012). This is an Appalachian-distributed species that has been patchily reported from New Hampshire south to Tennessee and South Carolina (Stark 2004, DeWalt et al. 2024). NatureServe (2024) recently classified this species as vulnerable to extinction or extirpation throughout its North American range. Little is known about the life history of this species, except that larvae may spend most of their time in the hyporheic zone. Kirchner and Kondratieff (1985) collected pre-emergent larvae from tree roots exposed by undercut stream banks. Kondratieff and Kirchner (1996) reported adults of this species present from mid-May to mid-June. The adult flight period for this species is currently unknown for New York (Fig. 33). The single distinctive larva known from New York was collected from a small stream at 454 m asl (Fig. 34) in Level IV Ecoregion Catskills Transition (60c) (Fig. 36b) by Martin Ronsenfeld of the New York City Department of Environmental Protection on September 9, 2004, representing a new state record.

#### Neoperla coosa

Smith & Stark, 1998

D8EA75F1-853B-56D6-BA5F-5E24C7968D07

##### Notes

This species is commonly known as the Coosa Stone (Stark et al. 2012). Although published accounts of *N.coosa* are patchily available from New York south to Alabama and Georgia (Stark 2004, Verdone et al. 2017, DeWalt et al. 2024), the records presented here and an additional unreported specimen from Ontario at CSUIC suggests that this species range is likely further northward. In New York, adult specimens were collected from late June to late July (Fig. 33) from low elevation rivers at 31-269 m asl (Fig. 34) from Level IV Ecoregions of Glaciated Reading Prong/Hudson Highlands (58i), Eastern Adirondack Foothills (58ac), Catskills Transition (60c), Finger Lakes Uplands and Gorges (60d), Ontario Lowlands (83c), and Cape Cod/Long Island (84a) (Fig. 36c).

#### Neoperla mainensis

Banks, 1948

597B94CF-B8E5-5A79-B7CC-1FF0F40620CA

##### Notes

*Neoperlamainensis* is commonly referred to as the Maine Stone (Stark et al. 2012). This species is known only from historical records from Ontario (Pelee Point of Lake Erie) and in the USA in Illinois, Maine, New York, and Ohio (DeWalt et al. 2024). This species is presumed extirpated from Illinois (DeWalt and Grubbs 2011) and Ohio (DeWalt et al. 2012). Our record is based on Cornell University specimens collected on June 11, 1919 (Fig. 33) at 146 m asl (Fig. 34) from the Roeliff Jansen Kill, a tributary to the Hudson River in Level IV Ecoregion Taconic Foothills (58x) (Fig. 36d).

#### Neoperla occipitalis

(Pictet, 1841)

E80E9FBB-FBE0-5999-88AF-2F4EA79308EF

##### Notes

*Neoperlaoccipitalis* is commonly referred to as the Atlantic Stone (Stark et al. 2012). Similar to *N.coosa*, this species is also patchily distributed in eastern North America. Records extend from Nova Scotia and in the USA from New York west to Wisconsin and Illinois and south to South Carolina, Alabama, and Mississippi (Stark 2004, DeWalt et al. 2024). Based on our examination of material at CUIC, some historical records of *N.clymene* from New York State are applicable to this species. In New York, adults of this species were collected commonly at lights near larger rivers. Adult collection dates for this species range from late May to early August (Fig. 33) from rivers at elevations ranging from 50-535 m asl (Fig. 34) and broadly across the state (Fig. 36e).

#### Neoperla stewarti

Stark & Baumann, 1978

A899D970-C455-5E0C-9CE6-419874B374CF

##### Notes

*Neoperlastewarti* is commonly known as the Multispine Stone (Stark et al. 2012). This species is widely distributed in eastern North America, with records from Pennyslvania southwest to Mississippi and west across the Laurentian Great Lakes region to Illinois and Minnesota (Stark 2004, DeWalt et al. 2024). Adults of this species have been collected in New York from late June through late July (Fig. 33), representing a new state record but expected since there are additional records from Ohio, Massachusetts, and Maine (DeWalt et al. 2024). Reported elevations for this species range from 125-427 m asl (Fig. 34) from Level IV Ecoregions Northern and Western Adirondack Foothills (58ab) and Finger Lakes Uplands and Gorges (60d) (Fig. 36f).

#### Paragnetina immarginata

(Say, 1823)

4B22438C-EFCD-5498-B5D6-8DEB77F6BC6B

##### Notes

*Paragnetinaimmarginata* is commonly referred to as the Beautiful Stone (Stark et al. 2012). The distribution of this large and common species extends from Quebec and Maine south along the Appalachian Mountains to Tennessee and Georgia (Stark 2004, DeWalt et al. 2024). Little life history information exists for *P.immarginata*. Smith (1913) conducted a preliminary observational study of this species in New York over a three-month period in a tributary to Coy Glen (Tompkins County). Johnson (1981) reported dietary overlap between larvae of *P.immarginata* and *A.capitata* in Orwell Brook (Oswego County). Harper and Pilon (1970) found that this species exhibited an extended emergence period from early July to late August in Quebec. In New York, collections of adults range from mid-June through mid-September (Fig. 33) at elevations ranging from 50-658 m asl (Fig. 34) from larger streams and rivers across the state (Fig. 37a).

#### Paragnetina media

(Walker, 1852)

B399AB54-8EBB-570B-9746-637B8A356F61

##### Notes

*Paragnetinamedia* is commonly known as the Embossed Stone (Stark et al. 2012). The range of this widespread species extends from New Brunswick northwest to the Northwest Territories, west to Minnesota and Iowa, and southwest to Arkansas (Hart et al. 2025, Stark 2004, DeWalt et al. 2024). A two-year life cycle has been reported for *P.media* in Michigan (Heilman and Knight 1970) and Kentucky (Tarter and Krumholz 1971). Harper (1973b) reported a three-year life cycle and parthenogenetic females in southern Ontario. In New York, adults have been collected between early June and mid-July (*Fig. 33*) from larger streams and rivers at elevations ranging from 50-658 m asl (Fig. 34) and is broadly distributed across much of the state (Fig. 37b).

#### Perlesta mihucorum

Kondratieff & Myers, 2011

28919C82-0EF8-5767-9F04-04CBEFBFAA47

##### Notes

This species is commonly referred to as the New York Stone (Fig. 1, Stark et al. 2012), with distributional records known only from New York and Maryland (Grubbs 2018, Kondratieff and Myers 2011). Adults have been collected in the state from mid- to late June (Fig. 33) at elevations ranging from 10-463 m asl (Fig. 34) from rivers in Level IV Ecoregions Acid Sensitive Adirondacks (58aa), Eastern Adirondack Foothills (58ac), Hudson Valley (59i), and Glaciated Low Allegheny Plateau (60a) (Fig. 37c).

#### Perlesta nelsoni

Stark, 1989

D6654B6A-CB0A-5431-9CB1-78095EE89436

##### Notes

*Perlestanelsoni* is commonly known as the Pale Stone (Stark et al. 2012). This is mainly an Appalachian-distributed species reported from New York south to Tennessee and South Carolina (Stark 2004, DeWalt et al. 2024). In New York, adults of this species have been collected between late June through mid-September (Fig. 33) from elevations ranging from 32-514 m asl (Fig. 34). This species appears to be relatively common in larger streams and small rivers in Level III Ecoregions Northeastern Highlands (58), Northeastern Coastal Lowlands (59), Northern Allegheny Plateau (60), and Eastern Great Lakes Lowlands (83) (Fig. 37d).

#### Perlinella drymo

(Newman, 1839)

AA52BB02-59BD-5243-B957-0CC5304CAFAE

##### Notes

This species is commonly referred to as the Striped Stone (Stark et al. 2012). *Perlinelladrymo* is distributed broadly across much of the eastern half of North America, with records from Quebec and Nova Scotia west to Minnesota, southwest to Texas, and south to Florida (Kondratieff et al. 1988, Stark 2004, DeWalt et al. 2024). The life history of this species remains unstudied, but Stewart and Stark (2002) suggested that larvae of this genus probably spend a large portion of their life cycle in the hyporheic zone. In New York, adults have been collected between early May through late June (Fig. 33) from low elevations at 32-241 m asl (Fig. 34). We found populations along wave-swept shorelines of large lakes and seasonally inundated floodplains of large rivers in Level IV Ecoregions Eastern Adirondack Foothills (58ac), Finger Lakes Uplands and Gorges (60d), Champlain Lowlands (83b), Upper St. Lawrence Valley (83e), and Mohawk Valley (83f) (Fig. 37e).

#### Perlinella ephyre

(Newman, 1839)

94B19483-974E-5D49-8A03-819BD1602115

##### Notes

*Perlinellaephyre* is commonly known as the Vernal Stone (Stark et al. 2012). This widespread species is known from all states east of the Mississippi River, plus from Minnesota south to Louisiana and west into Kansas and Oklahoma (Stark 2004, DeWalt et al. 2024). In New York, adults emerge later than *P.drymo*, between mid-June and late July (Fig. 33) from low elevations ranging from 36-246 m asl (Fig. 34). Records are available from Level IV Ecoregions Finger Lakes Uplands and Gorges (60d) and Champlain Lowlands (83b) (Fig. 37f). This species was encountered in similar habitats to those noted above for *P.drymo*.

#### Perlodidae

Klapálek, 1909

4264474A-5071-52BB-A3C3-A3C6BA0C3DD0

##### Notes

**Figure 38.**
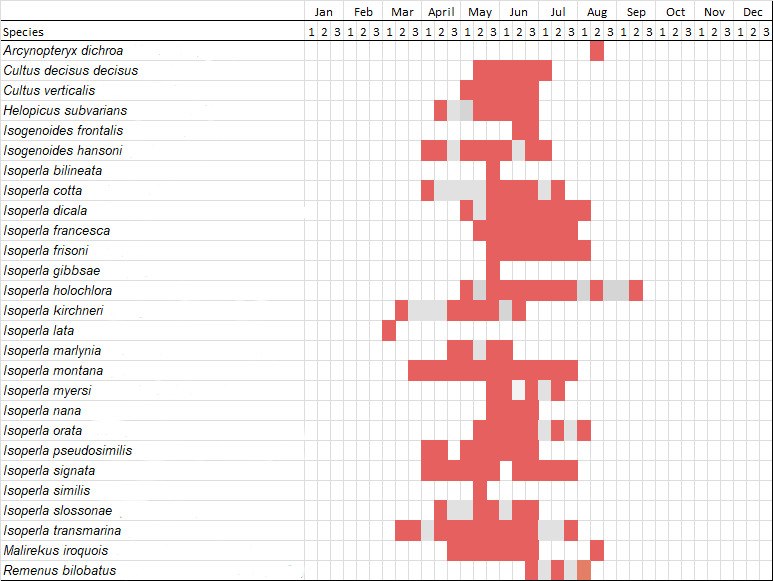
Adult flight period for 28 Perlodidae species in New York State. Red fill indicates positive adult collections while gray shaded areas indicate adults are likely present but not reported.

**Figure 39.**
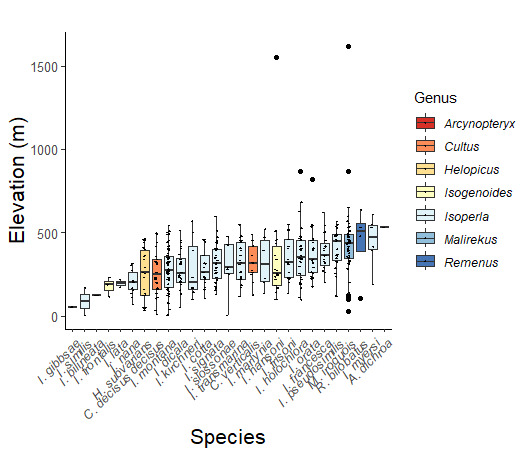
Elevation box plot for 28 Perlodidae species in New York State. Boxes indicate interquartile range, horizontal line in box represents median elevation, and outlliers are depicted with large circles.

Perlodidae are commonly known as Stripetails (subfamily Isoperlinae) and Springflies (subfamily Perlodinae) (Stark et al. 1998, Stark et al. 2012). This is the richest stonefly family in New York, comprised of seven genera and 28 species, including 19 *Isoperla*. Kondratieff (2004) and Szczytko and Kondratieff (2015) provided the most recent reviews on the Perlodinae and Isoperlinae of eastern North America, respectively. Larval identifications of *Isoperla* are still problematic for several eastern North American species. Beaty (2015) provided a useful photographic guide to the larvae of several species that occur in New York. Some genera of this family are commonly collected, whereas others are rarely encountered. Adult collection dates range from early March to September (Fig. 38). Perlodidae occupy a wide range of elevations in New York ranging from 3-1621 m asl (Fig. 39). Species occupying lower elevations in the state include *Isoperlabilineata*, *I.similis*, and *I.gibbsae*. Two species with the widest range of reported elevations include *Malirekusiroquois* and *Isogenoideshansoni*. Conversely, species with narrow ranges of reported elevations include uncommon species with few occurences (e.g., *Arcynopteryxdichroa*, *I.gibbsae*, *I.lata*, and *I.bilineata*).

#### Arcynopteryx dichroa

(McLachlan, 1872)

D5E77C49-2613-526C-9160-78C0E152C7AC

##### Notes

*Arcrynopteryxdichroa* is commonly known as the Holarctic Springfly (Stark et al. 2012). This is a circumpolar, northern Holarctic species that was previously referred to in North America as *A.compacta* (McLachlan, 1872) (Kondratieff 2004, Teslenko 2012, Numanović et al. 2024). In North America, the distribution of this species extends from the central Rocky Mountains north to a broad belt from Alaska east to Nunavut, with isolated records from Lake Superior in Michigan, New York, New Hampshire, and Maine (DeWalt et al. 2024). Stewart (1990) reported a univoltine life cycle for this species in Alaska. Larvae of *A.dichroa* typically inhabit rocky streams and rocky, wave-swept lake shorelines. This species, however, has not been collected in New York since August 1905 (Fig. 38) at 530 m asl (Fig. 39). This sole report is from Old Forge in Level IV Ecoregion Acid Sensitive Adirondacks (58aa) (Fig. 40a) under the old name *Perlodeslineata* (Smith, 1917) (Needham and Claassen 1925). The New York specimen has also been referred to as. *A.minor* Klapalek, 1912 (Hanson 1942). Further surveys targeting suitable high elevation habitats in the Adirondacks are needed to determine its status in the state.

#### Cultus decisus

(Walker, 1852)

FDD9F881-3728-5D87-A328-B860A9741671

##### Notes

This species is commonly referred to as the Great Lakes Springfly (Stark et al. 2012). Stark et al. (1988) proposed two disjunct subpsecies of *C.decisus* - northern *C.d.decisus* and southern *C.d.isolatus* (Banks, 1920). For this treatment, we have adopted the more inclusive species treatment. *Cultusdecisus* sensu lato is known patchily from New Brunswick west to Michigan and south to Georgia (Stark et al. 1988, Kondratieff 2004, Myers and Kondratieff 2009, DeWalt et al. 2024). The life history and biology of the eastern *Cultus* remains poorly known (Stewart and Stark 2002). Larval descriptions of *C.d.decisus*, *C.d.isolatus*, and *C.verticalis* are currently available (Myers and Kondratieff 2009). Adult records in New York for *C.decisus* are available from mid-May through late July (Fig. 38) at elevations of 10-499 m asl (Fig. 39). This species was common in larger rivers in the Level IV Ecoregions Catskill High Peaks (58y), Adirondack High Peaks (58z), Eastern Adirondack Foothills (58ac), Central Adirondacks (58ad), Hudson Valley (59i), Delaware-Neversink Highlands (60b), Champlain Lowlands (83b), and Mohawk Valley (83f) (Fig. 40b).

#### Cultus verticalis

(Banks, 1920)

D11F325D-8856-5C7B-81EA-C7D7CF7C76B4

##### Notes

*Cultusverticalis* is commonly referred to as the Spiny Springfly (Stark et al. 2012). The distribution of this species extends from Quebec south along the Appalachian Mountains to Georgia (Kondratieff 2004, Myers and Kondratieff 2009, Verdone et al. 2017, DeWalt et al. 2024). Adult collection dates in New York range from early May to late June (Fig. 38). This species inhabits smaller streams compared to *C.decisus*, with records at elevations ranging from 126-489 m asl (Fig. 39). State records are from Level IV Ecoregions Catskill High Peaks (58y), Northern and Western Adirondack Foothills (58ab), Eastern Adirondack Foothills (58ac), Central Adirondacks (58ad), Tug Hill Plateau (58ae), Tug Hill Transition (58af), and Upper St. Lawrence Valley (83e) (Fig. 40c).

#### Helopicus subvarians

(Banks, 1920)

A02D46CB-D1F1-54CC-96A7-99CE865057FC

##### Notes

This species is commonly known as the Vernal Springfly (Stark et al. 2012), and is broadly distributed from New Brunswick west to Ontario and south to Alabama and Florida (Kondratieff 2004, DeWalt et al. 2024). Stark and Ray (1983) suggested a univoltine life cycle for this species in Florida. In Ontario, emergence of *H.subvarians* was recorded from late May to early June (Harper and Pilon 1970). In New York, adults have been collected from mid-April and extending to late June (Fig. 38). State records are available from elevations ranging from 34-464 m asl (Fig. 39). Nymphs were commonly collected in the spring months from larger rivers in the Level III Ecoregions Northeastern Highlands (58), Northern Allegheny Plateau (60), and Eastern Great Lakes Lowlands (83) (Fig. 40d).

#### Isogenoides frontalis

(Newman, 1838)

105C79AC-6521-5F46-A9A1-1D509609FA9B

##### Notes

*Isogenoidesfrontalis* is commonly referred to as the Hudsonian Springfly (Stark et al. 2012). This species is very broadly distributed in Canada from Newfoundland-Labrador and the Atlantic Maritime Provinces west to Saskatchewan and parallel in the USA from Maine west to Minnesota (Kondratieff 2004, Sandberg and Stewart 2005, DeWalt et al. 2024). Hilsenhoff and Billmyer (1973) reported *I.frontalis* from small, high gradient streams in Wisconsin streams and suggested a univoltine life cycle. The limited, scattered reports of adults of this species in New York are available from mid- to late June (Fig. 38) at elevations ranging from 115-231 m asl (Fig. 39) from Level IV Ecoregions Eastern Adirondack Foothills (58ac), Ontario Lowlands (83c), and Mohawk Valley (83f) (Fig. 40e).

#### Isogenoides hansoni

(Ricker, 1952)

A7B8872B-19A8-50EC-A252-620A64A3A282

##### Notes

This species is commonly known as the Appalachian Springfly (Stark et al. 2012), and is known from Quebec and the Canadian Maritime Provinces, southwest into the USA along the Appalachian Mountains to North Carolina (Kondratieff 2004, Sandberg and Stewart 2005, DeWalt et al. 2024). Based on material examined at the CUIC, previous reports of *Isogenoidesdoratus* (Frison, 1942) from New York are applicable to *I.hansoni*. In New York, adults were present from early April through early July (Fig. 38) at elevations ranging from 100-1551 m asl (Fig. 39). This species is more widely distributed and commonly collected compared to *I.frontalis*, with scattered records from Level III Ecoregions Northeastern Highlands (58), Northern Allegheny Plateau (60), North Central Appalachians (62), and Eastern Great Lakes Lowlands (83) (Fig. 40f).

#### Isoperla bilineata

(Say, 1823)

CF430B1D-2C84-52EF-9ADC-1C7E4B0AB8B2

##### Notes

*Isoperlabilineata* is commonly referred to as the Two-lined Stripetail (Stark et al. 2012). This species is widespread across the eastern Nearctic, with records in Canada from Newfoundland west to Saskatchewan and southward in the USA to Mississippi, Alabama, and Florida, and west to Colorado (Szczytko and Kondratieff 2015, DeWalt et al. 2024). The sole record for this species representing the first report of this species in the state is based on a single ungravid female collected in late May 1926 (Fig. 38) from an approximate elevation of 125 m asl (Fig. 39) from Level IV Ecoregion Finger Lakes Uplands and Gorges (60d) (Fig. 41a). Additional material is needed to determine the status of this species in New York.

#### Isoperla cotta

Ricker, 1952

418D2890-001F-58FB-B548-7FCE6C73FC04

##### Notes

*Isoperlacotta* is commonly known as the Ontario Stripetail (Stark et al. 2012). This species is distributed from the Canadian Maritime Provinces west across the Laurentian Great Lakes region to Wisconsin and further south in the USA with disjunct records from West Virginia and Virginia (Szczytko and Kondratieff 2015, DeWalt et al. 2024). Hilsenhoff and Billmyer (1973) recorded this species from small to medium sized streams in Wisconsin. Harper (1973b) reported a univoltine-slow life cycle with emergence from to mid-June to early July from southern Ontario. In New York, adults of *I.cotta* were collected from early April to mid-July (Fig. 38). We have found this species from a wide range of stream sizes ranging in elevation from 107-461 m asl (Fig. 39) from the Level IV Ecoregions Glaciated Reading Prong/Hudson Highlands (58i), Acid Sensitive Adirondacks (58aa), Eastern Adirondack Foothills (58ac), Tug Hill Plateau (58ae), and Tug Hill Transition (58af) (Fig. 41b).

#### Isoperla dicala

Frison, 1942

F1B47043-71C5-5223-9309-9A82F310AFF0

##### Notes

This species is commonly referred to as the Sable Stripetail (Stark et al. 2012). *Isoperladicala* is distributed extensively across eastern North America, reported from most Canadian Provinces and USA states both east and immediately west of Hudson Bay and the Mississippi River southward along the appalachians to South Carolina (Szczytko and Kondratieff 2015, Verdone et al. 2023, DeWalt et al. 2024). Harper and Pilon (1970) documented emergence of *I.dicala* in late June from Ontario. In Virginia, adults were collected from late May to early July using a black light trap (Kondratieff and Despins 1983). In New York, adults of *I.dicala* have been reported from early May to early August (Fig. 38) from elevations ranging from 53-512 m asl (Fig. 39). This species was collected frequently from larger streams and rivers in Level III Ecoregions Northeastern Highlands (58) and Eastern Great Lakes Lowlands (83), and likely has a broader distribution than current collections indicate (Fig. 41c).

#### Isoperla francesca

Harper, 1971

5AF59D20-6607-5E5E-AC45-1E66769AC4E0

##### Notes

*Isoperlafrancesca* is commonly known as the Northeastern Stripetail (Stark et al. 2012). This species has been reported infrequently in northeastern North America, with records available from Nova Scotia east to Quebec and southwest to Pennsylvania (Szczytko and Kondratieff 2015, DeWalt et al. 2024). Harper et al. (1991b) suggested a univoltine-slow life cycle for this species in Quebec. We report an adult flight period in New York from mid-May through late July (Fig. 38). Populations of *I.francesca* have been documented at elevations ranging from 203-620 m asl (Fig. 39) from a broad range of stream sizes from Level IV Ecoregions Adirondack High Peaks (58z), Acid Sensitive Adirondacks (58aa), Northern and Western Adirondack Foothills (58ab), Eastern Adirondack Foothills (58ac), Central Adirondacks (58ad), Tug Hill Plateau (58ae), and Upper St. Lawrence Valley (83e) (Fig. 41d).

#### Isoperla frisoni

Illies, 1966

08F308BA-02F4-50B6-B87C-3C225A3350B7

##### Notes

*Isoperlafrisoni* is commonly referred to as the Wisconsin Stripetail (Stark et al. 2012). This is a widspread species reported in Canada from Nova Scotia west to Manitoba. In the USA it occurs from Maine west across the Laurentian Great Lakes region to Minnesota, plus south mainly along the Appalachian Mountains to Georgia (Szczytko and Kondratieff 2015, DeWalt et al. 2024). Harper and Magnin (1969) suggested a univoltine-slow life cycle for a population from Quebec, with adults present from late May to early July. In New York, adults of *I.frisoni* have been collected between late May through early August (Fig. 38) at elevations ranging from 134-548 m asl (Fig. 39) from larger rivers and streams from Level IV Ecoregions Acid Sensitive Adirondacks (58aa), Eastern Adirondack Foothills (58ac), Central Adirondacks (58ad), Champlain Lowlands (83b), St. Lawrence Lowlands (83d), Upper St. Lawrence Valley (83e), and Mohawk Valley (83f) (Fig. 41e).

#### Isoperla gibbsae

Harper, 1971

BFD929EA-6A18-5039-8EC8-61C6F74AFD85

##### Notes

This species is currently known as the Quebec Stripetail (Stark et al. 2012). *Isoperlagibbsae* has been reported from scattered locations from Quebec south to New York and Connecticut, with unsubstantiated records from Maryland and West Virginia (Szczytko and Kondratieff 2015, DeWalt et al. 2024). In New York, a small series of adults was collected on May 21, 1967 (Fig. 38) at 54 m asl (Fig. 39) from the town of Highland in Level IV Ecoregion Hudson Valley (59i) (Fig. 41f). The lack of any verified recent collections of this species may be cause for concern.

#### Isoperla holochlora

Klapálek, 1923

9951C93E-F88C-5300-AB16-2CF00E58A897

##### Notes

*Isoperlaholochlora* is currently known as the Pale Stripetail (Stark et al. 2012). This is a common, Appalachian-distributed species reported from Quebec and the Canadian Maritime Provinces southwest to Alabama and Georgia (Szczytko and Kondratieff 2015, DeWalt et al. 2024). Mackay (1969) noted a univoltine-slow larval life cycle for *I.holochlora* in Quebec. We report an adult flight period in New York extending from early May through mid-September (Fig. 38) with populations reported at elevations ranging from 97-866 m asl (Fig. 39). In New York, this species was collected frequently from Level III Ecoregions Northeastern Highlands (58), Northern Allegheny Plateau (60), and Eastern Great Lakes Lowlands (83) and is likely also present in upland areas of western New York (Fig. 42a).

#### Isoperla kirchneri

Szczytko & Kondratieff, 2015

C2FEDCD9-9C0F-5C01-9E34-3C77E11A3EBC

##### Notes

This species is currently referred to as the Two-spine Stripetail (Stark et al. 2012). *Isoperlakirchneri* is primarily an Appalachian-distributed species reported from New York south to Tennessee and North Carolina (Szczytko and Kondratieff 2015, DeWalt et al. 2024). Szczytko and Kondratieff (2015) suggested that this species has a univoltine-slow life cycle. In New York, adults of *I.kirchneri* have been collected from mid-March through mid-June (Fig. 38) at elevations of 101-570 m asl (Fig. 39) from streams and rivers in Level IV Ecoregions Catskill High Peaks (58y), Eastern Adirondack Foothills (58ac), Glaciated Low Allegheny Plateau (60a), Champlain Lowlands (83b), and Upper St. Lawrence Valley (83e) (Fig. 42b). This species is likely more common and widespread in the state.

#### Isoperla lata

Frison, 1942

7C64E2F2-B47F-58AF-9000-114AD04220DF

##### Notes

*Isoperlalata* is currently known as the Dark Stripetail (Stark et al. 2012). This species has been reported from Canada from mainland Labrador and Nova Scotia west to Ontario and in the USA from Maine in parallel fashion west to Minnesota plus southwest along the Appalachian Mountains to North Carolina and Tennessee (Szczytko and Kondratieff 2015, DeWalt et al. 2024). Sandberg and Szczytko (1997) suggested a univoltine-slow larval life cycle for *I.lata* in central Wisconsin. In Quebec, adults have been reported from early May to late June (Harper and Magnin 1969). The limited adult flight period data in New York of only early March (Fig. 38) is based on reared material. We report collections of this species from elevations ranging from 170-218 m asl (Fig. 39) from the Boquet River in Level IV Ecoregions Eastern Adirondack Foothills (58ac) and Champlain Lowlands (83b) (Fig. 42c). This species is likely more common in the state than collections indicate and is easily distinguished as larvae from other members of this genus by the distinct lacinia setation.

#### Isoperla marlynia

(Needham & Claassen, 1925)

DF6FB870-0694-5C31-BF92-637BEB52B71D

##### Notes

This species is currently referred to as the Midwestern Stripetail (Stark et al. 2012). *Isoperlamarlynia* is a broadly distributed species reported from Nova Scotia west to Saskatchewan, southwest to Colorado, and sporadically south to South Carolina (Szczytko and Kondratieff 2015, DeWalt et al. 2024). Larvae are often found clinging to submerged wood in riffles of larger streams (Szczytko and Kondratieff 2015). *Isoperlamarlynia* is presumably extirpated from Illinois and Indiana (DeWalt and Grubbs 2011, DeWalt et al. 2005). The adult filight period for *I.marlynia* in New York extends from late April to early June (Fig. 38). Populations have been found at elevations ranging from 136-522 m asl (Fig. 39) from Level IV Ecoregions Acid Sensitive Adirondacks (58aa), Eastern Adirondack Foothills (58ac), Central Adirondacks (58ad), Tug Hill Transition (58af), and Upper St. Lawrence Valley (83e) (Fig. 42d).

#### Isoperla montana

(Banks, 1898)

1B035CD4-4F67-503C-AB67-6758DC1C9DB8

##### Notes

*Isoperlamontana* is currently known as the Montane Stripetail (Stark et al. 2012). This species has been reported from Nova Scotia west to Ontario, Minnesota, and Indiana, and southeast along the Appalachian Mountains to Virginia (Szczytko and Kondratieff 2015, Verdone and Kondratieff 2017, DeWalt et al. 2024). Harper et al. (1991b) found that larvae of *I.montana* displayed a univoltine-slow life cycle in Quebec. In their study, early instar larvae first appeared in June and July directly following the emergence of adults in May. In New York, the adult flight period extends from late March through late July (Fig. 38). *Isoperlamontana* has been reported from elevations ranging from 3-544 m asl (Fig. 39) and appears to be widely distributed across the state (Fig. 42e).

#### Isoperla myersi

Szczytko & Kondratieff, 2015

E1F260B5-48A1-5216-B7B4-A8DFFE67EED2

##### Notes

This species is commonly known as the Paddle Stripetail (Stark et al. 2012) and has only been reported in New York State (Szczytko and Kondratieff 2015, DeWalt et al. 2024). However, collections from Pennsylvania and Rhode Island (unpublished data) suggest this species may be more widespread than presently understood. The adult flight period of *I.myersi* is from late May to mid-July (Fig. 38). The elevation range for this species ranges from 189-609 m asl (Fig. 39), with records available from Level IV Ecoregions Catskill High Peaks (58y), Acid Sensitive Adirondacks (58aa), Eastern Adirondack Foothills (58ac), Central Adirondacks (58ad), and Champlain Lowlands (83b) (Fig. 42f). Although this species is currently listed as an SGCN in New York (New York State Department of Environmental Conservation 2015), it may be more common in the eastern USA than presently understood. Adult females, mature eggs, and larvae are currently undescribed.

#### Isoperla nana

(Walsh, 1862)

65310A9B-726B-5CD1-BB0C-FE23860DFD7C

##### Notes

*Isoperlanana* is currently referred to as the Least Stripetail (Stark et al. 2012) and is known from Ontario and Quebec southwest through the Laurentian Great Lakes region to Kentucky (Szczytko and Kondratieff 2015, DeWalt et al. 2024). In Wisconsin, this species has been reported from small streams and exhibited a univoltine life cycle (Hilsenhoff and Billmyer 1973). In New York, adult collection dates range from late May through late June (Fig. 38). This species occupies lowland streams ranging in elevation from 69-317 m asl (Fig. 39) with records available from Level III Ecoregions Northeastern Highlands (58), Northeastern Coastal Lowlands (59), Northern Allegheny Plateau (60), and Eastern Great Lakes Lowlands (83) (Fig. 43a).

#### Isoperla orata

Frison, 1942

53DC4250-B39C-5D37-ACB7-C739DAE61980

##### Notes

*Isoperlaorata* is commonly known as the Colorless Stripetail (Stark et al. 2012). This species is known from Canada from Nova Scotia west to Quebec and in the USA from Maine southwest mainly along the Appalachian Mountains to Tennessee and South Carolina, with a disjunct distribution present in Minnesota (Szczytko and Kondratieff 2015, DeWalt et al. 2024). Harper and Pilon (1970) reported the emergence of this species in early June in Quebec. In New York, adults have been collected between mid-May through early August (Fig. 38) from a wide range of elevations (175-821 m asl; Fig. 39). Although this species is known mainly from Level III Ecoregions Northeastern Highlands (58), Northern Allegheny Plateau (60), and Eastern Great Lakes Lowlands (83) (Fig. 43b), further collections in western New York would likely yield additional records.

#### Isoperla pseudosimilis

Szczytko & Kondratieff, 2015

D092489A-9F5B-5408-BDED-135AF6D88F98

##### Notes

This species is commonly referred to as the Confusing Stripetail (Stark et al. 2012). *Isoperlapseudosimilis* is an Appalachian-distributed species reported from Maine southwest to Tennessee and North Carolina (Szczytko and Kondratieff 2015, DeWalt et al. 2024). *Isoperlapseudosimilis* is a common species in New York and prior to 2015 most records were referred to as *I.similis*. Adults have been collected in New York from early April through late June (Fig. 38) from elevations ranging from 119-480 m asl (Fig. 39), with numerous records from elevated regions across the state (Fig. 43c). This species is especially common in small spring-fed streams.

#### Isoperla signata

(Banks, 1902)

4238824B-ED74-5EED-95F3-9F92A7205489

##### Notes

This species is commonly referred to as the Transverse Stripetail (Stark et al. 2012) and has been recorded from Canada from Nova Scotia west to Manitoba (Szczytko and Kondratieff 2015, DeWalt et al. 2024). The distribution of the species in the USA, however, is more complex. Records are available from Maine south to Virginia and west to Minnesota and in turn south to Arkansas and Oklahoma (Hart et al. 2025, Szczytko and Kondratieff 2015, DeWalt et al. 2024). Ecological studies of *I.signata* have indicated a univoltine-slow larval life cycle (Krueger and Cook 1981, Jop and Szczytko 1984) from Minnesota and Wisconsin, respectively. Hilsenhoff and Billmyer (1973) reported this species from a wide range of stream sizes in Wisconsin. Adult collection dates for this species in New York range between early April and late July (Fig. 38) from elevations ranging from 131-597 m asl (Fig. 39) from small to medium-sized rivers in Level III Ecoregions Northeastern Highlands (58), Northern Allegheny Plateau (60), and Eastern Great Lakes Lowlands (83) (Fig. 43d).

#### Isoperla similis

(Hagen, 1861)

FF0CF0BF-05F8-5CBF-8729-1131402A0FD7

##### Notes

*Isoperlasimilis* is currently known as the Black Stripetail (Stark et al. 2012). Although this species is presently considered as distributed from Quebec and Nova Scotia south to North Carolina and Tennessee (Szczytko and Kondratieff 2015, DeWalt et al. 2024), prior records, especially from the Appalachian Mountains, need to be reassessed for accuracy due to the recent description of *I.pseudosimilis*. Szczytko and Kondratieff (2015) provided confirmation of this species from six states and noted that *I.similis* is mainly present in the Piedmont Plateau and the Atlantic Coastal Plain. Our limited collections of this species in New York have found adults in mid-May (Fig. 38). All confirmed New York localities are from elevations ranging from 119-549 m asl (Fig. 39), but only from Level IV Ecoregions Catskills Transition (60c) and Cape Cod/Long Island (84a) (Fig. 43e). Additional collecting on Long Island and other remnant relatively pristine streams of New York’s Atlantic Coastal Plain is needed to determine the conservation status of this species in the state.

#### Isoperla slossonae

(Banks, 1911)

6EC015BB-2319-5B6E-B7AE-88F32400FAA9

##### Notes

This species is currently known as the Colorful Stripetail (Stark et al. 2012) and has been recorded in Canada from Quebec and the Canadian Maritime Provinces south in the USA to North Carolina and from Michigan west to Minnesota and Iowa (Szczytko and Kondratieff 2015, DeWalt et al. 2024). Hilsenhoff and Billmyer (1973) reported a univoltine life cycle for this species in small to medium-sized streams in Wisconsin. We report adult collection dates in New York for *I.slossonae* from mid-April to late June (Fig. 38). Although this species was not reported from New York by Szczytko and Kondratieff (2015), we have confirmed records at low elevations ranging from 3-169 m asl (Fig. 39) from streams and rivers in Level IV Ecoregions Taconic Foothills (58x), Adirondack High Peaks (58z), Eastern Adirondack Foothills (58ac), Central Adirondacks (58ad), and Cape Cod/Long Island (84a) (Fig. 43f).

#### Isoperla transmarina

(Newman, 1838)

F516702D-8E72-55FE-BC79-0BDE0FAF434C

##### Notes

*Isoperlatransmarina* is commonly referred to as the Boreal Stripetail (Stark et al. 2012). This species is distributed in Canada from Newfoundland-Labrador west to British Columbia. In the USA, *I.transmarina* has been reported from Maine across the Laurentian Great Lakes region to Colorado and south to North Carolina (Szczytko and Kondratieff 2015, DeWalt et al. 2024). Harper et al. (1991b) reported a univoltine-slow life cycle for this species in Quebec. Eggs hatched directly with larvae first appearing in June and July, followed by a two-week adult emergence beginning in mid-May. We report an adult flight period for *I.transmarina* in New York that extends from mid-March through late July (Fig. 38). Reported elevations for this species in the state range from 119-548 m asl (Fig. 39) with records primarily from larger streams and rivers in Level III Ecoregions Northeastern Highlands (58) and Eastern Great Lakes Lowlands (83) (Fig. 44a).

#### Malirekus iroquois

Stark & Szczytko, 1988

ABD3BAA4-D926-50EA-8514-37371895C482

##### Notes

*Malirekusiroquois* is commonly referred to as the Iroquois Springfly (Stark et al. 2012). This species has been reported in Canada from Nova Scotia west to Canada and in the USA from New Hampshire and Vermont southwest to Maryland and Ohio (Kondratieff 2004, DeWalt et al. 2024). No detailed ecological studies have been conducted on *M.iroquois*, although Huryn and Wallace (1987) reported a univoltine-slow life cycle for closely related *M.hastatus* (Banks, 1920) in North Carolina. Our collections from New York indicate a flight period extending from late April through mid-August (Fig. 38). This species has been found in small rocky streams and rheocrenes at elevations ranging from 99-1620 m asl (Fig. 39) and is widely distributed across upland areas of the state (Fig. 44b).

#### Remenus bilobatus

(Needham & Claassen, 1925)

98628E44-8090-5B07-9A13-A6C76F10A501

##### Notes

This species is commonly known as the Lash Springfly (Stark et al. 2012) with distributional reports from Connecticut and New York south along the Appalachian Mountains to Alabama and Georgia (Kondratieff 2004, Verdone and Kondratieff 2018, DeWalt et al. 2024). The life history and ecology of *R.bilobatus* are unknown. Although originally described from New York, few collections of this species have been made since its initial description from Old Forge in Herkimer County. Records of this species in the state indicate a late summer emergence, with adults collected from late June through early August (Fig. 38). We have collected both nymphs and adults of *R.bilobatus* at elevations ranging from 106-637 m asl (Fig. 39) from small spring-fed streams in Level IV Ecoregions Eastern Adirondack Foothills (58ac) and Central Adirondacks (58ad) (Fig. 44c).

#### Pteronarcyidae

Newman, 1853

A5A469B0-8B56-5F15-86AC-EE6B765084BE

##### Notes

**Figure 45.**
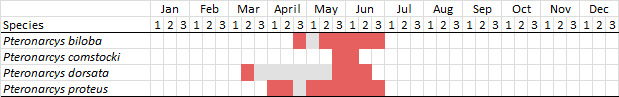
Adult flight period for 4 Pteronarcyidae species in New York State. Red fill indicates positive adult collections while gray shaded areas indicate adults are likely present but not reported.

**Figure 46.**
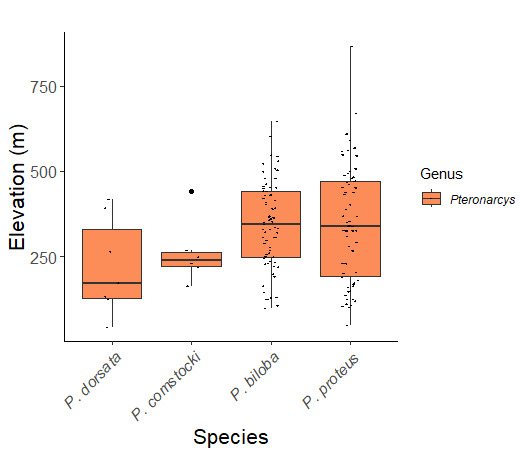
Elevation box plot for 4 Pteronarcyidae species in New York State. Boxes indicate interquartile range, horizontal line in box represents median elevation, and outlliers are depicted with large circles.

Pteronarcyidae are commonly referred to as Salmonflies (Stark et al. 1998, Stark et al. 2012) and are among the largest-bodied stoneflies in North America. One genus with four species have been reported from New York (Myers et al. 2011). Nelson (2000) provided the most recent review of this family for eastern North America. Myers and Kondratieff (2017) provided descriptions and an updated key to the larvae from North America. Larval exuviae can often be found great distances from streams and rivers. Adults of this family have been recorded from mid-March through late June (Fig. 45). Pteronarcyidae in New York occupy a wide range of elevations from 41-866 m asl (Fig. 46).

#### Pteronarcys biloba

Newman, 1838

BEBAD790-0EF8-5B4B-8BDA-2331F85A8F20

##### Notes

*Pteronarcysbiloba* is commonly known as the Knobbed Salmonfly (Stark et al. 2012). This species has been reported from Quebec and the Canadian Maritime Provinces southeast mainly through the Appalachian Mountains to Alabama and Georgia (Nelson 2000, DeWalt et al. 2024). This species is widely distributed across New York State (Fig. 44d), with an adult flight period extending from late April through late June (Fig. 45) from a wide range of streams and rivers at elevations ranging from 98-647 m asl (Fig. 46).

#### Pteronarcys comstocki

Smith, 1917

3F65C78D-AA55-509E-A91A-B89F2805C549

##### Notes

This species is commonly referred to as the Spiny Salmonfly (Stark et al. 2012). *Pteronarcyscomstocki* is an uncommon species distributed patchily from New Brunswick southeast to Kentucky and Tennessee (DeWalt et al. 2024, Nelson 2000). In New York, this species is known from scattered localities in Level IV Ecoregions Adirondack High Peaks (58z), Acid Sensitive Adirondacks (58aa), Eastern Adirondack Foothills (58ac), Finger Lakes Uplands and Gorges (60d), and Mohawk Valley (83f) (Fig. 44e). Adults have been collected in the state from late May to early June (Fig. 45) and appear to prefer cold pristine rivers and larger streams at elevations ranging from 164-442 m asl (Fig. 46).

#### Pteronarcys dorsata

(Say, 1823)

817E8649-6173-5C97-9F42-CEF9ABA82F06

##### Notes

*Pteronarcysdorsata* is commonly known as the American Salmonfly (Stark et al. 2012). The range of this transcontinental species extends from Labrador and New Brunswick west across Canada to Alaska and south to Wyoming and from south from Maine to the USA Gulf Coastal region from Florida west to Louisiana (Nelson 2000, DeWalt et al. 2024). Barton (1980) suggested a three-to-four-year life cycle for this species in Alberta. In contrast, Lechleitner and Kondratieff (1983) suggested a univoltine life cycle for this species in Virginia. In New York, this species has been collected from across the state and is largely associated with large streams and rivers (Fig. 44f). Adults have been collected sporadically in the state from mid-March through mid-June (Fig. 45) at elevations ranging from 41-418 m asl (Fig. 46).

#### Pteronarcys proteus

Newman, 1838

74C4BFB2-656D-553D-B890-DCCBD934C8FE

##### Notes

**Figure 47.**
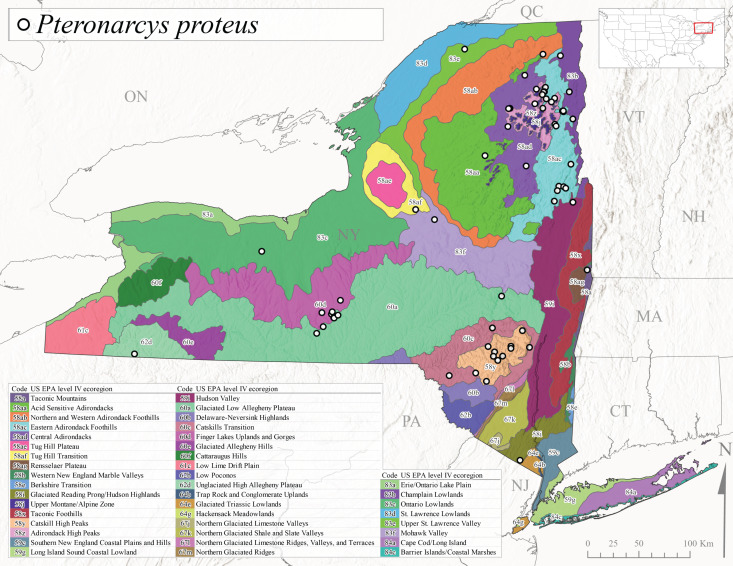
New York distribution maps of *Pteronarcysproteus*.

*Pteronarcysproteus* is commonly referred to as the Appalachian Salmonfly (Stark et al. 2012). This Appalachian-distributed species has been reported from Quebec south to Kentucky and North Carolina (Nelson 2000, DeWalt et al. 2024). Life history studies of *P.proteus* in Virginia (Perry et al. 1987) and Pennsylvania (Grubbs and Cummins 1996) have indicated a three to four year cycle. Grubbs and Cummins (1996) reported the emergence of adults in Pennsylvania from May through June. We report an adult flight period for *P.proteus* in New York extending from early April through the end of June (Fig. 45). Adults and larvae have been collected from a wide range of streams and rivers at elevations ranging from 48-866 m asl (Fig. 46) and are both widely distributed and common across the state (Fig. 47).

## Analysis

### Overview of state fauna

**Figure 48.**
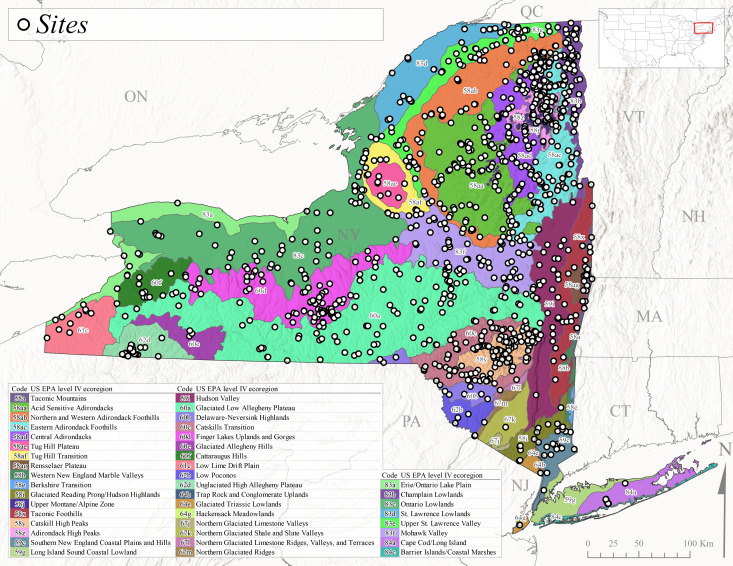
Distribution of unique collection locations across New York State overlayed onto USEPA Level IV Ecoregions.

**Figure 49.**
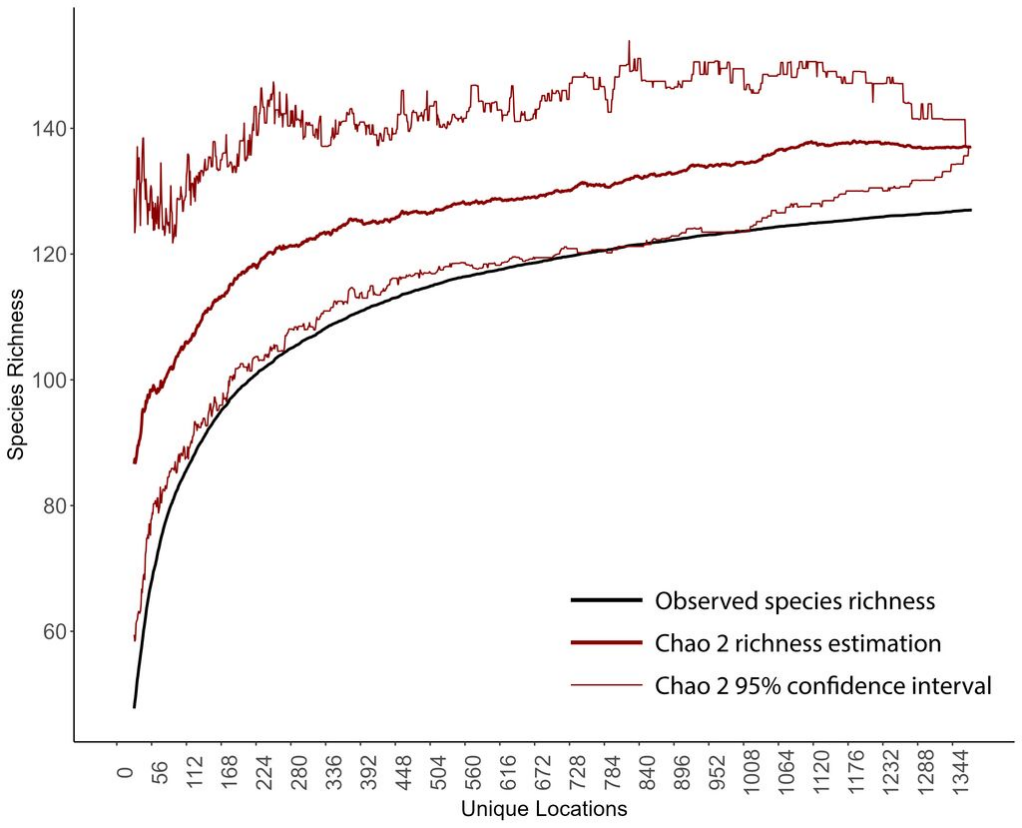
Chao 2 richness estimates and observed richness for stonefly species in New York State.

Over the course of this study we compiled 6,538 records of stoneflies from New York State. Nearly 32,000 individual specimens were examined from 1475 unique locations (Fig. 48), comprising 127 species across 42 genera and 9 families. The species accumulation curve for the full extent of the state shows a slightly increasing pattern with a Chao2 richness estimation leveling off at 137 species with no variance in 95 percent confidence intervals (Fig. 49). We provide the first reports of eight species from the state: *Leuctraalexanderi*, *L.carolinensis*, *Amphinemuraappalachia*, *Alloperlaleonarda*, *Acroneuriakosztarabi*, *Hansonoperlaappalachia*, *Neoperlamainensis*, and *N.stewarti*

**Figure 50.**
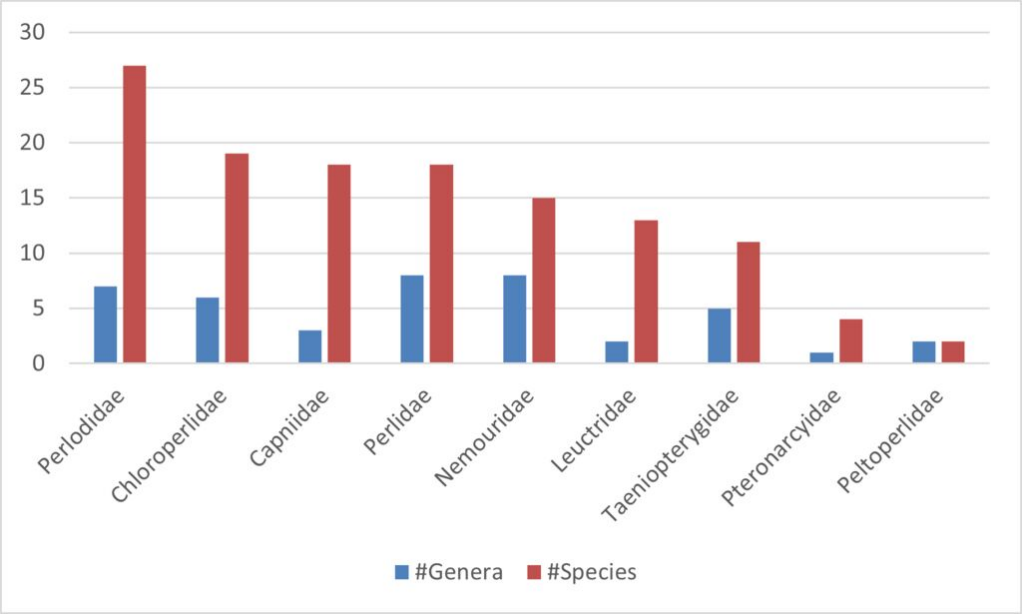
Genus and species level richness of stonefly families recorded from New York State.

The family Perlodidae has the highest reported richness with verified occurrences of 27 species and 7 genera (Fig. 50). The family Chloroperlidae ranked second in richness with 19 species and 6 genera reported, followed closely by Capniidae (18 species and 3 genera), Perlidae (18 species and 8 genera), Nemouridae (15 species and 8 genera), and Leuctridae (13 species and 2 genera) (Fig. 50). The family Peltoperlidae had the lowest diversity with two species and two genera, while the family Pteronarcyidae was represented by a single genus with four species (Fig. 50). Four species-rich genera, *Isoperla* (19 species), *Allocapnia* (15 species), *Leuctra* (12 species) and *Alloperla* (10 species) represent 44% of the current 127 species reported from the state (Fig. 50).

### State coverage and richness by elevation

**Figure 51.**
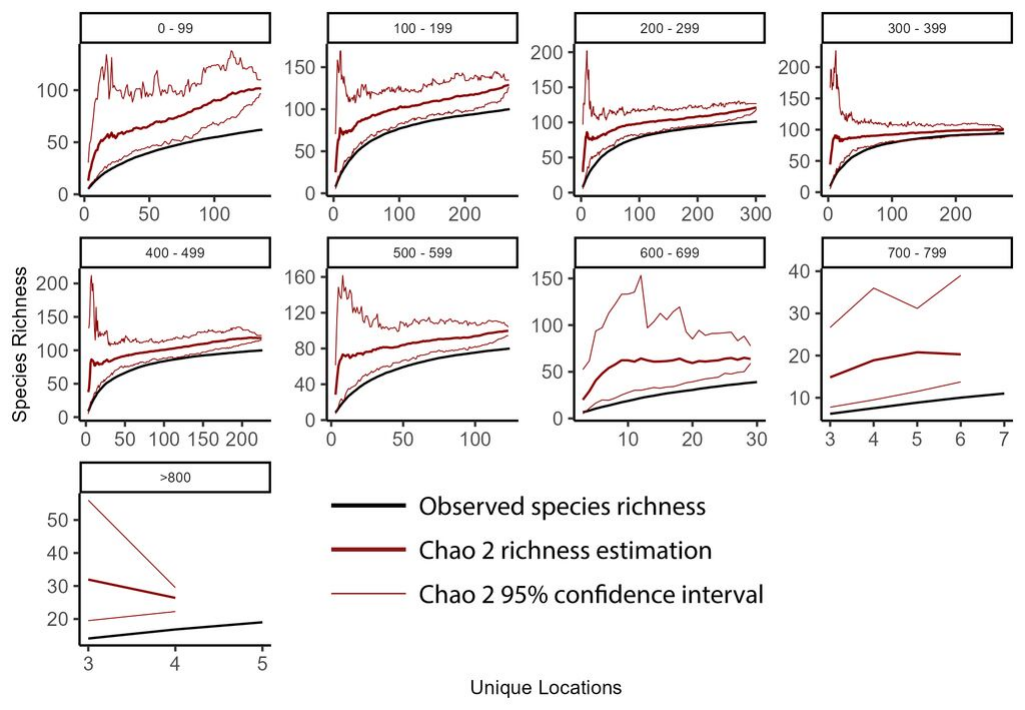
Species accumulation curves plotting observed species richness Chao2 species richness estimation and 95% confidence interval by individual 100 m elevation bands up to 800 m asl and for > 800-1500 m asl combined.

**Figure 52.**
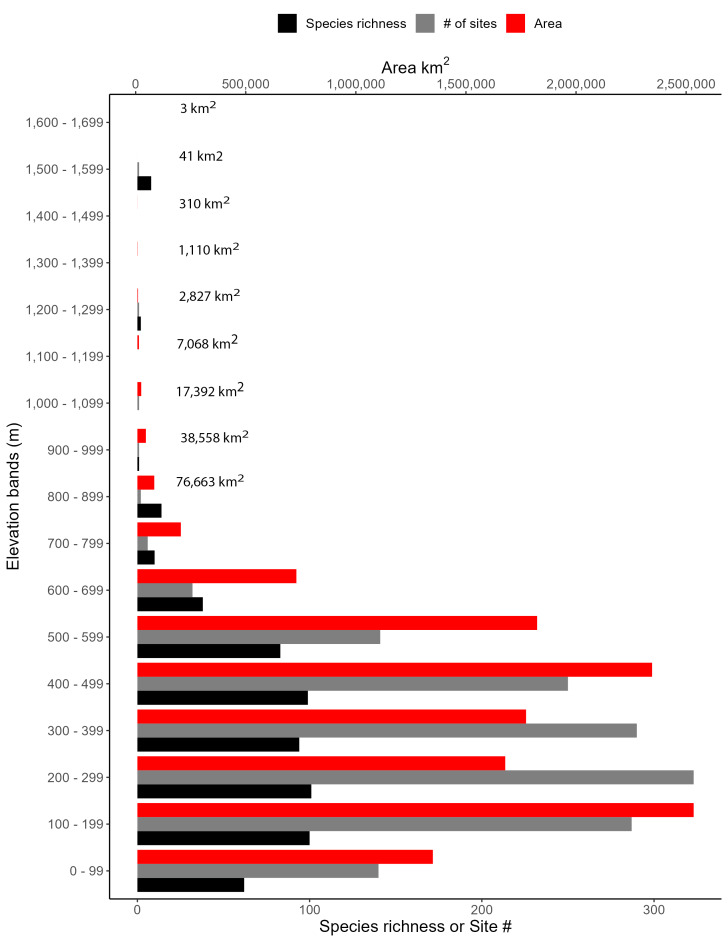
Summary of species richness, number of unique collection sites, total land area by elevation band in New York State.

With one general exception, species accumulation curves are shown in 100 m elevation bands ranging from 0-1500 m asl (Fig. 51). Due to the paucity of collection sites above 800 m, we chose to lump together those seven elevation bands 800 m and above. Given the wide range of elevations present in the state (0-1628 m asl), it may appear surprising that 87% of the 1475 unique locations sampled are at elevations below 500 m asl (Fig. 52), but this range occupies 77% of the total land area in New York State (Fig. 52).

The lowest individual elevation band (0-100 m asl) had the smallest observed richness at 62 species (Fig. 52). However, it is likely that more species will be recovered from these areas with further sampling efforts (Fig. 51). Observed richness increased to 100 species at the 100-200 m asl, 101 species at the 200-300 m asl, 94 species at 300-400 m asl, and 99 species at 400-500 m asl (Fig. 52). The 83 species reported from 500-600 m asl (141 unique locations) was markedly higher than the 62 species recorded from 0-100 m asl but with nearly the same number of sites (140 unique locations) (Fig. 52). The 300-399 m asl elevation band appears to be the most well sampled with a near flattening of the observed richness with increasing number of unique sites. There is a similar pattern observed with the 200-299 and the 400-499 m asl elevation bands. Further divergence of observed and estimated richness (indicating the potential for increases in observed richness with increased sampling) occurred at elevations below 299 m asl and above 500 m asl (Fig. 51). At sites above 800 m asl it is clear our knowledge of high elevation inhabitants and the upper elevation limits of many of these species is limited. Specific family and species-specific elevation trends are further summarized in the species accounts by family section.

### State coverage and richness patterns by ecoregion

**Figure 53.**
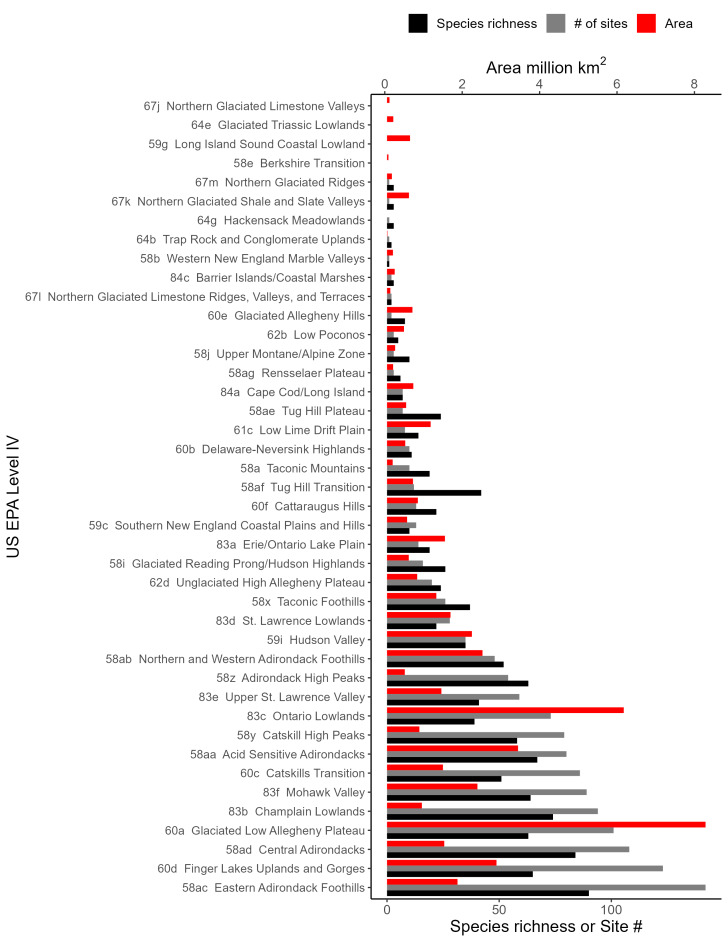
USEPA Level IV Ecoregions, land area, unique collection sites, and species richness. Ecoregion are presented in increasing order by the number of unique collection locations.

**Figure 54.**
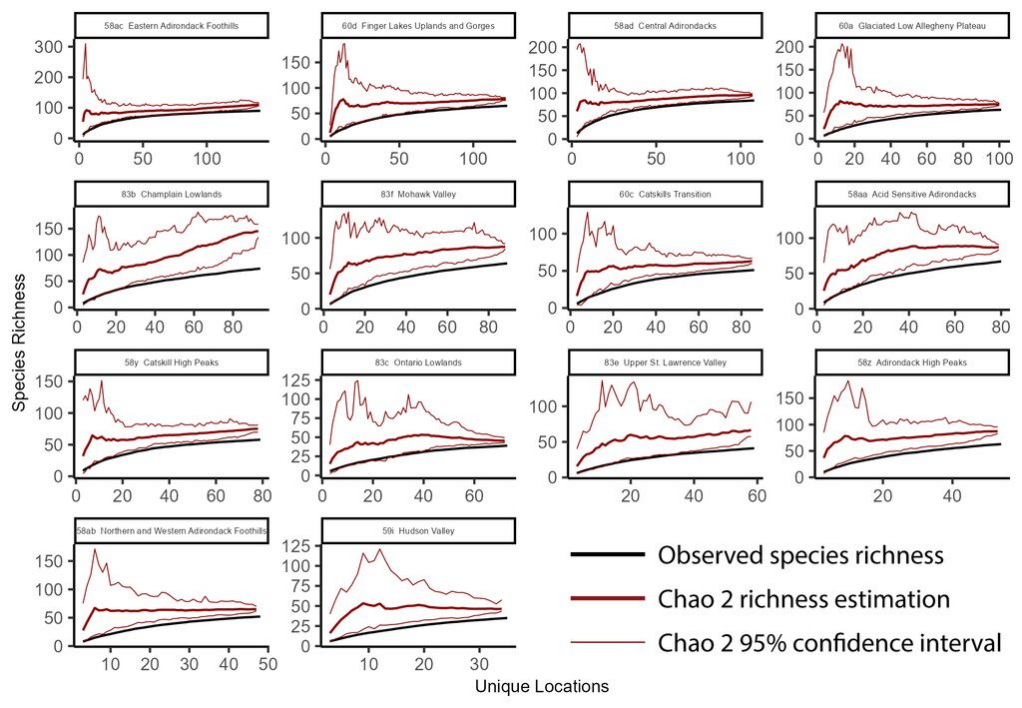
Species accumulation curves plotting observed richness, Chao2 richness estimator, and 95% confidence interval for 14 best sampled USEPA Level IV Ecoregions in New York. Ecoregions are presented in increasing order of unique collection locations, from left-to-right and top-to-bottom.

**Figure 55.**
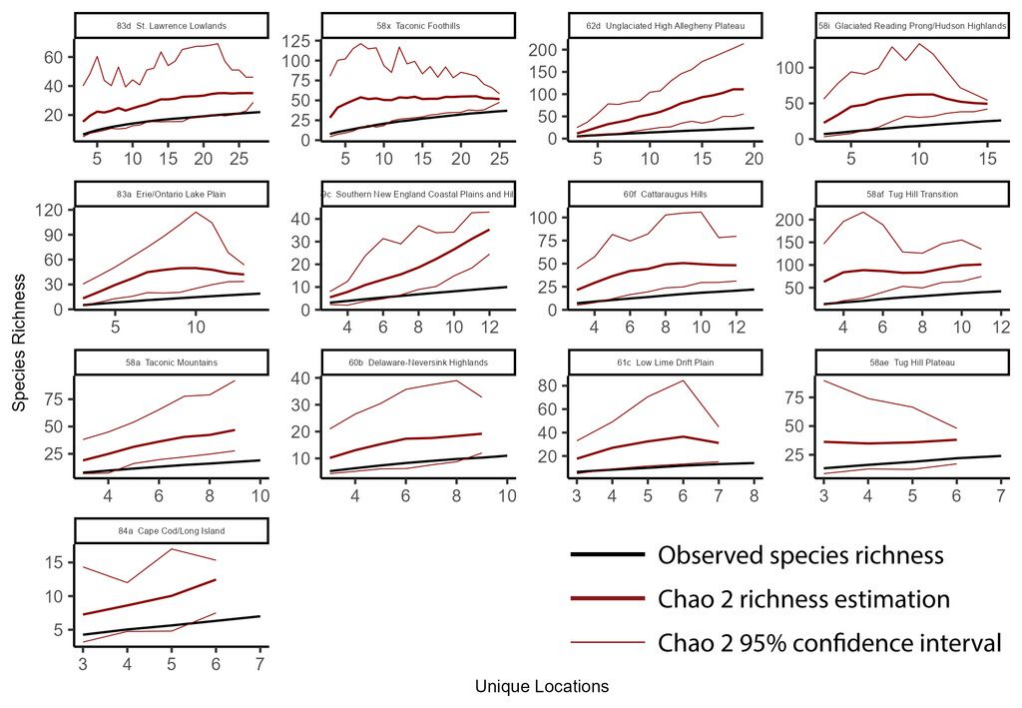
Species accumulation curves plotting observed richness and Chao2 richness estimator for the next 13 best sampled USEPA Level IV Ecoregions in New York. Ecoregions are presented in increasing order of unique collection location, from left-to-right and top-to-bottom.

In total, 38 of the 42 Level IV Ecoregions present in the state have one or more unique locations with at least one stonefly species, and 18 Ecoregions had >15 unique locations (Fig. 53). The four Ecoregions lacking stonefly records are Berkshire Transition (58c), Long Island Sound Coastal Lowland (59c), Glaciated Triassic Lowlands (64c), and Northern Glaciated Limestone Valleys (67j) (Fig. 53). With the exception of 59c, we anticipate that stoneflies are present in these ecoregions. The most species-rich ecoregions in New York State to date are the Eastern Adirondack Foothills (58ac; 90 species), Central Adirondacks (58ad; 84 species), Champlain Lowlands (83b; 74 species), Acid Sensitive Adirondacks (58aa; 67 species), and Finger Lakes Uplands and Gorges (60d; 65 species) (Fig. 53). There was a strong correlation between sampling effort (number of unique locations) (Figs 53, 54, 55) and observed richness patterns (b=0.62, p<0.001, r^2^=0.88). Even in the most well-collected Level IV Ecoregions (e.g., Eastern Adirondack Foothills (58ac)) the curve of observed richness has not yet reached its asymptope nor an intersection with Chao2 richness estimation indicating the potential for increases in richness with additional sampling effort (Fig. 54).

Observed richness appears to asymptote and reach a congruence with Chao 2 richness estimates indicating adequate sampling effort for eight Level IV Ecoregions: Eastern Adirondack Foothills (58ac), Finger Lakes Uplands and Gorges (60d), Central Adirondacks (58ad), Glaciated Low Allegheny Plateau (60a), Catskills Transition (60c), Ontario Lowlands (83c), Catskill High Peaks (58y), and Northern and Western Adirondack Foothills (58ab). This pattern is also present but less pronounced for the Mohawk Valley (83f), Acid Sensitive Adirondacks (58aa), Upper St. Lawrence Valley (83e), and Hudson Valley (59i) ecoregions.

### State coverage and richness patterns by watershed

**Figure 56.**
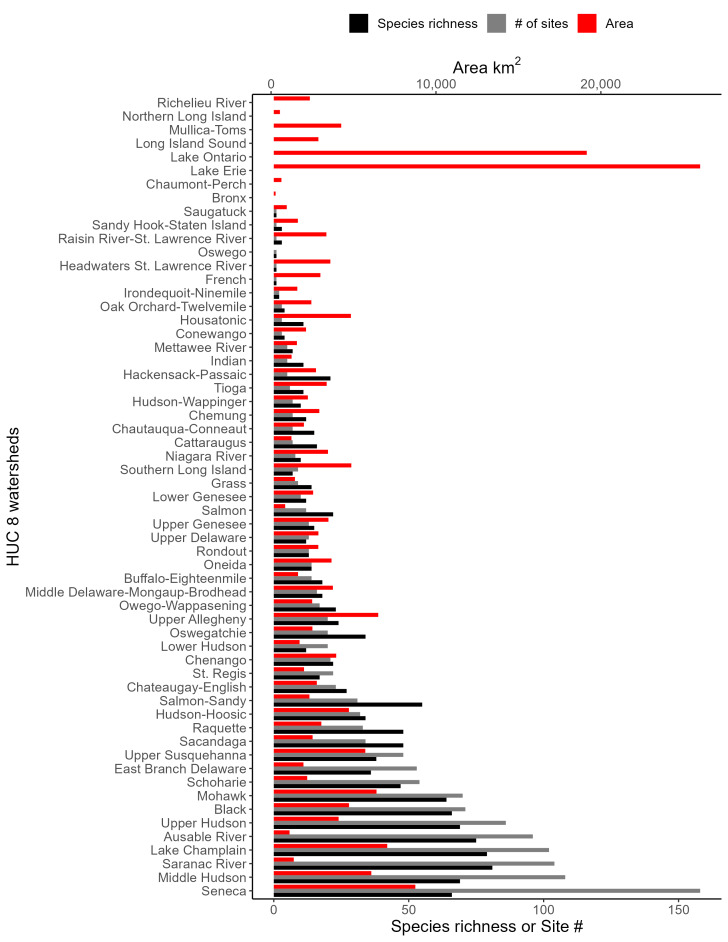
Species richness, number of unique collection localities, and drainage area for each of the HUC8 watersheds present in New York State. Watersheds are presented in order from top to bottom by increasing number of localities.

**Figure 57.**
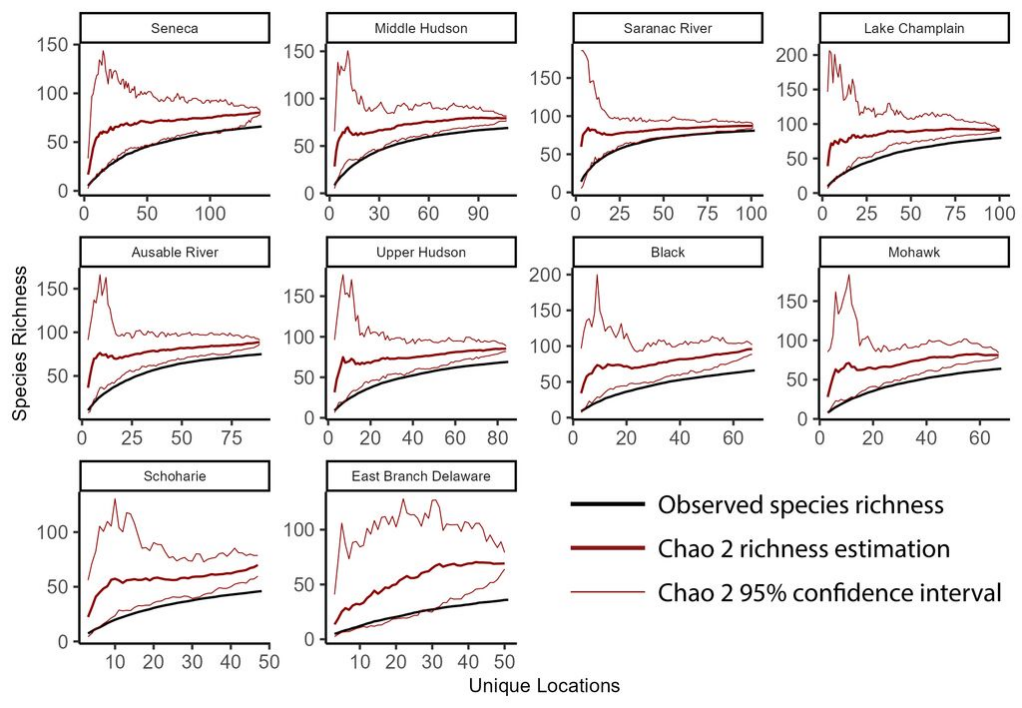
Species accumulation curves ordered by number of unique sample locations plotting observed richness, Chao 2 richness estimation, and 95% confidence intervals for 14 best sampled HUC8 watersheds in New York State. Watersheds are presented in descreasing number of unique collection locations from left-to-right and top-to-bottom.

Of the 59 HUC 8 watersheds in New York, only these eight have no reported collections of stoneflies: Bronx, Northern Long Island, Chaumont-Perch, Richelieu River, Long Island Sound, Mullica-Toms, Lake Ontario, and Lake Erie (Fig. 56). Several factors have likely contributed to the paucity of records from these watersheds including urbanization (Mullica-Toms, Bronx, and Northern Long Island), agriculture (Chaumont-Perch, Richelieu), and the restricted HUC8 boundaries of Lake Ontario and Lake Erie that exclude tributaries. The highest richness was observed in the Saranac River (81 species), Lake Champlain (79 species), Ausable River (75 species), Middle Hudson (69 species), Upper Hudson River (69 species), Black (66 species), Seneca (66 species), and Mohawk (64 species) HUC8 watersheds (Fig. 56). The most well sampled watersheds include the Seneca (158 unique locations), Middle Hudson (108), Saranac River (104), Lake Champlain (102), Ausable River (96), Upper Hudson (86), Black (54), and Schoharie (54) (Fig. 56). The relationship between sampling effort (number of unique sites) and species richness is apparent (b=0.64, p<0.001, r^2^=0.83). For example, the Saranac River (104) was heavily collected over the course of this study and appears to be adequately sampled with richness estimations nearly convergent with the flattening curve plotting observed richness. This trend is repeated but less pronounced in Seneca, Middle Hudson, Lake Champlain, Ausable River, and Upper Hudson HUC8 watershed. The effect of increased sampling effort appears to be less pronounced in larger watersheds (i.e., Seneca) (Fig. 57).

### Rare and historical species

The paucity of records for several species that we consider as "rare" may simply be an artifact of recent vs. historical collection efforts in the state. Four species are listed as Species of Greatest Conservation Need (SGCN) in the 2015 New York State Wildlife Action Plan (WAP) (New York State Department of Environmental Conservation 2015): *Allocapniaillinoensis*, *A.ohioensis*, *Isoperlagibbsae*, and *Isogenoidesfrontalis*. Similarly, six species are included in the "Rare Animal Status List" by the New York Natural Heritage Program, including five not listed in the NY WAP (New York Natural Heritage Program 2017): *A.illinoensis*, *Ostrocercacomplexa*, *Alloperlavoinae*, *A.vostoki*, *Utaperlagaspesiana*, and *Pteronarcyscomstocki*. None of the six aforementioned species have state conservation rankings.

Seventeen species reported from New York are considered to be species of conservation concern and are currently listed as SGCN on the 2025 draft SGCN list and have been recommended for listing as SGCN on the updated 2025 NY WAP (New York State Department of Environmental Conservation 2025): *Allocapniaillinoensis*, *A.ohioensis*, *Leuctramaria*, *Ostrocercaprolongata*, *Alloperlaleonarda*, *A.voinae*, *A.vostoki*, *Rasvenaterna*, *Utaperlagaspesiana*, *Acroneuriakosztarabi*, *Hansonoperlaappalachia*, *Neoperlamainensis*, *Arcynopteryxdichroa*, *Isoperlagibbsae*, *Isogenoidesfrontalis*, and *Pteronarcyscomstocki*.

Eleven species in total, including two listed above, have not been collected since prior to 1970 and are considered herein as "historical": *Allocapniaindianae*, *A.ohioensis*, *A.zola*, *Leuctracarolinensis*, *L.triloba*, *Peltoperlaarcuata*, *Neoperlamainensis*, *Arcynopteryxdichroa*, *Isogenoidesfrontalis*, *Isoperlagibbsae*, and *I.bilineata*. Some of these species might be lost from the state, but concerted effort is required to document extirpation. Often, it is difficult to conduct a statewide survey for all species and do enough work to document the loss of specific species.

We were not able to locate any specimens collected in the state to corroborate previous reports by Needham and Claassen (1925) of *Acroneuriaarenosa*, *A.arida*, and *Attaneuriaruralis*. A historical record of *Perlaramosa* (Needham and Claassen, 1925), a nomen dubium (DeWalt et al. 2024), was not included in our treatment. Based on our examination of material at the Cornell University Collection, the Needham (1925) report of *Acroneuriatrijuncta* from Lake George, represents the more recently described *Acroneuriakosztarabi*. Frison (1942) reported *Isogenoidesdoratus* from three locations in New York. However, these records represent the more common species *Isogenoideshansoni* that was described 10 years later. *Perlestalagoi* Stark, 1989, *Perlesta* n. sp. 1, and *Perlesta* n. sp. 2 were listed in error by Myers et al. (2011) and now refer to *P.mihucorum*.

## Discussion

This is the fifth holistic treatment of the stonefly fauna of a USA state, following similar frameworks on the faunas of Arkansas (Hart et al. 2025), Indiana (Newman et al. 2021), Maryland (Hogan and Grubbs 2022), and Ohio ([Bibr B10429403][Bibr B10429365]). Over the course of this study we compiled 6,571 records from 51 HUC8 watersheds and 38 USEPA Level IV Ecoregions, following a long history of stonefly research in the state that includes the designations of 28 primary types.

Certain regions have received more attention than others, namely in the vicinity of Ithaca with its extensive entomological history at Cornell University and along lower elevational bands of the Adirondack and Catskill mountains. Even in these two seemingly well-collected montane regions, efforts have been concentrated mainly along easily accessible roadways and at trailheads. Future work focused within a combination of less-collected HUC8s, Level IV Ecoregions, and elevation bands will likely yield new regional and state records (e.g., *Acroneuriaarenosa* and *Paranemouraclaasseni*). For example, 31 species have been reported after 2008 from New York, including 23 from the Adirondacks (Myers et al. 2011) and eight from this study. Much of this effort during the past 17 years has focused on areas in the eastern portion of the state, reflecting, in part, the geographic location of the first author.

With warming temperatures, cold water adapted species such as stoneflies are expected to experience a significant loss of available habitats, especially at lower elevations <800 m asl, resulting in range reductions up to 96% (Bojková et al. 2012). This loss of habitat may cause significant upward elevation shifts that will create "summit traps" leading to eventual species loss once the species reaches the elevational limits of available habitat (Sauer et al. 2011). For example, over a 30-year period Sheldon (2012) observed a significant rate of upslope migration for *Acroneuriaabnormis* (24 m/decade) from a small watershed in Great Smoky Mountains National Park, Tennessee USA. Since 1970, New York has seen an average annual increase in air temperature of 0.6℉ per decade with winter warming in excess of 1.1℉ per decade (New York State Department of Environmental Conservation 2021), with current models that suggest some areas of New York State will see a 12℉ increase in annual average temperatures by the year 2100. These concerns highlight the need to obtain further information in the state on the elevation limits of these species in mountainous areas.

The paucity of records above 800 m asl reported from this study is largely due to the lack of maintained roads above this elevation, but there are extensive trail networks across the Adirondacks and Catskills (White et al. 2005, Heilman et al. 2019) that provide ready access to higher-elevation streams and lake outlets. Two recent studies on the stonefly faunas of Mammoth Cave National Park, Kentucky, USA (McRoberts and Grubbs 2021) and Mount Mitchell, North Carolina, USA (Metzger and Grubbs 2023) each relied heavily on hiking trails and permanently closed roads to access most of their collection sites. Future sampling at higher montane elevations in New York will be especially important to assess the upper elevation limits of several species (especially at elevations >800 m asl).

The Adirondacks, in particular, contain many boreal species at or near the southern limits of their range (e.g., *Podmostamacdunnoughi* and *Capnuramanitoba*. Conversely, several species (e.g., *Leuctraalexanderi* and *Leuctratriloba*) have Appalachian affinites and reach the northern distributional limits in the Appalachian Plateau with some extending into lowland valleys into northern regions of the state and onward into the Canadian provinces. Other species have clear affinities with the midwestern states and have colonized the state through the Ohio River drainage (e.g. *Allocapniaindianae* and *Taeniopteryxmetequi*), some with distributions extending into Canada on the low-lying areas surrounding the Adirondack Park.

Large data gaps also exist for areas of western New York (Allegany and Letchworth state parks), Long Island (Connetquot State Park and other smaller municipal and county owned parks and preserves), the Lower Hudson Valley, and north of the New York Metropoliton Area (e.g., Catskill Park, and Harriman State Park, and protected areas of the NYC watershed owned and regulated by the New York City Department of Environmental Protection). These include some of the last remaining protected habitats in a highly urbanized region of the state.

The negative effects of urbanization on pollution intolerant aquatic insects has been well documented (Morse et al. 2003). In some cases, it appears that riparian buffers have limited some of the negative effects of land use on water quality in streams in the vicinity of New York City (Tran et al. 2010). However, localized extirpations of *Eccopturaxanthenes* have been observed in some streams north of New York City as a result of land use changes (Martin Rosenfeld, Personal Communication, 2009). Isolated patches of intact habitat remain in streams in Central Park including reports of *Agnetinacapitata* by the New York State Department of Environmental Conservation (Robert Bode, Personal Communication, 2008). Several widespread and common species have been collected from the peripheral areas north of New York City including recent records of *Alloperlaatlantica*, *Haploperlabrevis*, *Paragnetinamedia*, *Ostrocercatruncata*, and *Amphinemuranigritta*. Those stoneflies that do remain in these highly urbanized areas are likely pollution or thermally tolerant species that have remained in small fragments of protected habitats.

In many cases, sampling events in streams draining areas dominated by agricultural in the lowlands encircling the Adirondack Park yielded negative collection results of stoneflies. However, many pollution tolerant mayflies (e.g. *Stenonemafemoratum* (Say, 1823)) and caddisflies (e.g. *Cheumatopsycheanalis* (Banks, 1903)) were observed in these areas. When stoneflies were collected, widespread and common species were often documented (e.g. *Allocapniapygmaea and Alloperlaatlantica*). Other unique occurences in these areas included a number of species that appear restricted to lower elevations with distributions extending into low-lying areas of Canada (e.g. *Allocapniagranulata* and *Taeniopteryxmetequi*).

When our field work for this project first began in 2008, there were only four SGCNs in the original 2005 NY WAP (Wellman et al. 2024). The subsequent revision in 2015 (Wellman et al. 2024) also had four SGCNs, except with two from the 2005 WAP and two newly added species). The forthcoming 2025 WAP highlights 18 species of stoneflies as SGCNs (New York State Department of Environmental Conservation 2025). Admittedly, these recommendations are largely opinions and lack formal scientifically-based approaches at assessing rarity (e.g., NatureServe Rank Calculations or other methods using a percent area occupancy approach combined with an accurate assesment of threats). While we currently know more than we once did regarding the distribution of species in the state, our knowledge of specific threats remains difficult to acertain for many of the species of conservation concern in New York.

## Supplementary Material

E47E9E63-908B-5E85-8C38-B27D5BC6E1FC 10.3897/BDJ.13.e158952.suppl1 Supplementary material 1 NY Plecoptera data V1 Data type occurrences Brief description The following dataset presents all of the specimen records included in the paper titled: Distributional and species richness patterns of the stoneflies (Insecta, Plecoptera) in New York State. This paper is published in Biodiversity Data Journal. This dataset combines specimen record data from Colorado State University Insect Collection, Lake Champlain Research Institute, New York State Museum, Illinois Natural History Survey Insect Collection, Cornell University Insect Collection, and several other institutional, personal, and literature records. The data includes specimens to their lowest possible taxonomic rank based on identification and determination. The data includes geo-references for all specimens. This data file will be available for download from Global Biodiversity Information Facility (Myers L, Kondratieff B, Grubbs S, Pett L, DeWalt R E, Mihuc T, Hart L (2025). Distributional and species richness patterns of the stoneflies (Insecta, Plecoptera) in New York State: occurrence dataset. Version 1.1. Biodiversity Data Journal. Occurrence dataset https://doi.org/10.15468/hkum7k accessed via GBIF.org on 2025-08-15.). This data file contains a total of 6553 records, structured in 62 columns of data in DwCA format.
Below is a list of the column labels and their descriptions:
**basisOfRecord**: The specific nature of the data record.
**ownerInstitutionCode**: The name (or acronym) in use by the institution having ownership of the object(s) or information referred to in the record.
**collectionCode**: The name, acronym, coden, or initialism identifying the collection or data set from which the record was derived.
**catalogNumbe**r: An identifier (preferably unique) for the record within the data set or collection.
**bibliographicCitation**: Reference of publication in which the MaterialCitation was found.
**order**: The full scientific name of the order in which the dwc:Taxon is classified.
**family**: The full scientific name of the family in which the dwc:Taxon is classified.
**genus**: The full scientific name of the genus in which the dwc:Taxon is classified.
**specificEpithet**: The name of the first or species epithet of the dwc:scientificName.
**scientificName**: The full scientific name, with authorship and date information if known. When forming part of a dwc:Identification, this should be the name in lowest level taxonomic rank that can be determined.
**scientificNameAuthorship**: The authorship information for the dwc:scientificName formatted according to the conventions of the applicable dwc:nomenclaturalCode.
**identificationQualifier**: A brief phrase or a standard term ("cf.", "aff.") to express the determiner's doubts about the dwc:Identification.
**typeStatus**: A list (concatenated and separated) of nomenclatural types (type status, typified scientific name, publication) applied to the subject.
**adultFemale**: Custom field indicating the total count of adult females for this record.
**adultMale**: Custom field indicating the total count of adult males for this record.
**adultUnsexed**: Custom field indicating the total count of adults (sex unknown) for this record.
**ageUnknown**: Custom field indicating the total count of individuals (age unknown) for this record.
**immature**: Custom field indicating the total count of immatures for this record.
**exuvium**: Custom field indicating the total count of exuvia for this record.
**individualCount**: The number of individuals present at the time of the dwc:Occurrence.
**lifeStage**: The age class or life stage of the dwc:Organism(s) at the time the dwc:Occurrence was recorded.
**sex**: The sex of the biological individual(s) represented in the dwc:Occurrence.
**identifiedBy**: A list (concatenated and separated) of names of people, groups, or organizations who assigned the dwc:Taxon to the subject.
**identifiedByID**: A list (concatenated and separated) of the globally unique identifier for the person, people, groups, or organizations responsible for assigning the dwc:Taxon to the subject.
**dateIdentified**: The date on which the subject was determined as representing the dwc:Taxon.
**verbatimIdentification**: A string representing the taxonomic identification as it appeared in the original record.
**identificationRemarks**: Comments or notes about the dwc:Identification.
**occurrenceRemarks**: Comments or notes about the dwc:Occurrence.
**verbatimOtherLabels**: Custom field for verbatim labels associated with the record that do not include verbatimLocality or verbatimIdentification.
**countryCode**: The standard code for the country in which the dcterms:Location occurs.
**stateProvince**: The name of the next smaller administrative region than country (state, province, canton, department, region, etc.) in which the dcterms:Location occurs.
**county**: The full, unabbreviated name of the next smaller administrative region than stateProvince (county, shire, department, etc.) in which the dcterms:Location occurs.
**locality**: The specific description of the place.
**verbatimLocality**: The original textual description of the place.
**eventDate**: The date-time or interval during which a dwc:Event occurred. For occurrences, this is the date-time when the dwc:Event was recorded. Not suitable for a time in a geological context.
**waterBody**: The name of the water body in which the dcterms:Location occurs.
**habitat**: A category or description of the habitat in which the dwc:Event occurred.
**samplingProtocol**: The methods or protocols used during a dwc:Event, denoted by an IRI.
**emergedDate**: Custom field indicating the date of emergence of reared insect specimen records.
**fieldNumber**: An identifier given to the dwc:Event in the field. Often serves as a link between field notes and the dwc:Event.
**eventRemarks**: Comments or notes about the dwc:Event.
**recordedBy**: A person, group, or organization responsible for recording the original dwc:Occurrence.
**recordedByID**: A list (concatenated and separated) of the globally unique identifier for the person, people, groups, or organizations responsible for recording the original dwc:Occurrence.
**decimalLatitude**: The geographic latitude (in decimal degrees, using the spatial reference system given in dwc:geodeticDatum) of the geographic center of a dcterms:Location. Positive values are north of the Equator, negative values are south of it. Legal values lie between -90 and 90, inclusive.
**decimalLongitude**: The geographic longitude (in decimal degrees, using the spatial reference system given in dwc:geodeticDatum) of the geographic center of a dcterms:Location. Positive values are east of the Greenwich Meridian, negative values are west of it. Legal values lie between -180 and 180, inclusive.
**verbatimCoordinates**: The verbatim original spatial coordinates of the dcterms:Location. The coordinate ellipsoid, geodeticDatum, or full Spatial Reference System (SRS) for these coordinates should be stored in dwc:verbatimSRS and the coordinate system should be stored in dwc:verbatimCoordinateSystem.
**coordinatePrecisionCode**: Custom field indicating the coordinate precision code for each collecting event, where 1=10m, 2=1000m, 3=10,0000m, 4=100,000m.
**coordinateUncertaintyInMeters**: The horizontal distance (in meters) from the given dwc:decimalLatitude and dwc:decimalLongitude describing the smallest circle containing the whole of the dcterms:Location. Leave the value empty if the uncertainty is unknown, cannot be estimated, or is not applicable (because there are no coordinates). Zero is not a valid value for this term.
**verbatimElevation**: The original description of the elevation (altitude, usually above sea level) of the Location.
**elevationBand**: Custom field indicating the minimum and maximum elevation in meters of the collecting event.
**minimumElevationInMeters**: The lower limit of the range of elevation (altitude, usually above sea level), in meters.
**maximumElevationInMeters**: The upper limit of the range of elevation (altitude, usually above sea level), in meters.
**huc6**: Custom field indicating the USGS HUC6 identification number for the location of the collecting event.
**huc6_name**: Custom field indicating the USGS HUC6 name for the location of the collecting event.
**huc8**: Custom field indicating the USGS HUC8 identification number for the location of the collecting event.
**huc8_name**: Custom field indicating the USGS HUC8 name for the location of the collecting event.
**US_L4CODE**: Custom field indicating the USEPA Level IV Ecoregion Code for the location of the collecting event.
**US_L4NAME**: Custom field indicating the USEPA Level IV Ecoregion Name for the location of the collecting event.
**US_L3CODE**: Custom field indicating the USEPA Leve III Ecoregion Code for the location of the collecting event.
**US_L3NAME**: Custom field indicating the USEPA Level III Ecoregion Name for the location of the collecting event.
**NA_L2CODE**: Custom field indicating the North America Level II Ecoregion Code for the location of the collecting event.
**NA_L2NAME**: Custom field indicating the North America Level II Ecoregion Name for the location of the collecting event. File: oo_1322922.tsv https://binary.pensoft.net/file/1322922 Luke W. Myers, Boris C. Kondratieff, Scott A. Grubbs, Lindsey A. Pett, R. Edward DeWalt, Timothy B. Mihuc, Lily V. Hart

## Figures and Tables

**Figure 10a.**
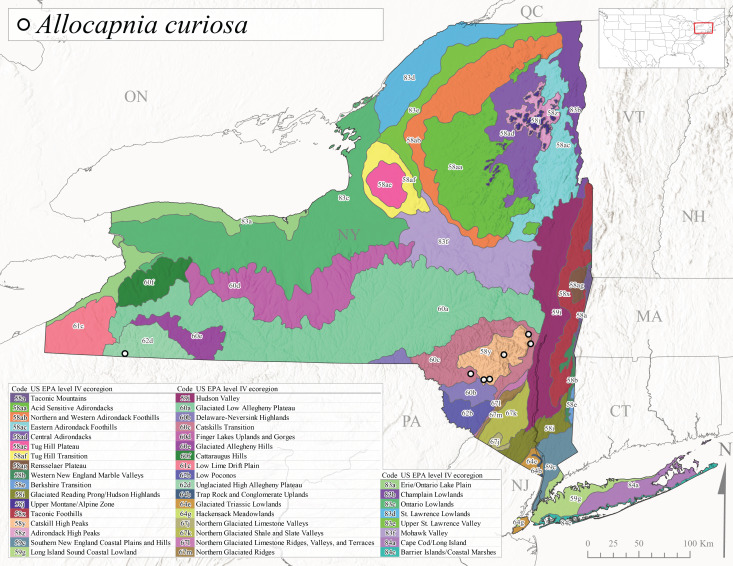
Allocapniacuriosa

**Figure 10b.**
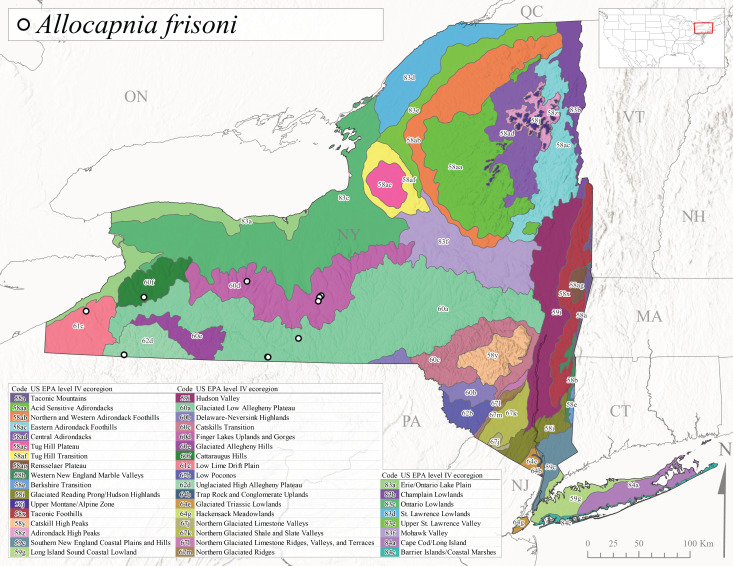
Allocapniafrisoni

**Figure 10c.**
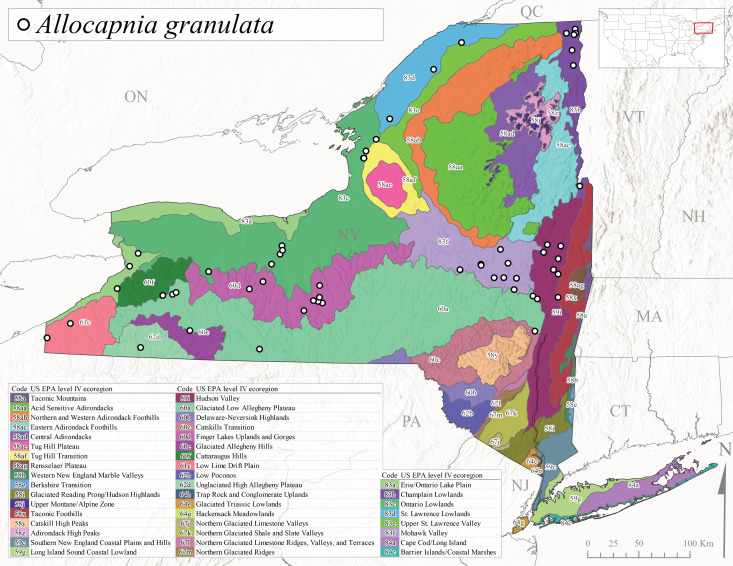
Allocapniagranulata

**Figure 10d.**
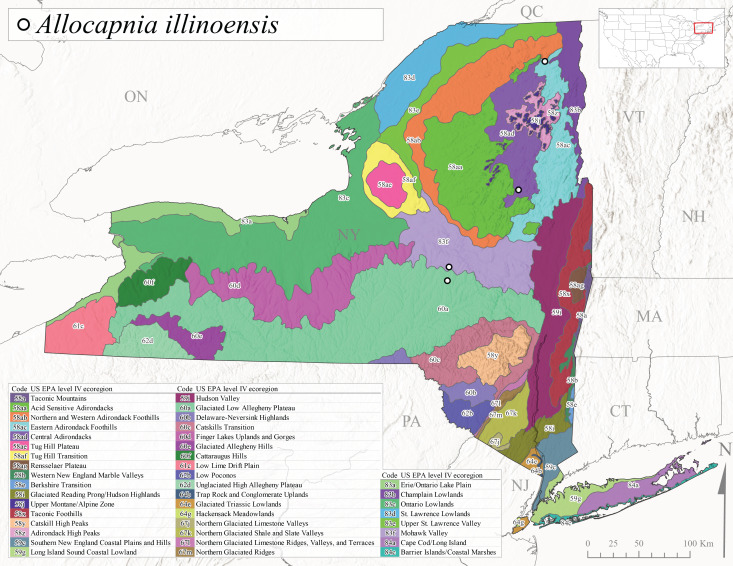
Allocapniaillinoensis

**Figure 10e.**
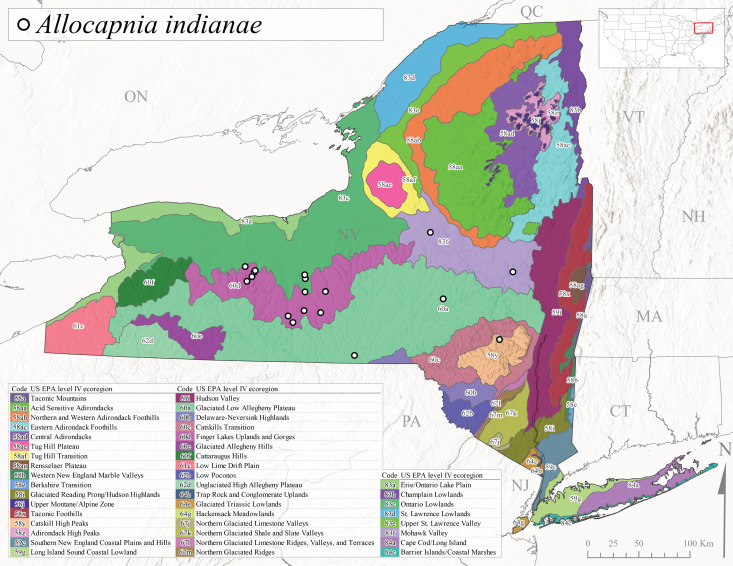
Allocapniaindianae

**Figure 10f.**
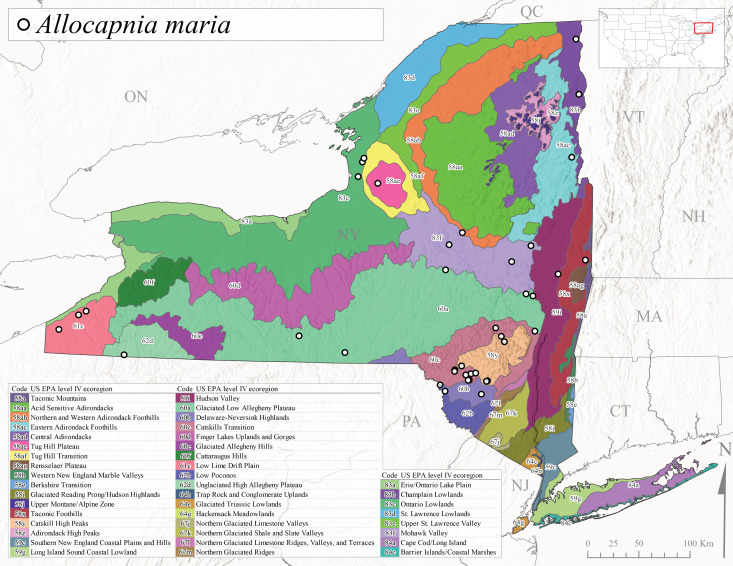
Allocapniamaria

**Figure 11a.**
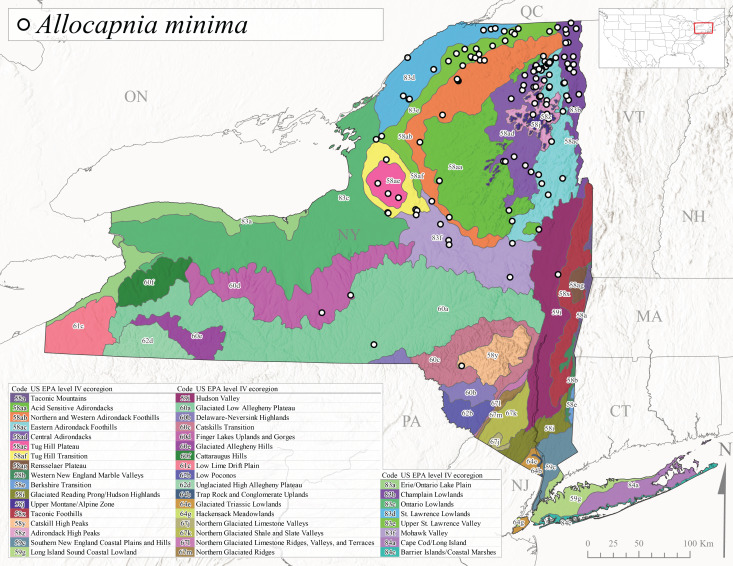
Allocapniaminima

**Figure 11b.**
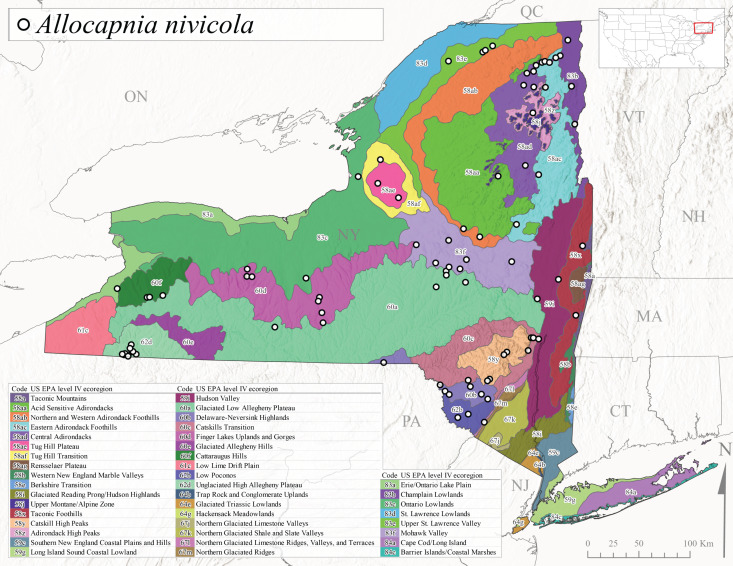
Allocapnianivicola

**Figure 11c.**
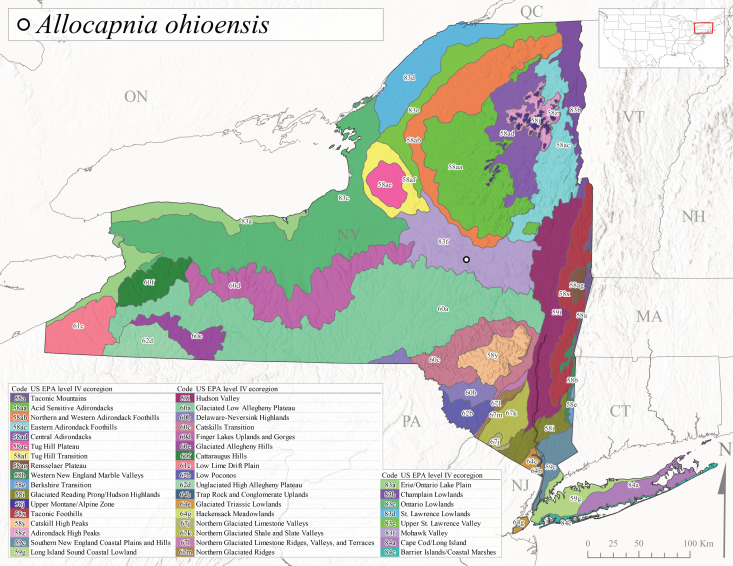
Allocapniaohioensis

**Figure 11d.**
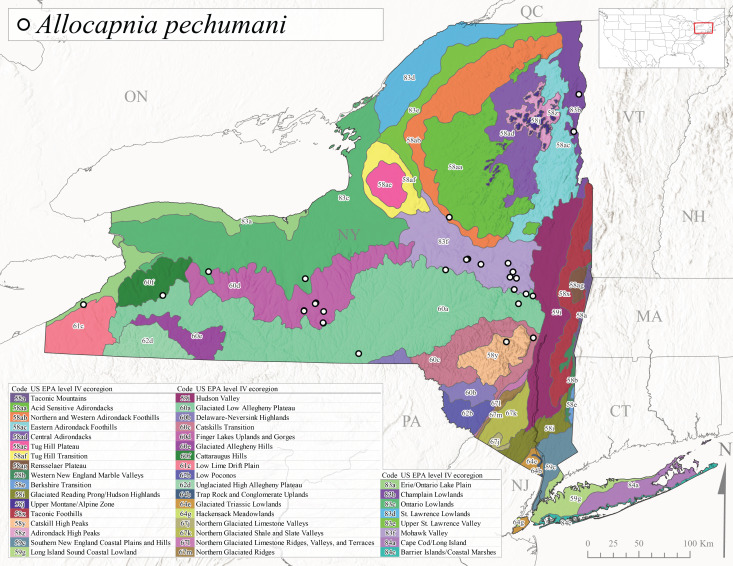
llocapnia pechumani

**Figure 11e.**
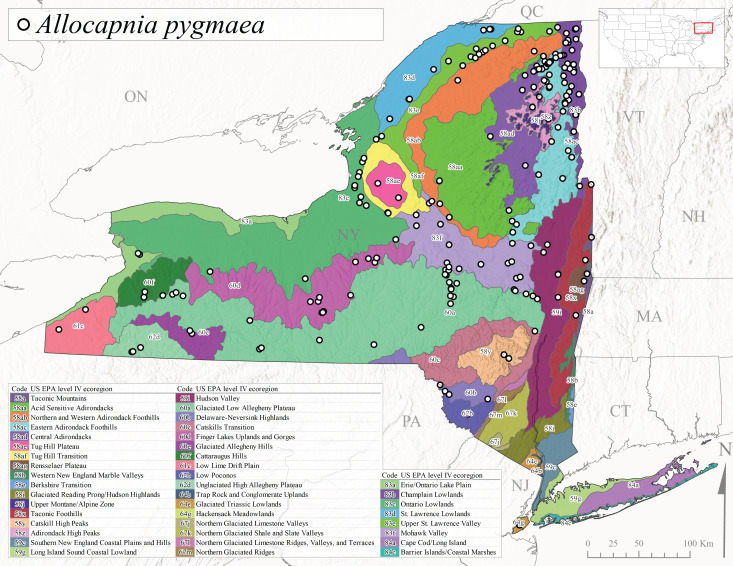
Allocapniapygmaea

**Figure 11f.**
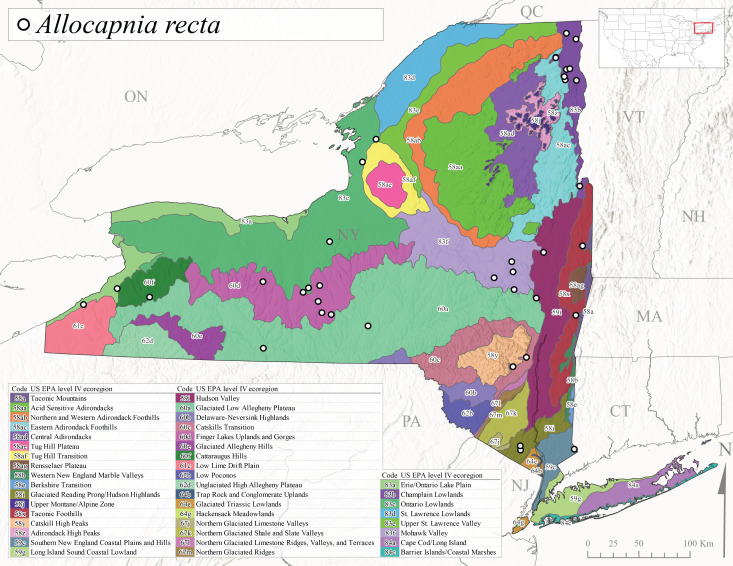
Allocapniarecta

**Figure 12a.**
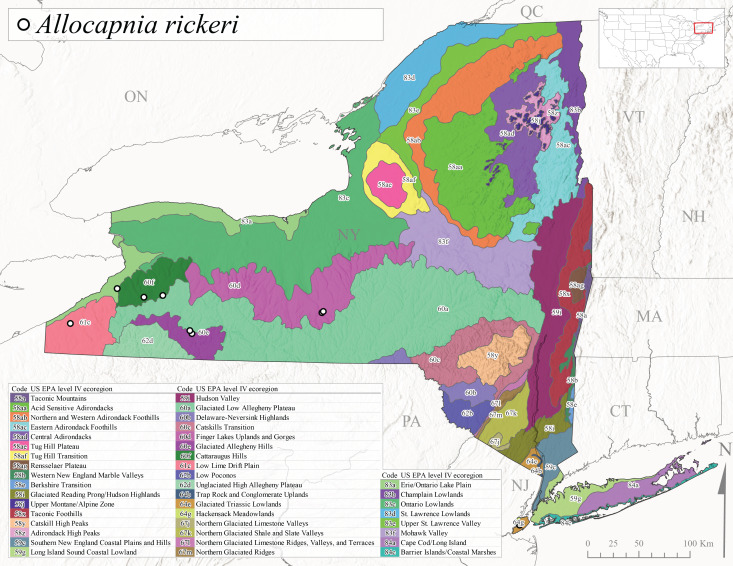
Allocapniarickeri

**Figure 12b.**
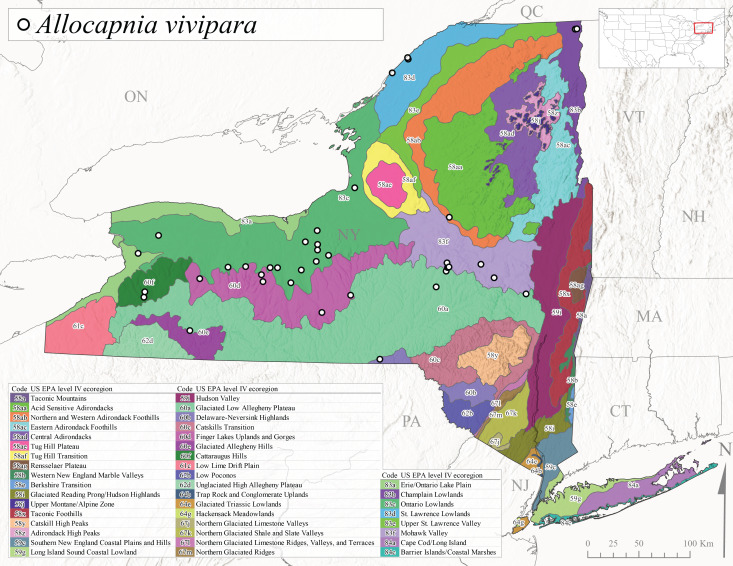
Allocapniavivipara

**Figure 12c.**
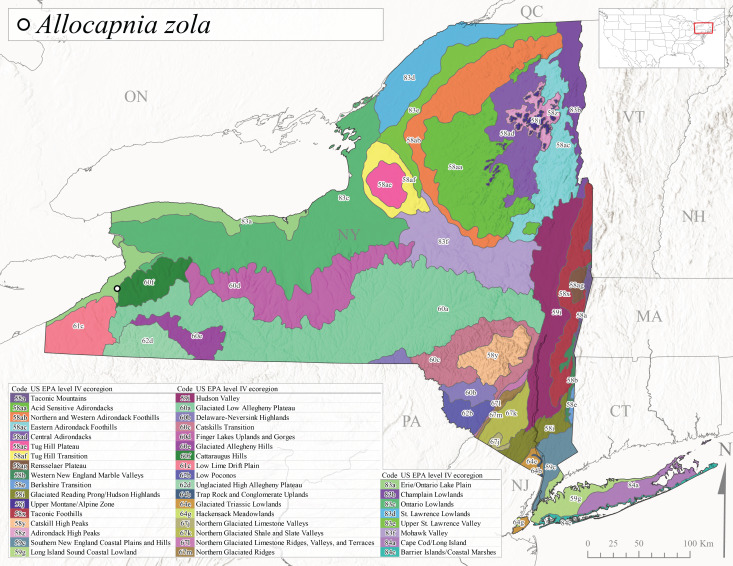
Allocapniazola

**Figure 12d.**
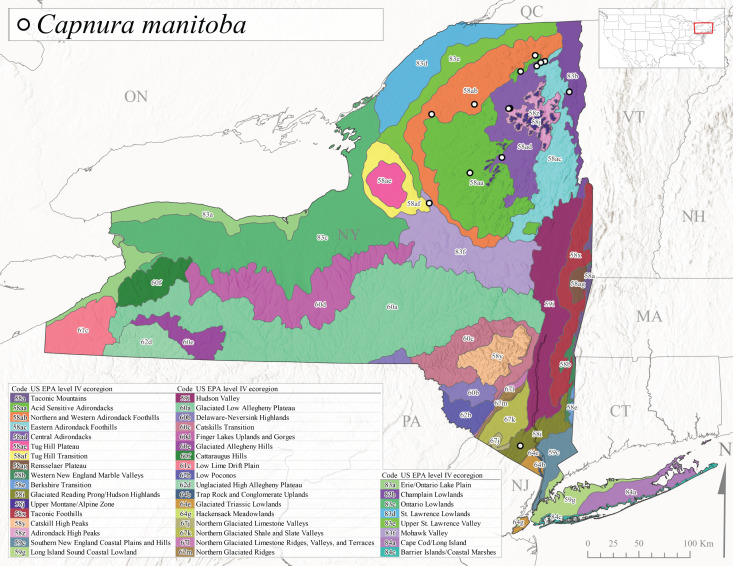
Capnuramanitoba

**Figure 12e.**
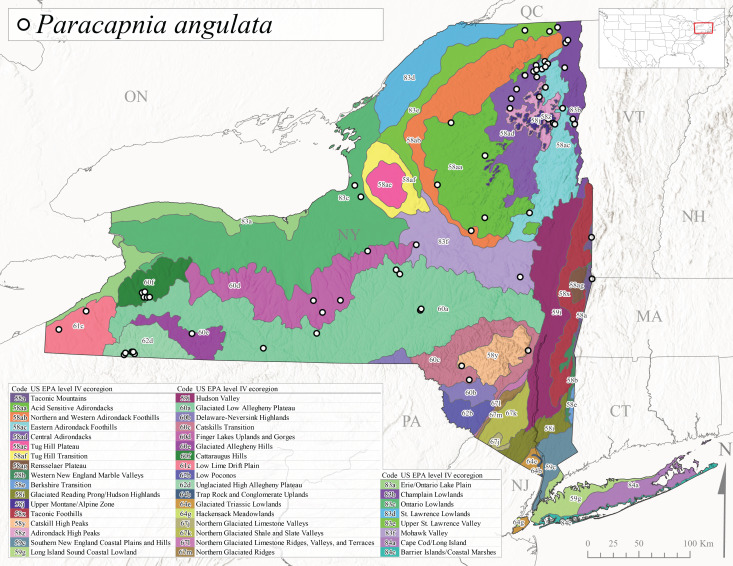
Paracapniaangulata

**Figure 12f.**
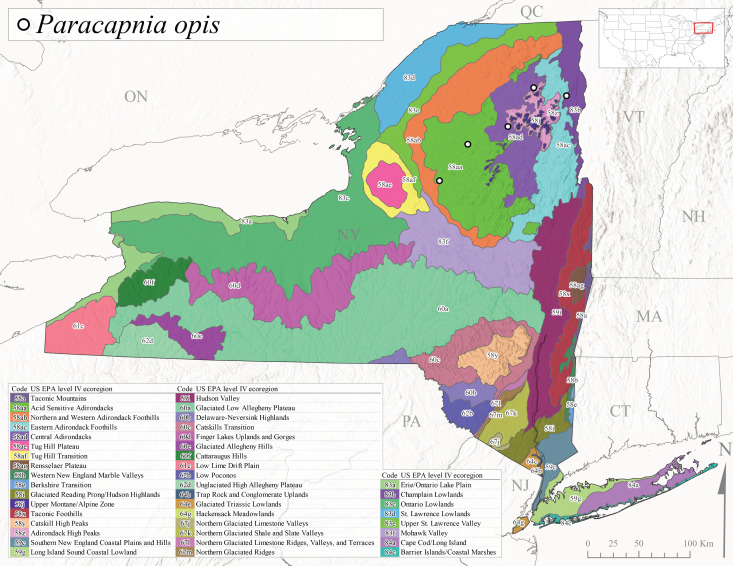
Paracapniaopis

**Figure 15a.**
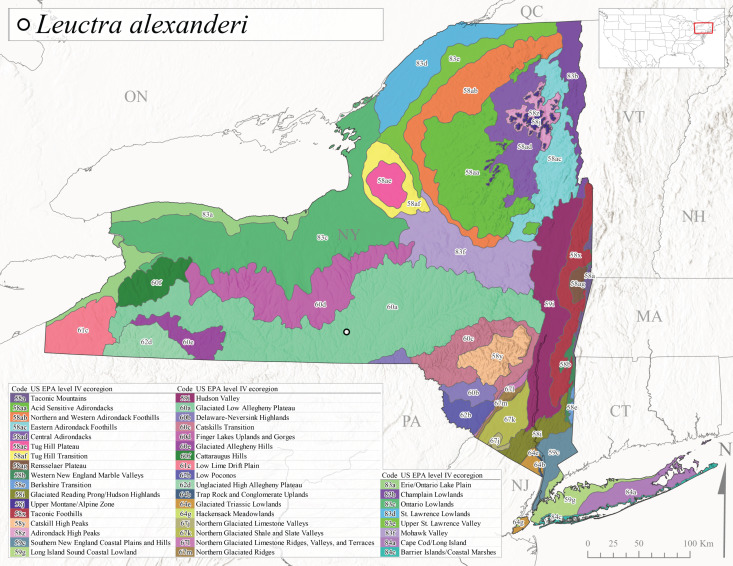
Leuctraalexanderi

**Figure 15b.**
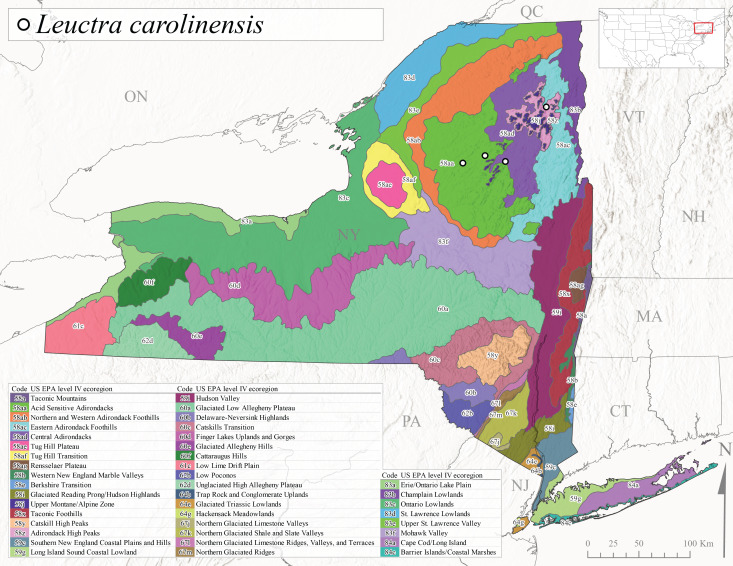
Leuctracarolinensis

**Figure 15c.**
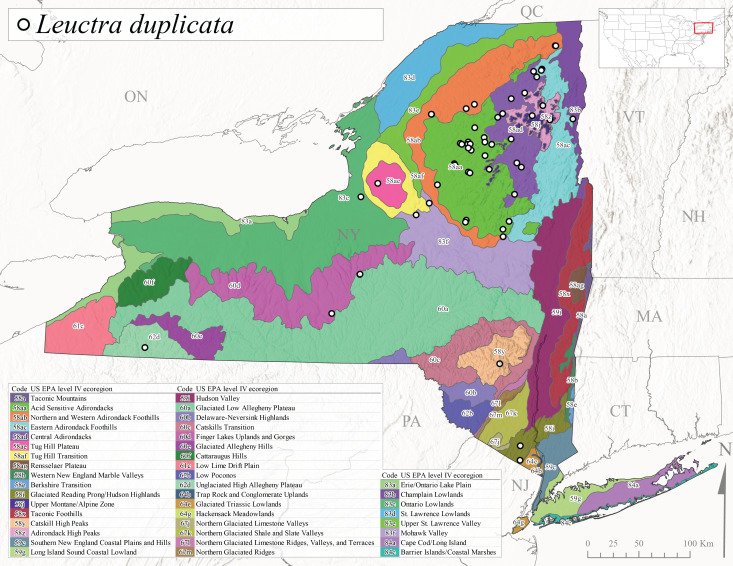
Leuctraduplicata

**Figure 15d.**
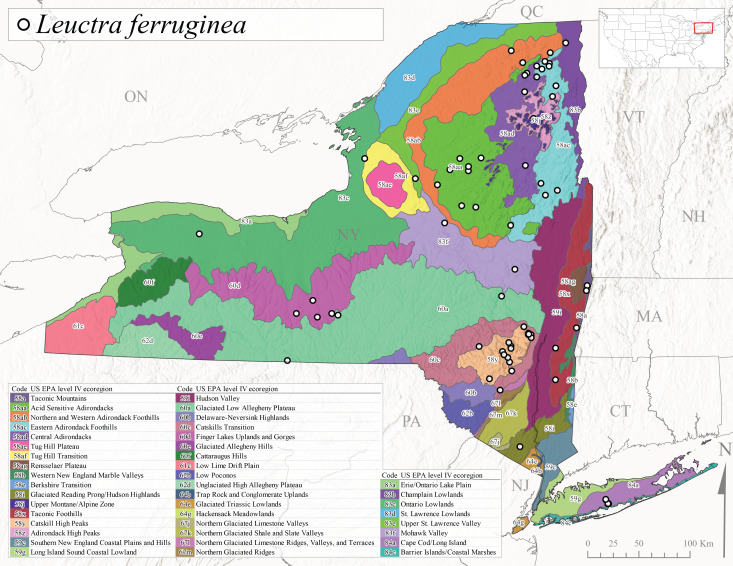
Leuctraferruginea

**Figure 15e.**
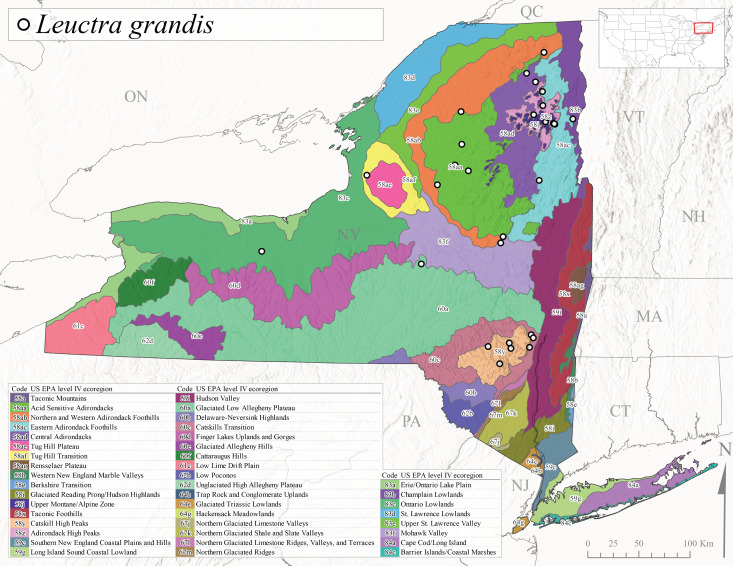
Leuctragrandis

**Figure 15f.**
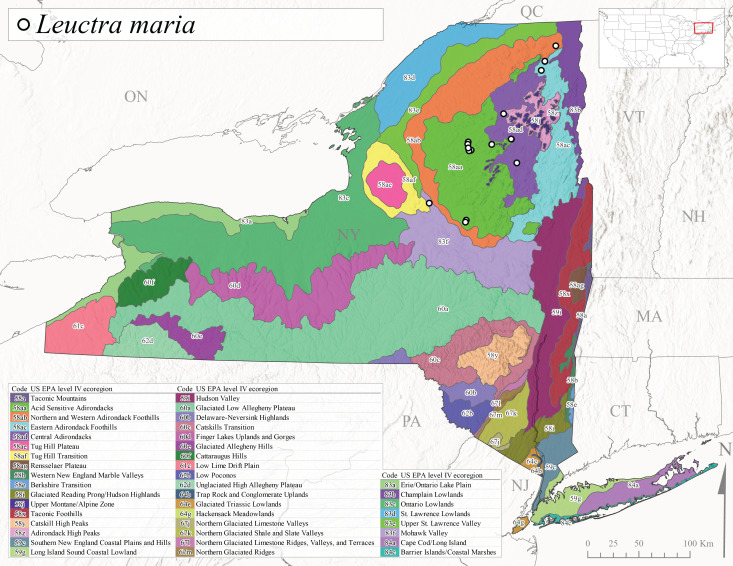
Leuctramaria

**Figure 16a.**
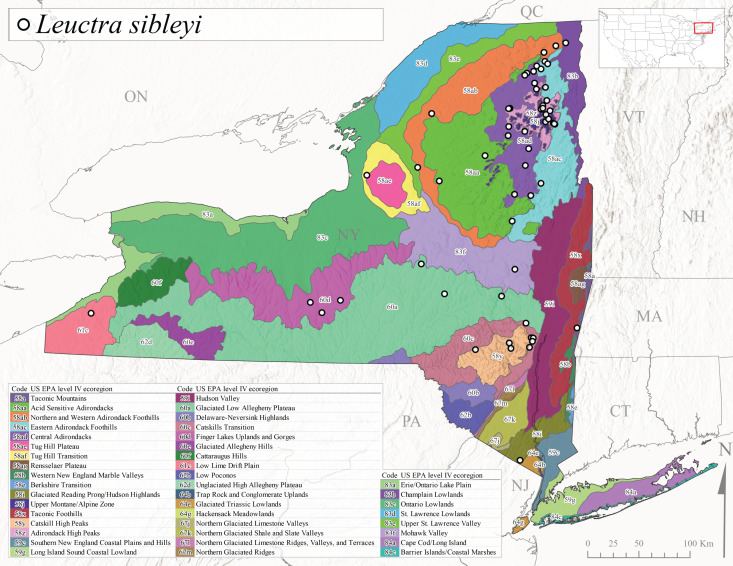
Leuctrasibleyi

**Figure 16b.**
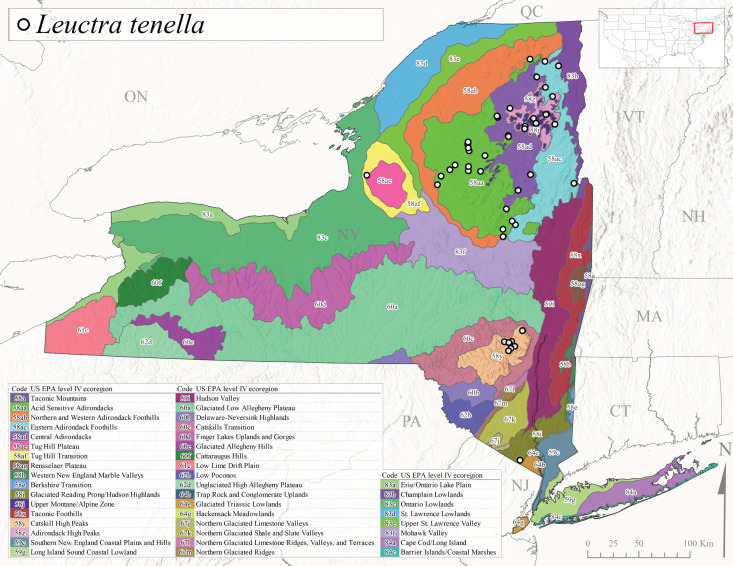
Leuctratenella

**Figure 16c.**
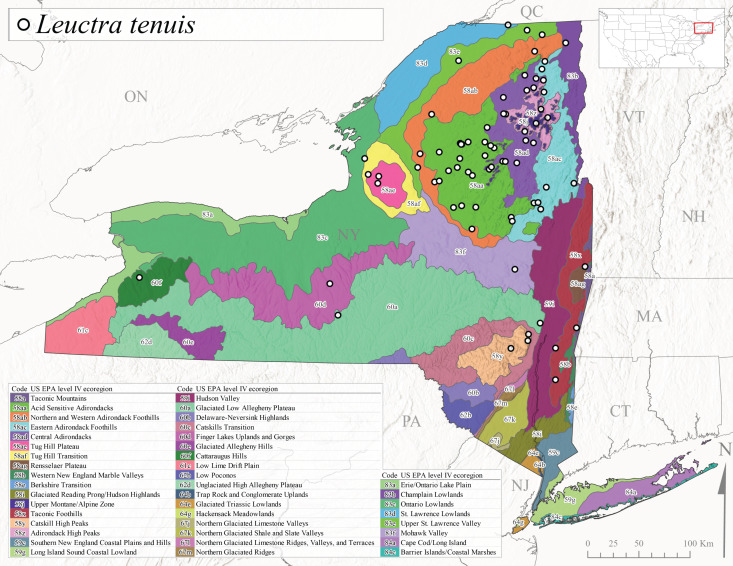
Leuctratenuis

**Figure 16d.**
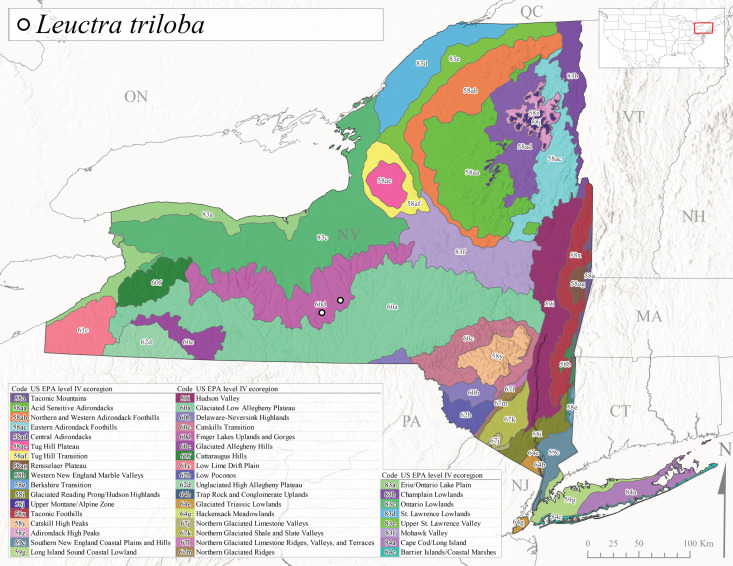
Leuctratriloba

**Figure 16e.**
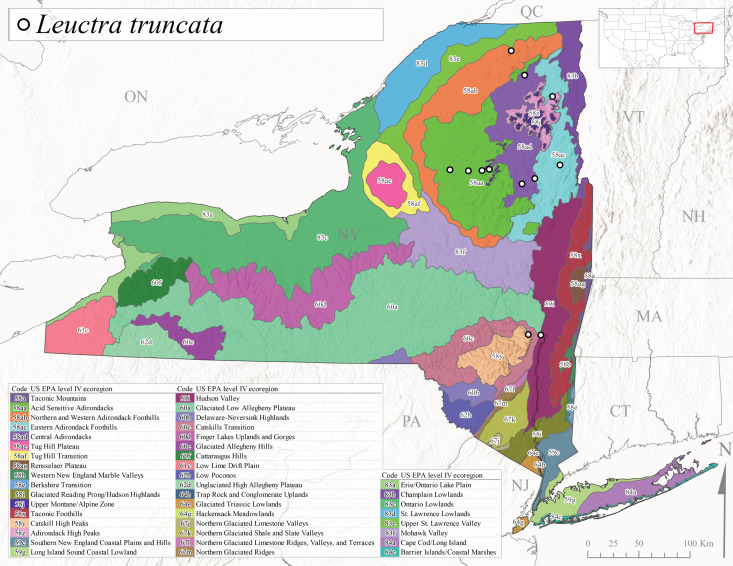
Leuctratruncata

**Figure 16f.**
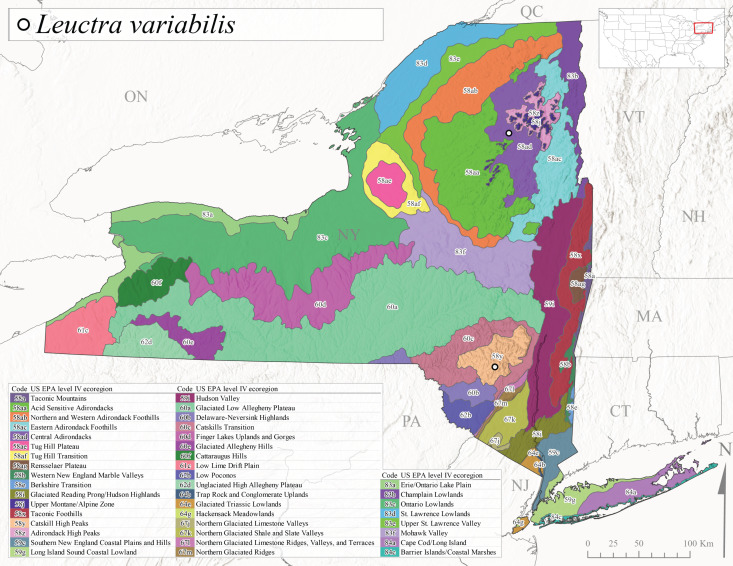
Leuctravariabilis

**Figure 17a.**
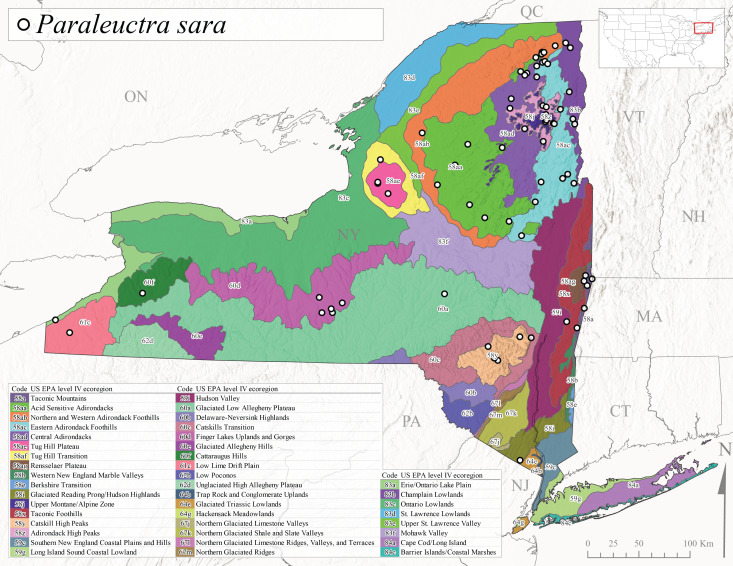
Paraleuctrasara

**Figure 17b.**
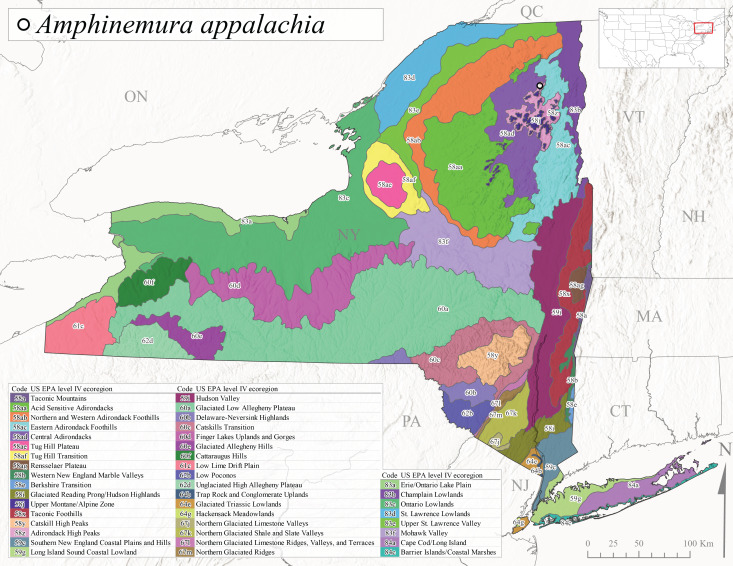
Amphinemuraappalachia

**Figure 17c.**
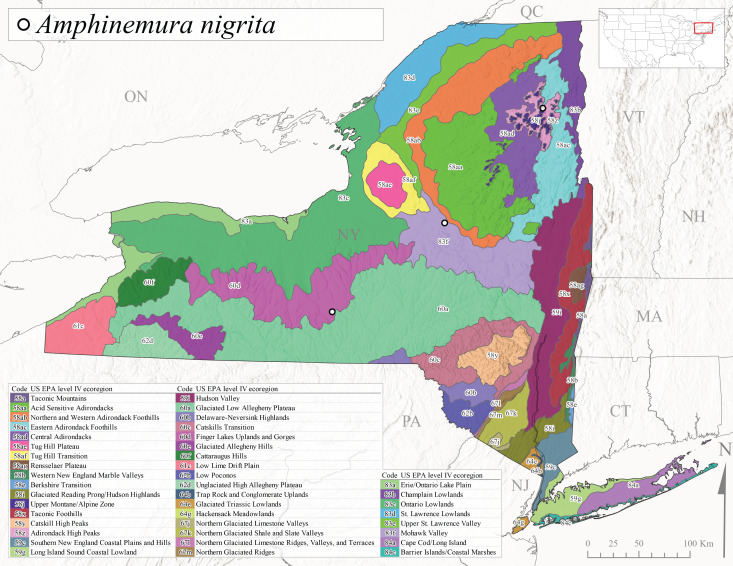
Amphinemuranigritta

**Figure 17d.**
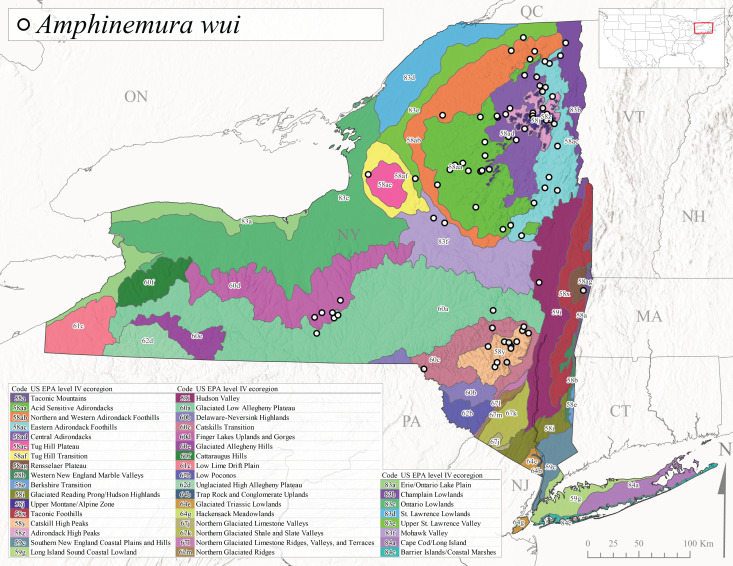
Amphinemurawui

**Figure 17e.**
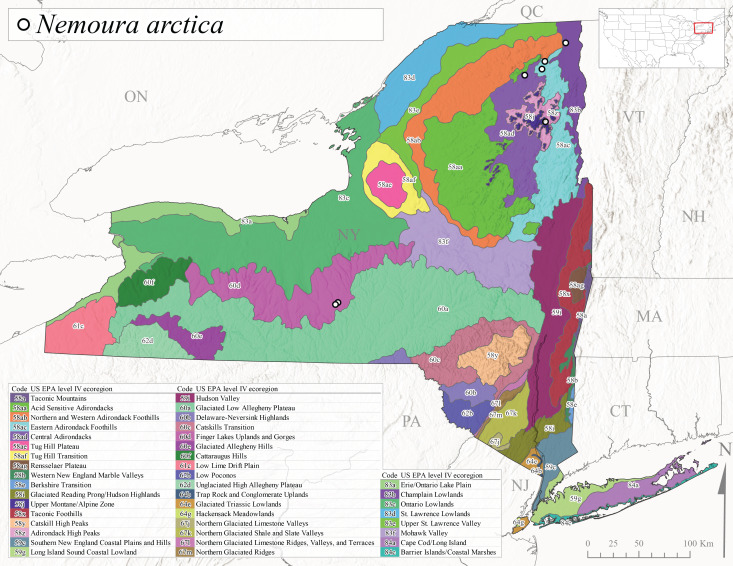
Nemouraarctica

**Figure 17f.**
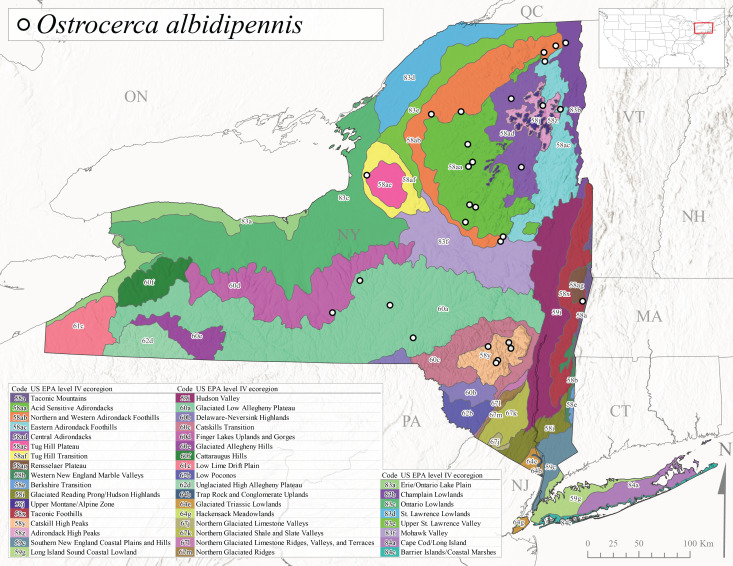
Ostrocercaalbidipennis

**Figure 20a.**
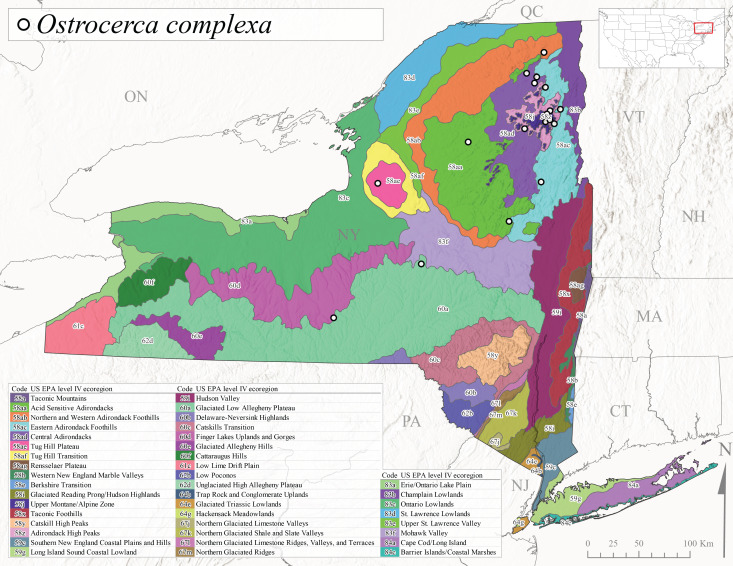
Ostrocercacomplexa

**Figure 20b.**
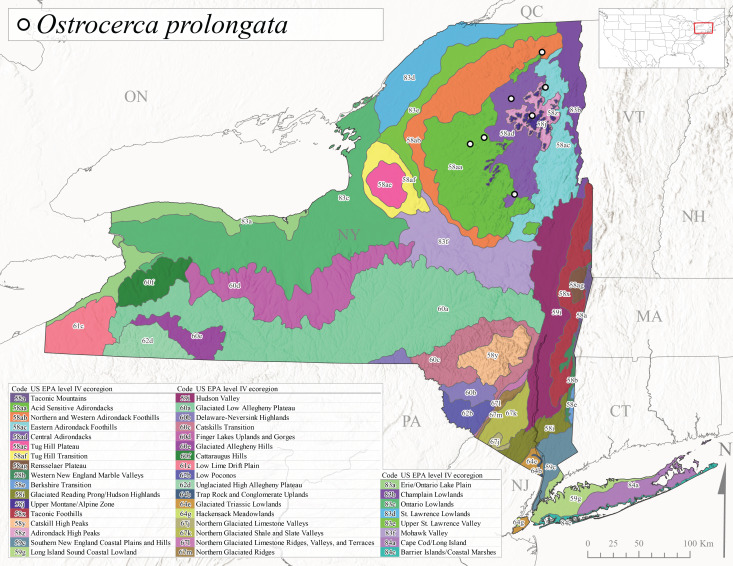
Ostrocercaprolongata

**Figure 20c.**
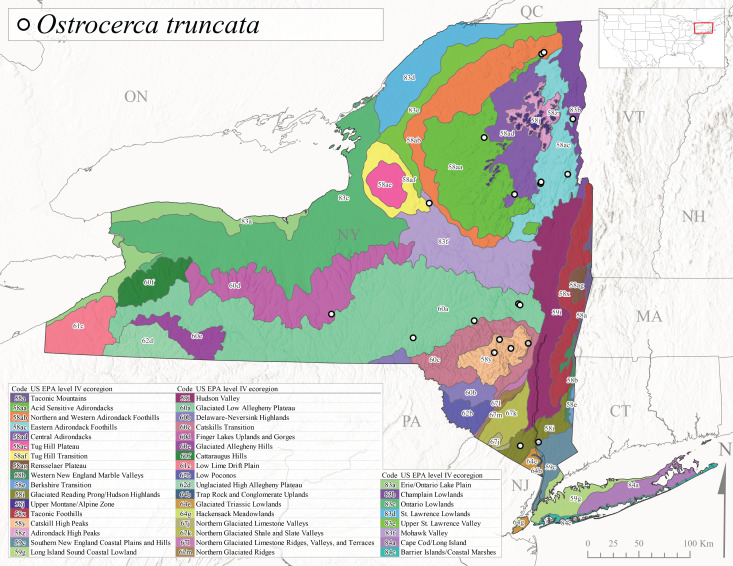
Ostrocercatruncata

**Figure 20d.**
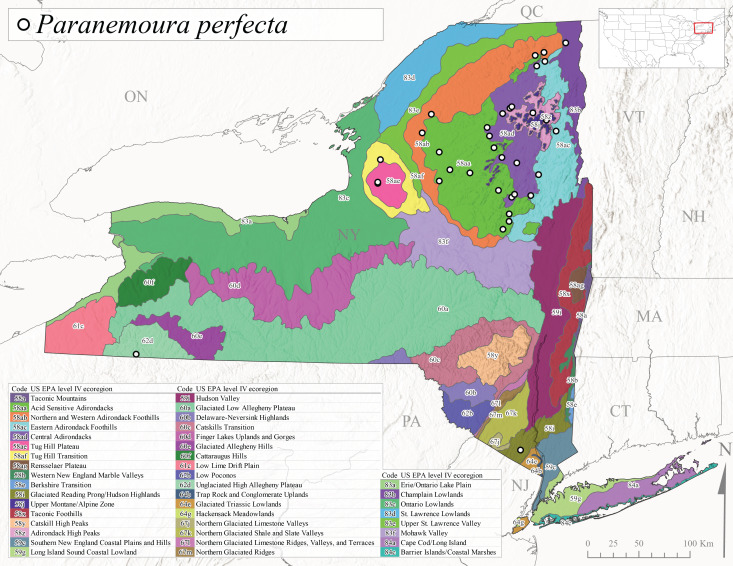
Paranemouraperfecta

**Figure 20e.**
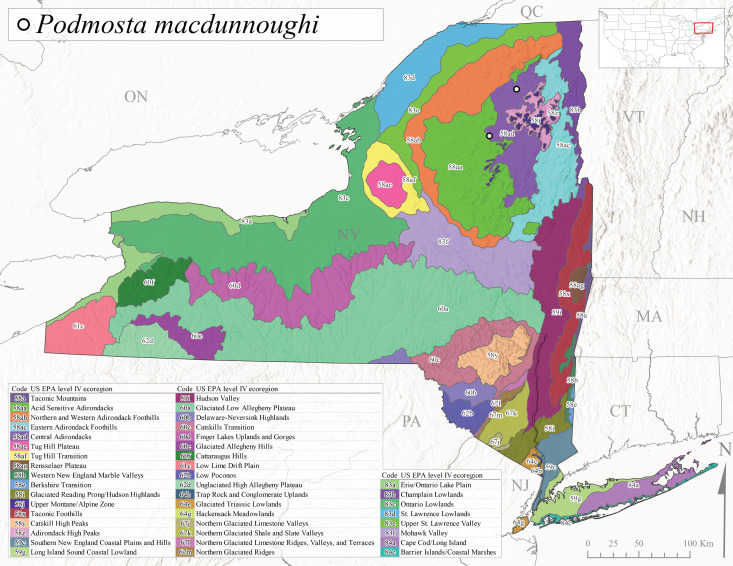
Podmostamacdunnoughi

**Figure 20f.**
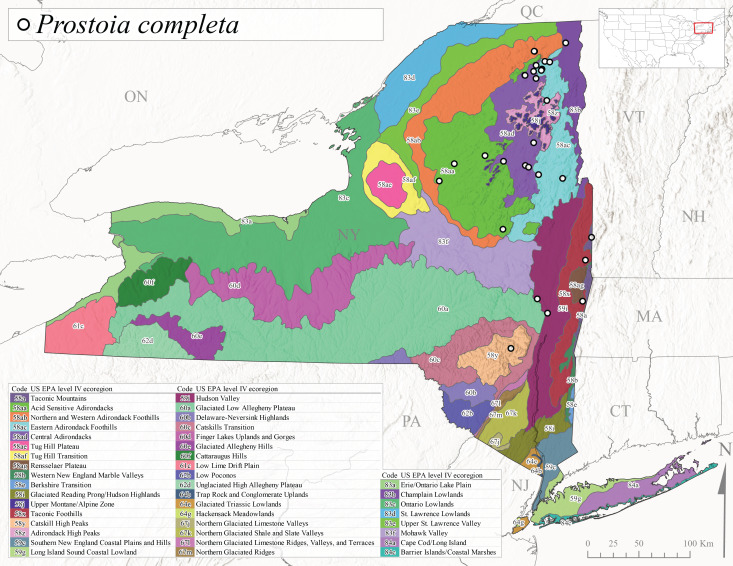
Prostoiacompleta

**Figure 21a.**
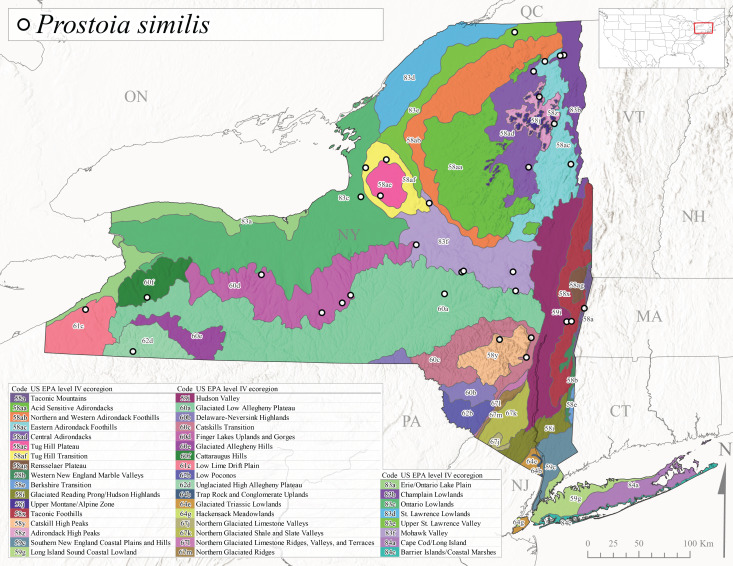
Prostoiasimilis

**Figure 21b.**
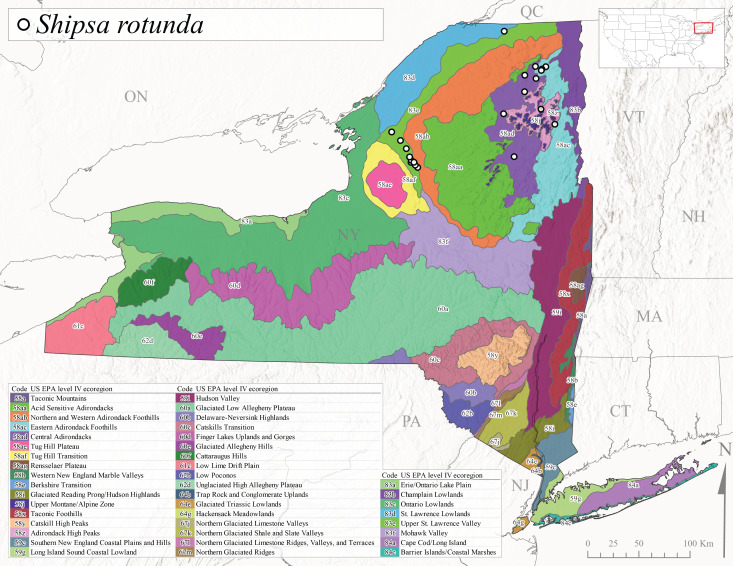
Shipsarotunda

**Figure 21c.**
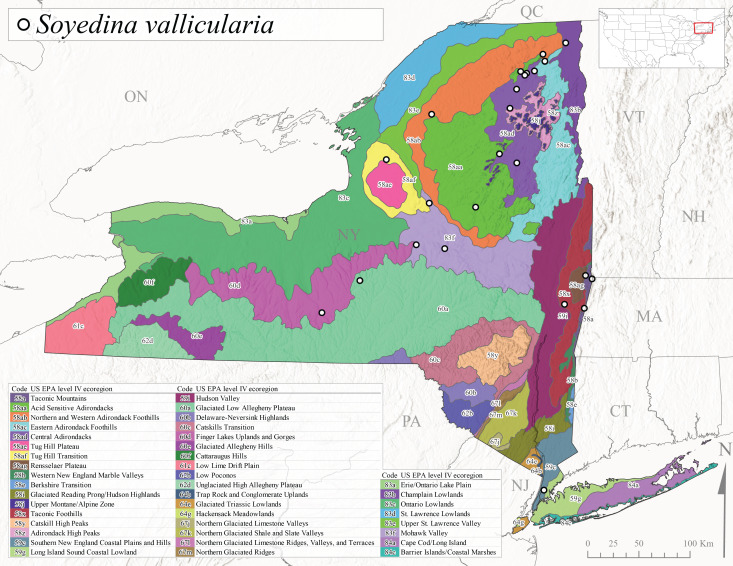
Soyedinavallicularia

**Figure 21d.**
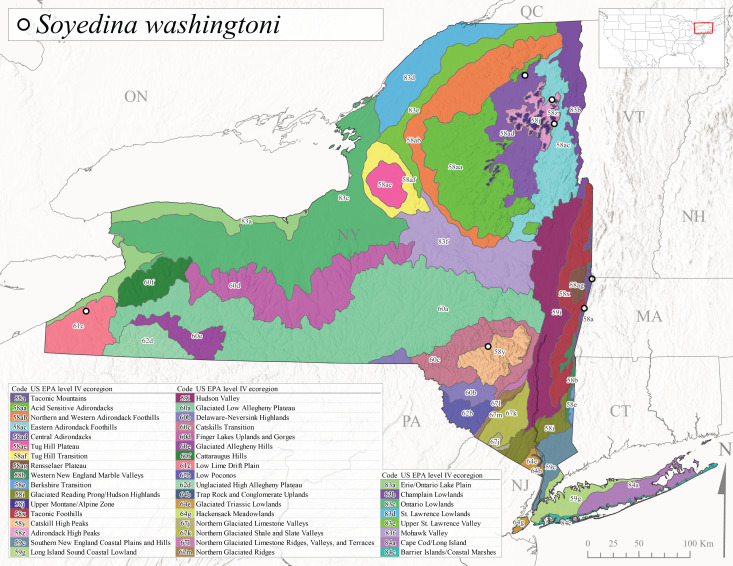
Soyedinawashingtoni

**Figure 21e.**
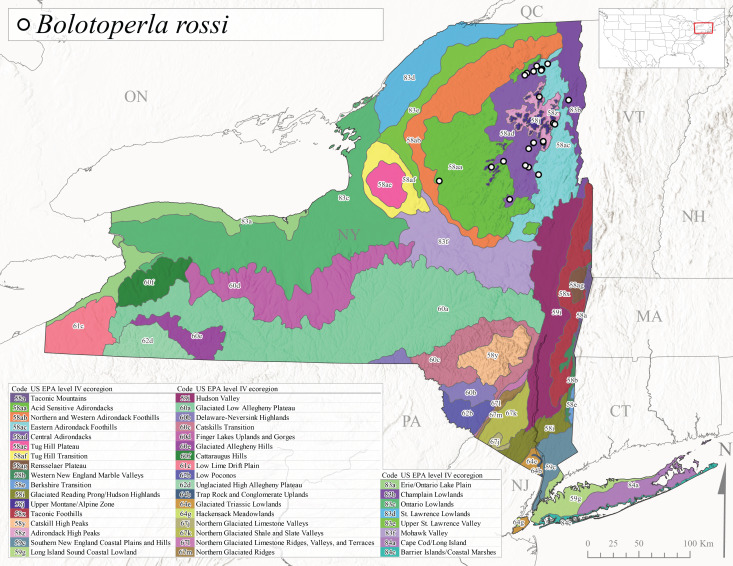
Bolotoperlarossi

**Figure 21f.**
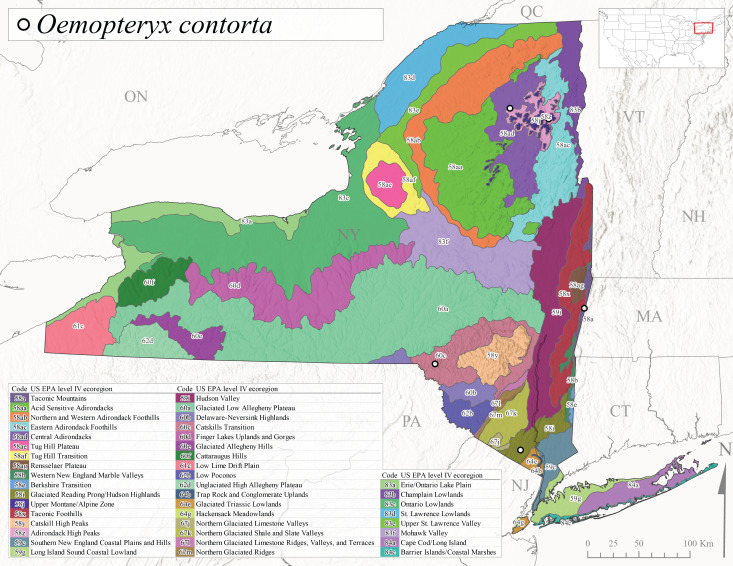
Oemopteryxcontorta

**Figure 24a.**
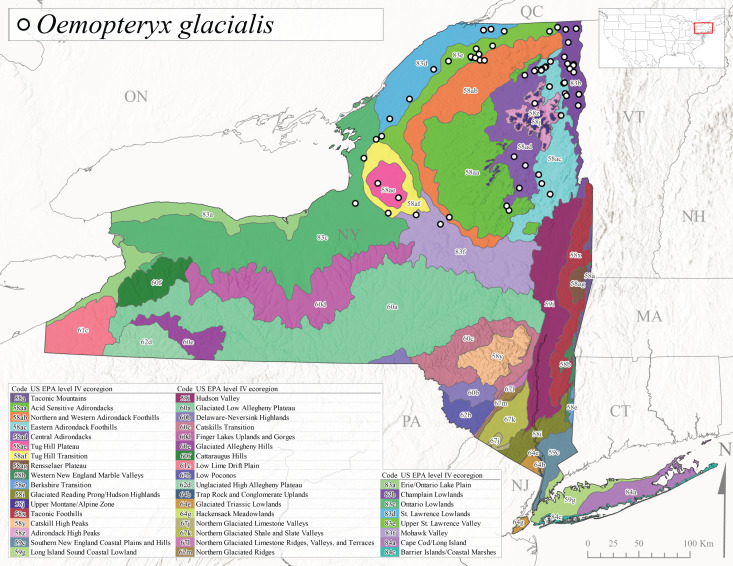
Oemopteryxglacialis

**Figure 24b.**
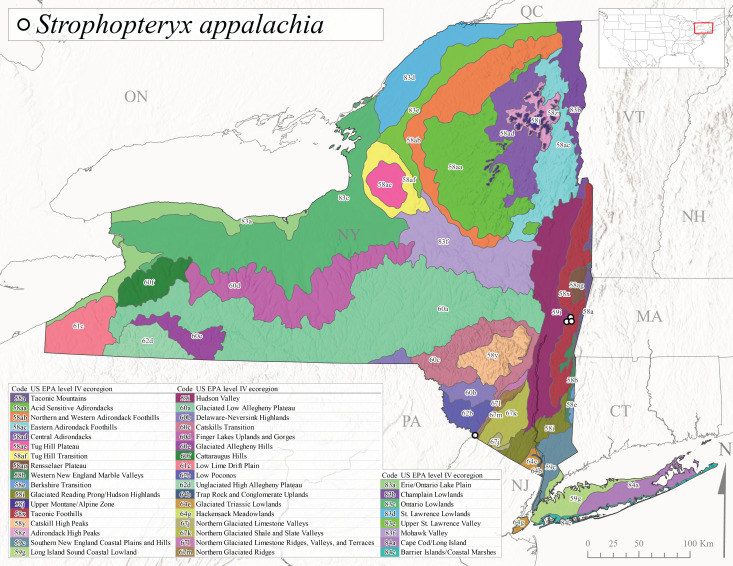
Strophopteryxappalachia

**Figure 24c.**
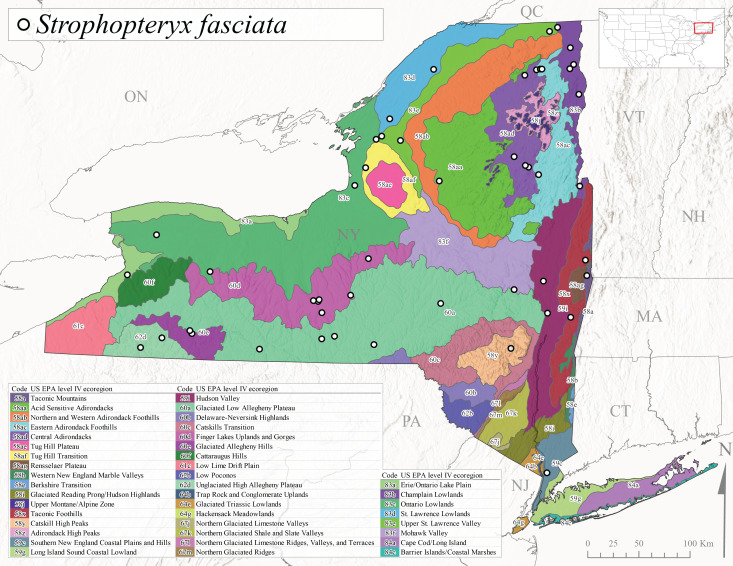
Strophopteryxfasciata

**Figure 24d.**
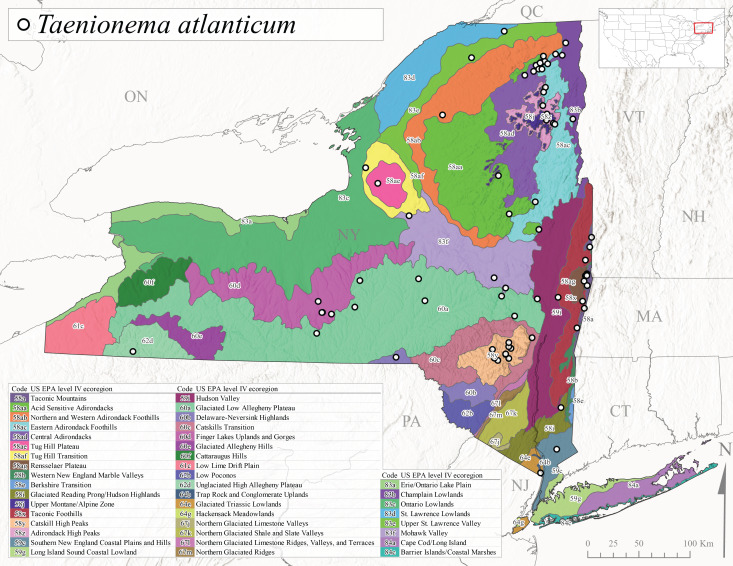
Taenionemaatlanticum

**Figure 24e.**
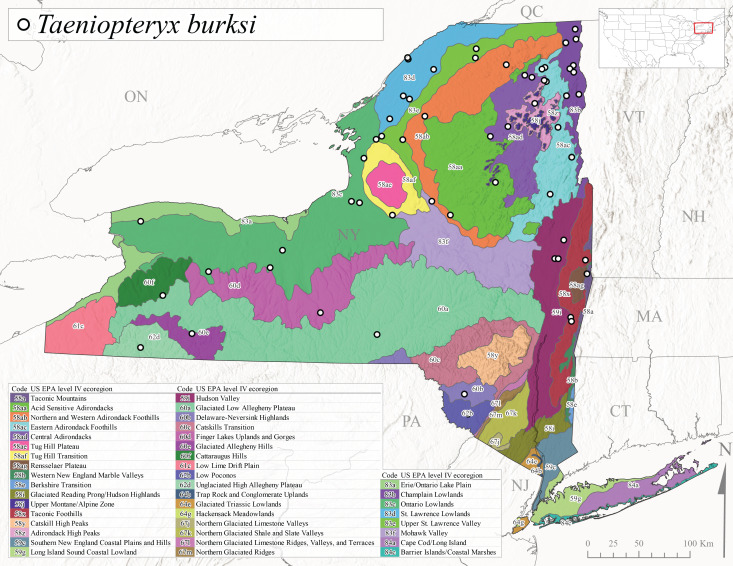
Taeniopteryxburksi

**Figure 24f.**
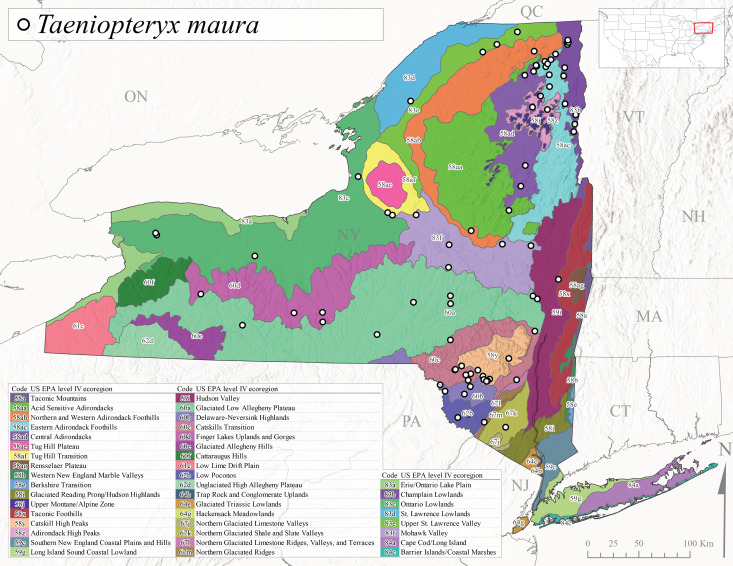
Taeniopteryxmaura

**Figure 25a.**
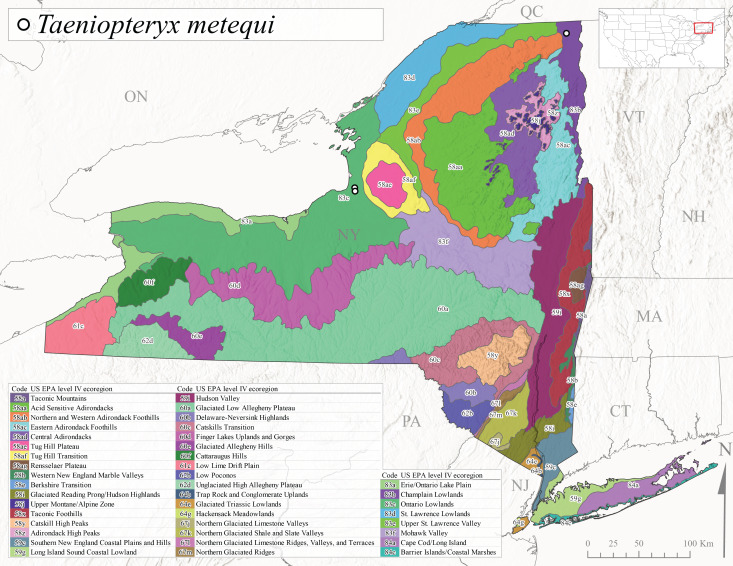
Taeniopteryxmetequi

**Figure 25b.**
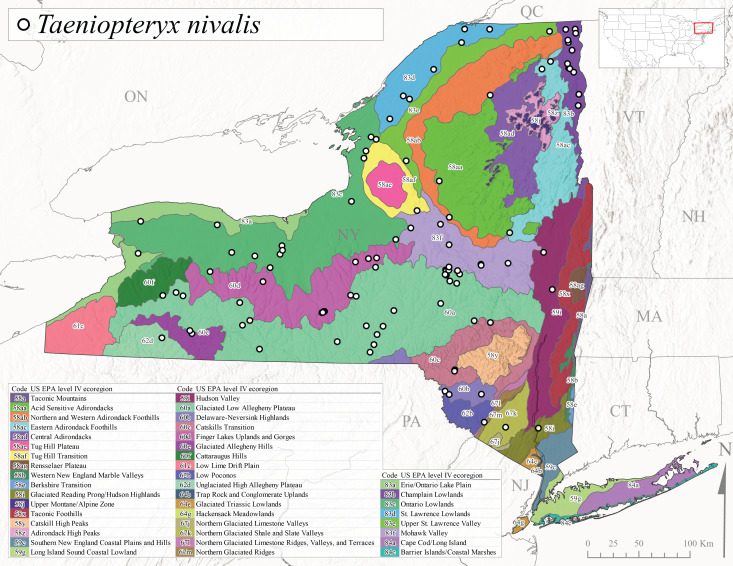
Taeniopteryxnivalis

**Figure 25c.**
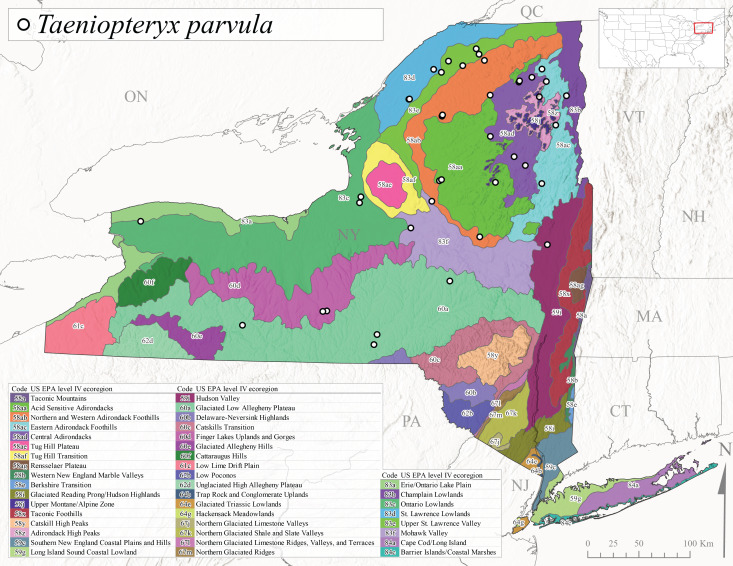
Taeniopteryxparvula

**Figure 25d.**
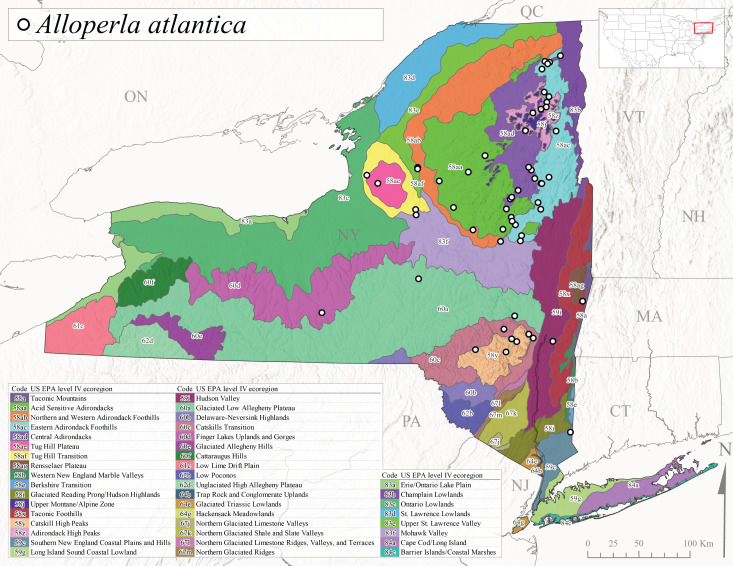
Alloperlaatlantica

**Figure 25e.**
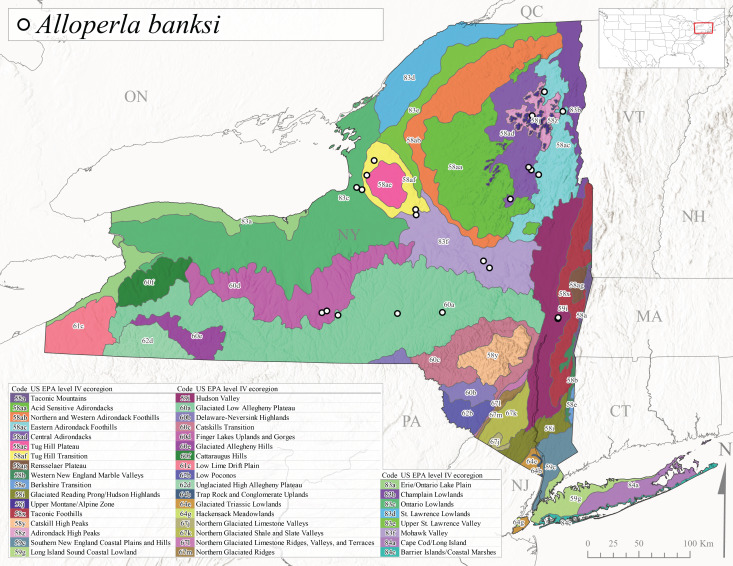
Alloperlabanksi

**Figure 25f.**
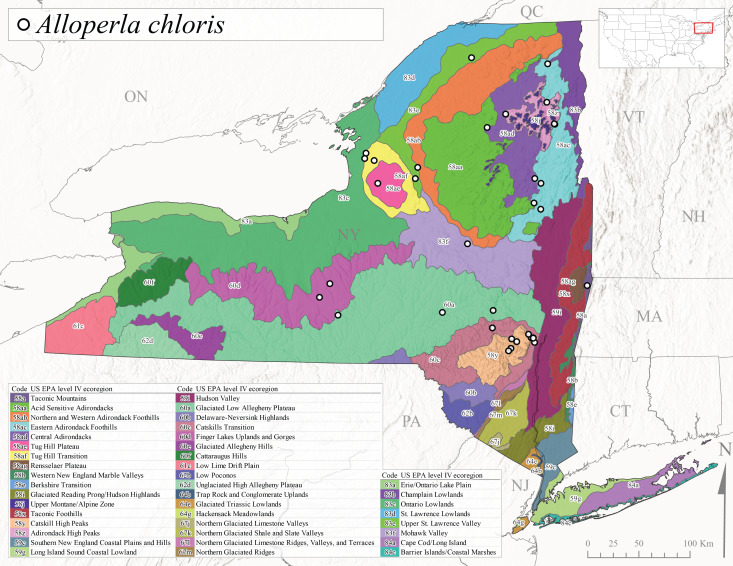
Alloperlachloris

**Figure 28a.**
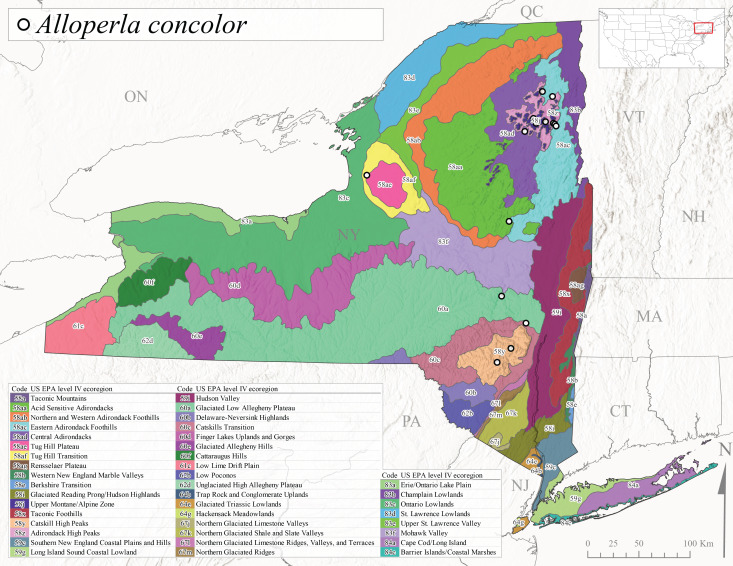
Alloperlaconcolor

**Figure 28b.**
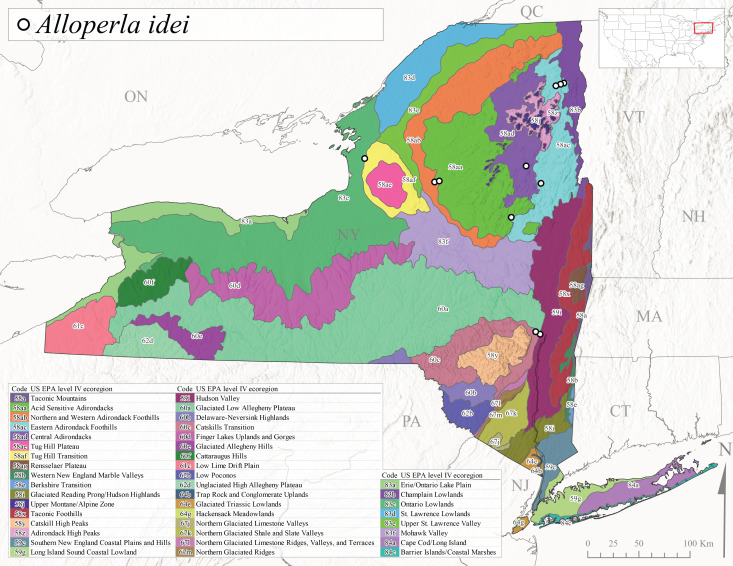
Alloperlaidei

**Figure 28c.**
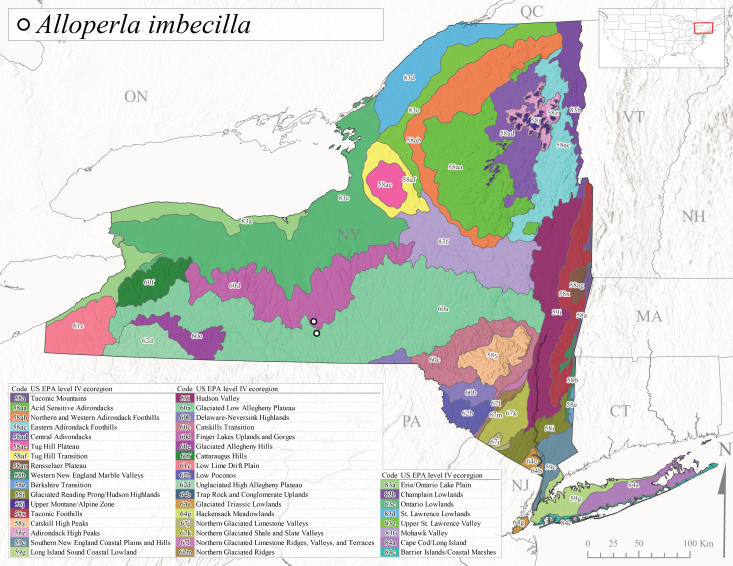
Alloperlaimbecilla

**Figure 28d.**
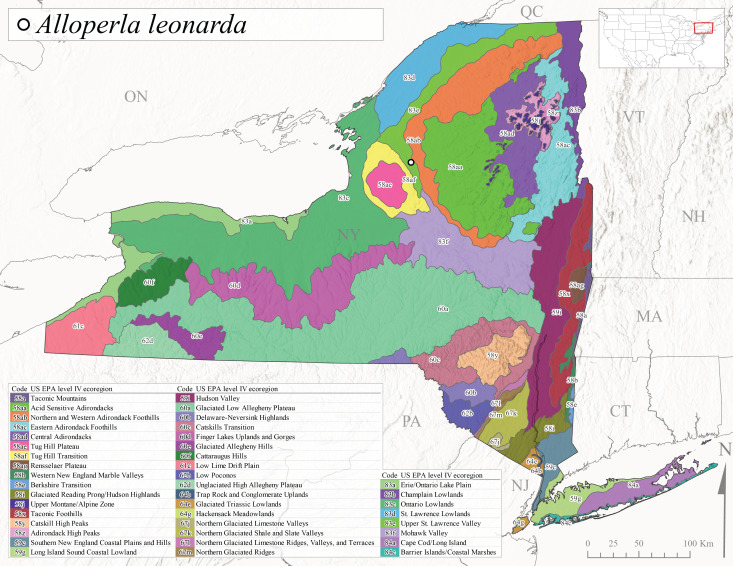
Alloperlaleonarda

**Figure 28e.**
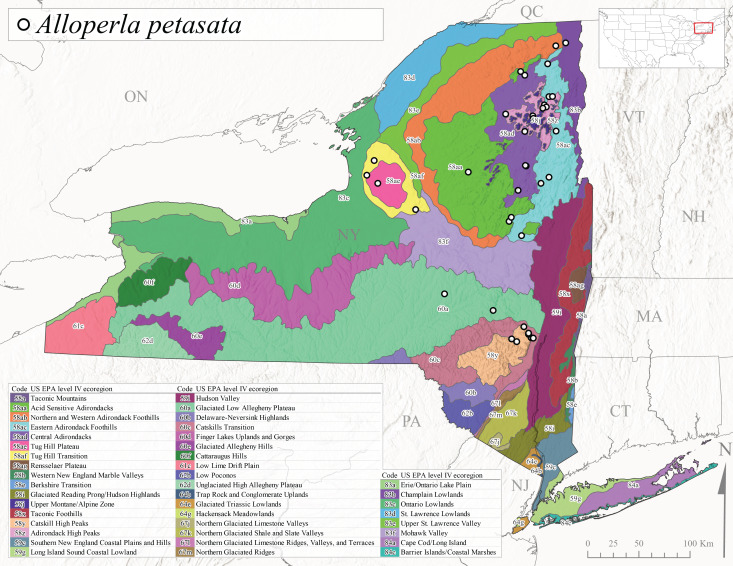
Alloperlapetasata

**Figure 28f.**
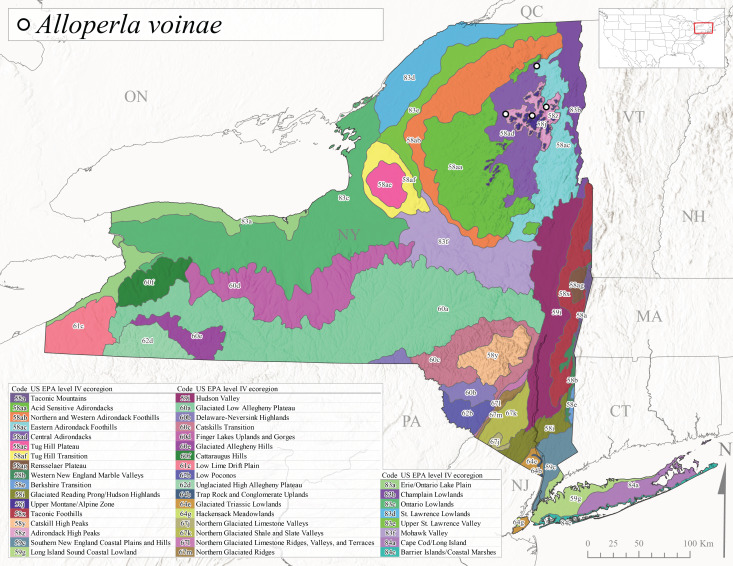
Alloperlavoinae

**Figure 29a.**
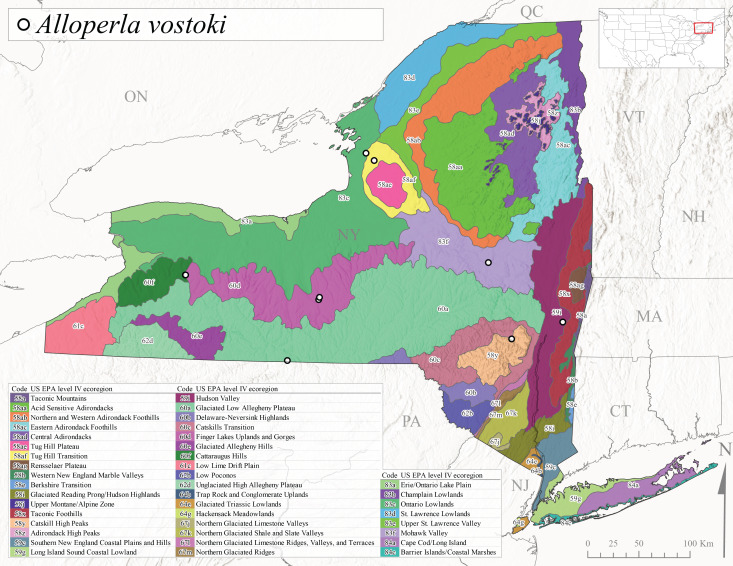
Alloperlavostoki

**Figure 29b.**
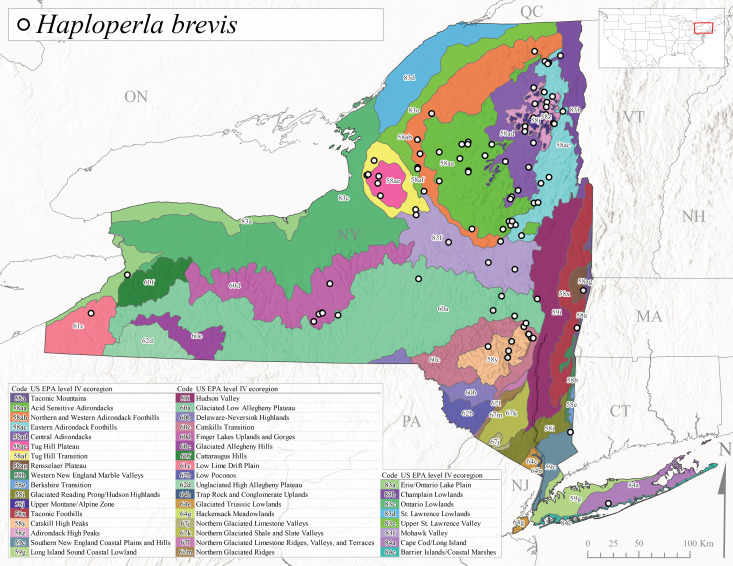
Haploperlabrevis

**Figure 29c.**
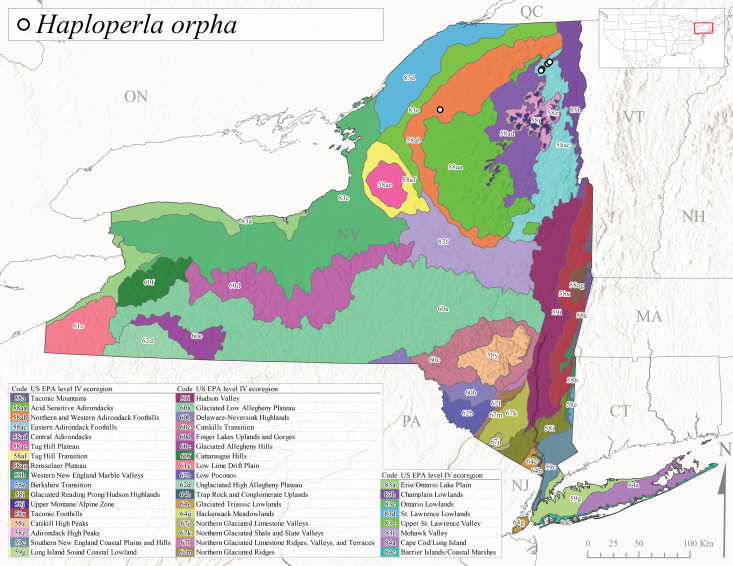
Haploperlaorpha

**Figure 29d.**
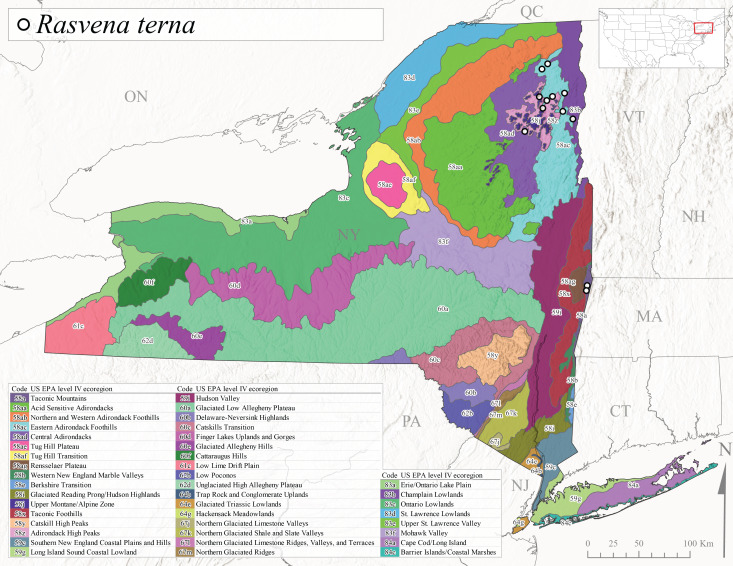
Rasvenaterna

**Figure 29e.**
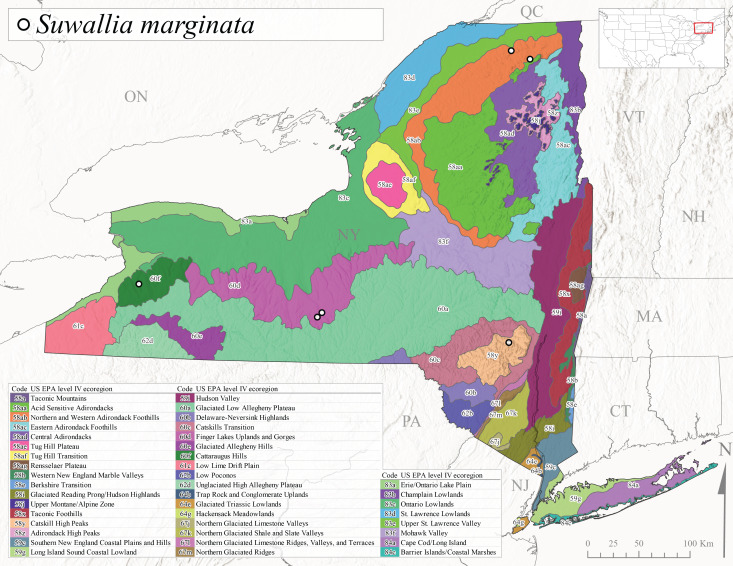
Suwalliamarginata

**Figure 29f.**
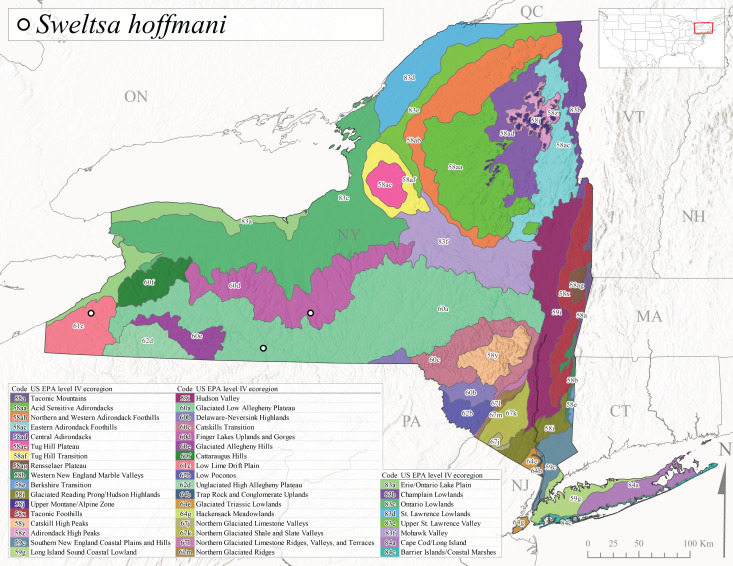
Sweltsahoffmani

**Figure 30a.**
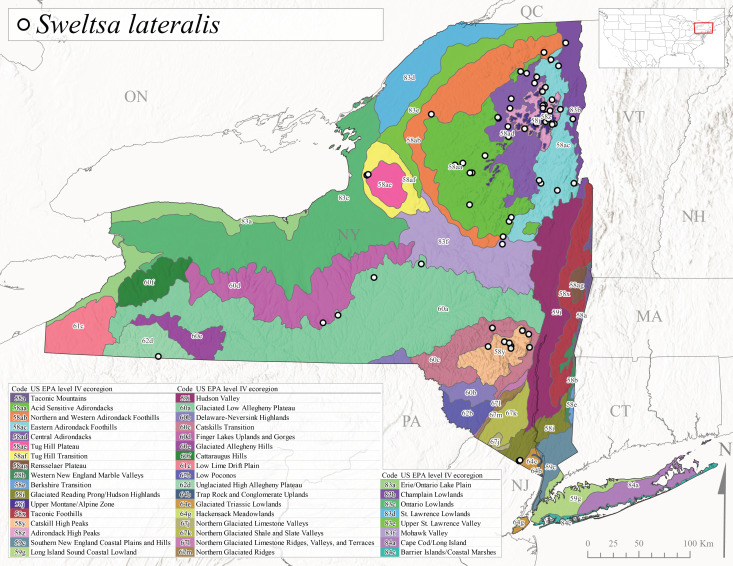
Sweltsalateralis

**Figure 30b.**
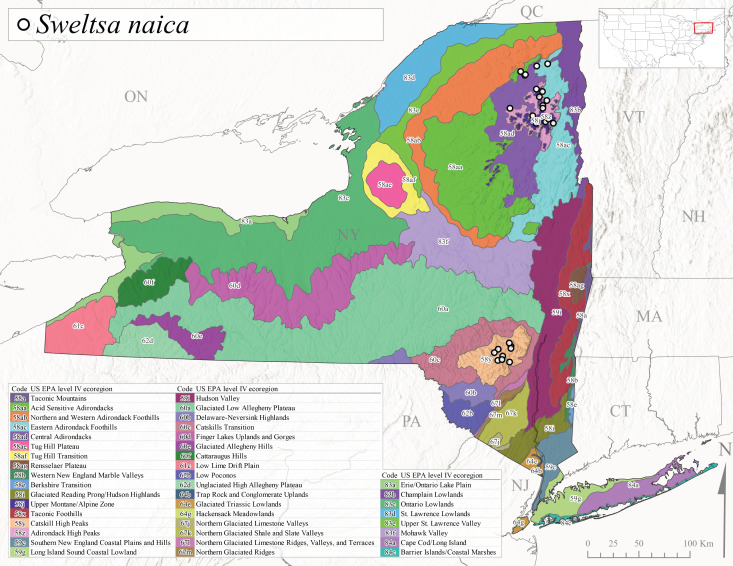
Sweltsanaica

**Figure 30c.**
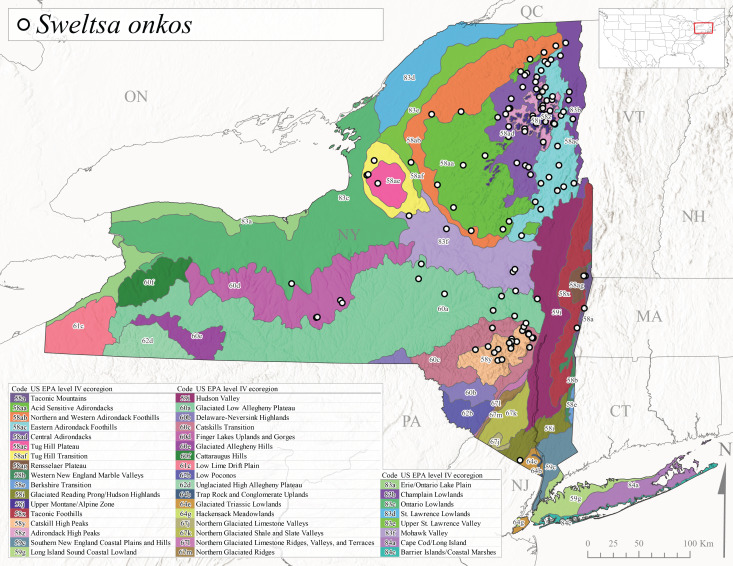
Sweltsaonkos

**Figure 30d.**
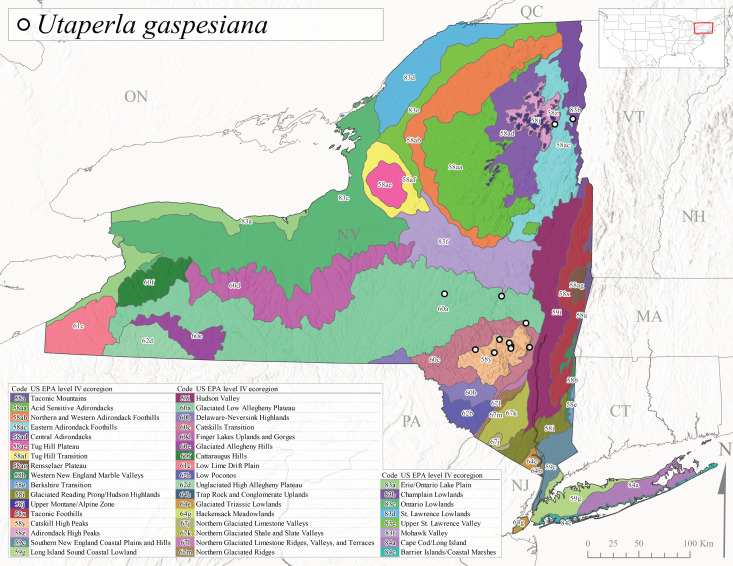
Utaperlagaspesiana

**Figure 30e.**
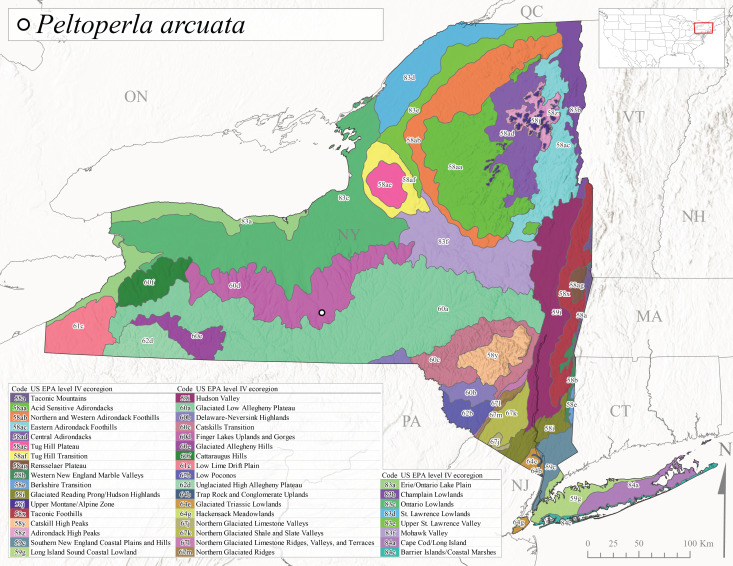
Peltoperlaarcuata

**Figure 30f.**
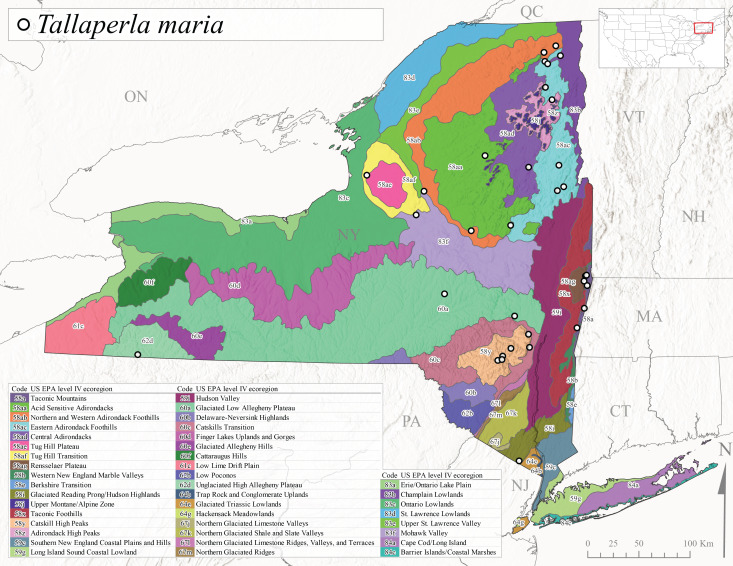
Tallaperlamaria

**Figure 35a.**
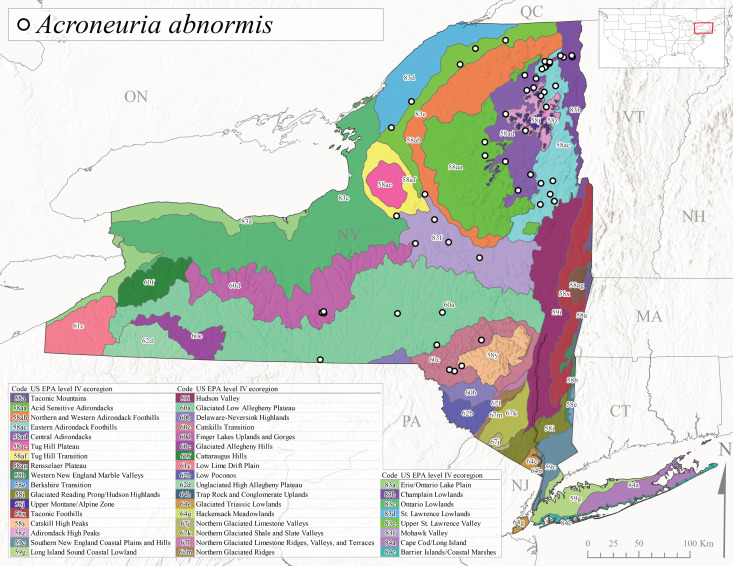
Acroneuriaabnormis

**Figure 35b.**
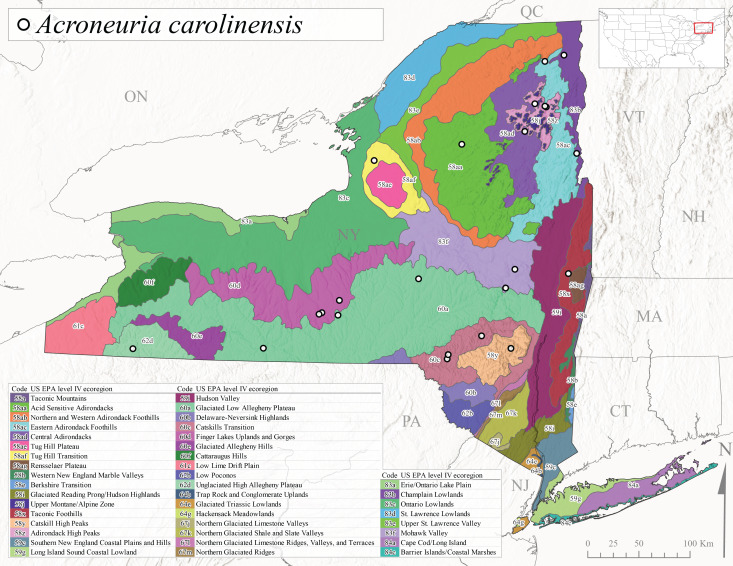
Acroneuriacarolinensis

**Figure 35c.**
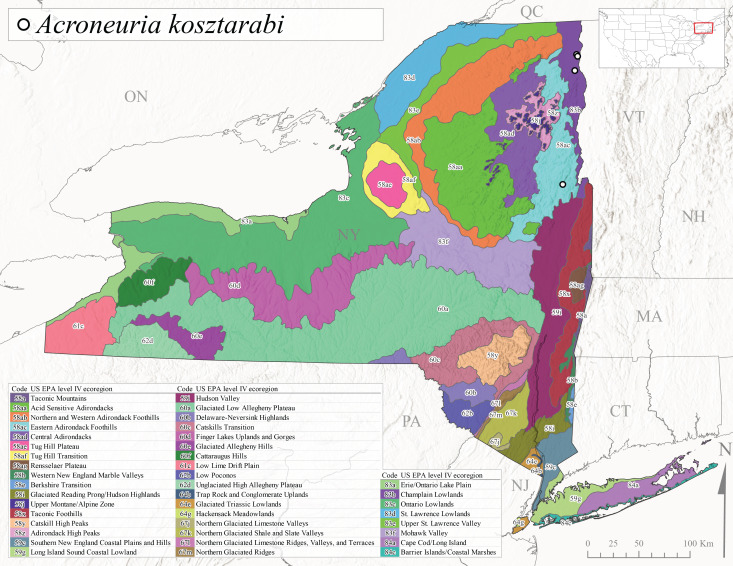
Acroneuriakosztarabi

**Figure 35d.**
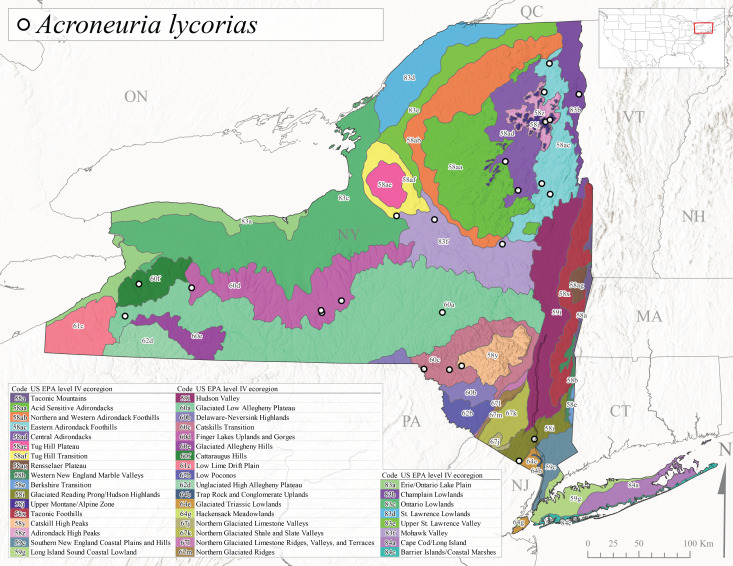
Acroneurialycorias

**Figure 35e.**
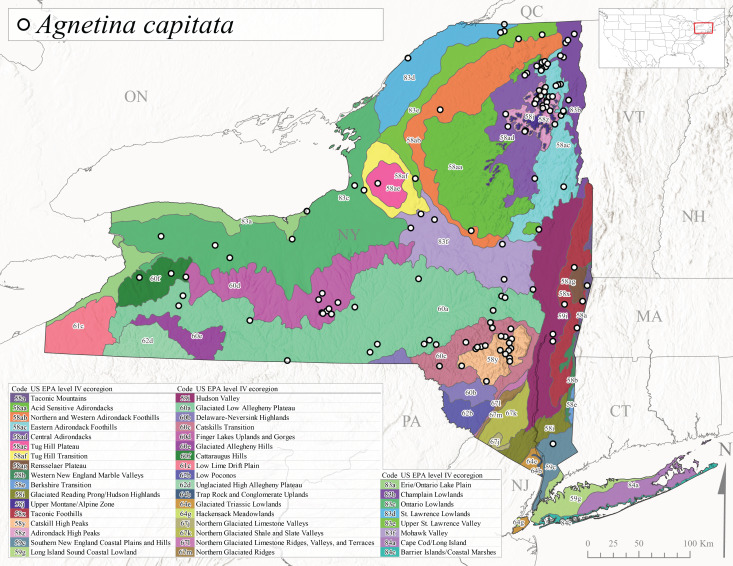
Agnetinacapitata

**Figure 35f.**
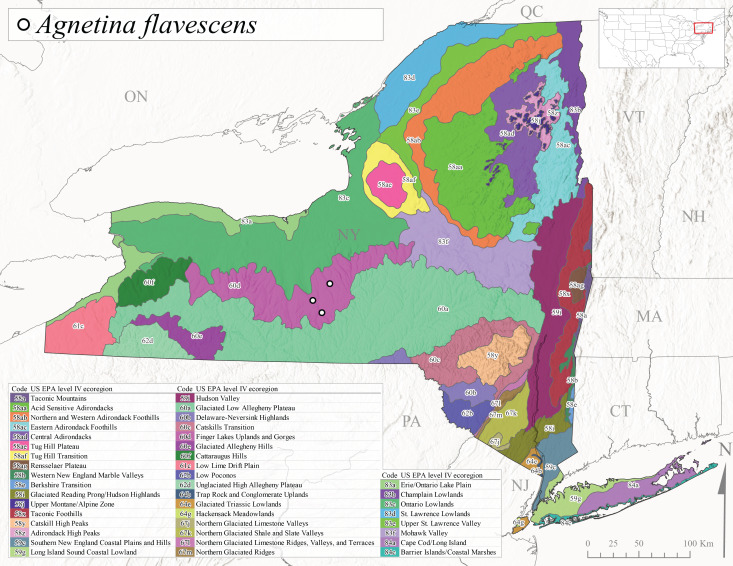
Agnetinaflavescens

**Figure 36a.**
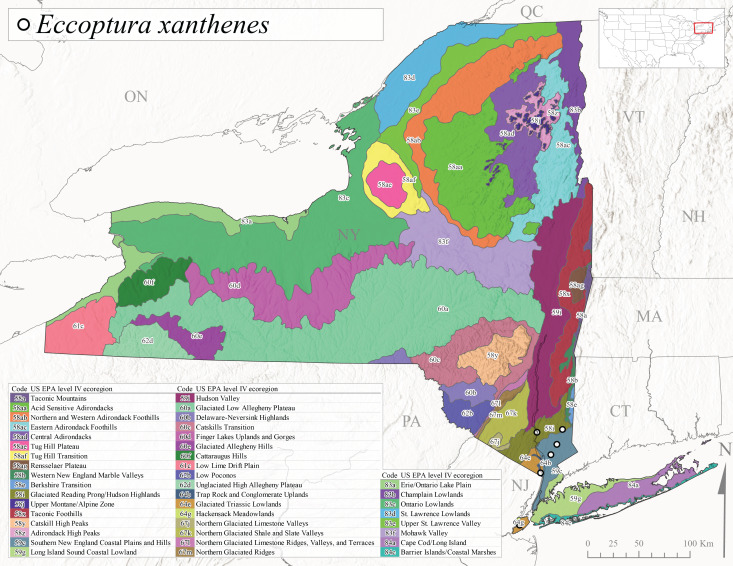
Eccopturaxanthenes

**Figure 36b.**
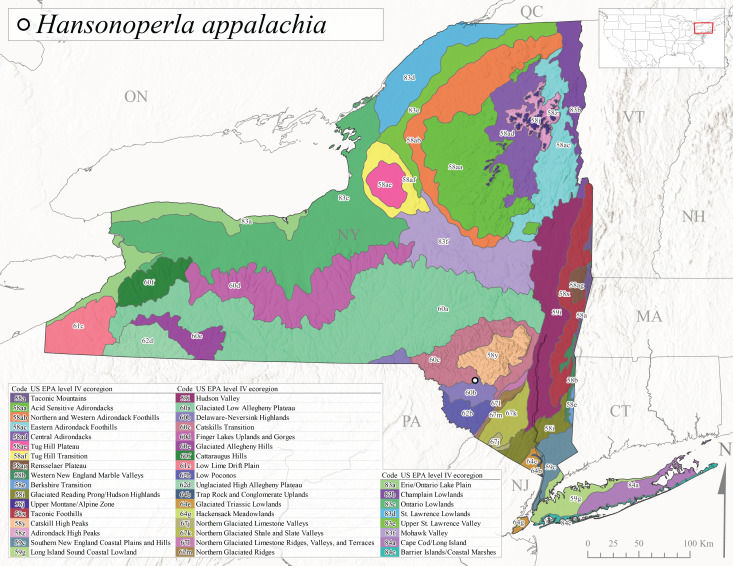
Hansonoperlaappalachia

**Figure 36c.**
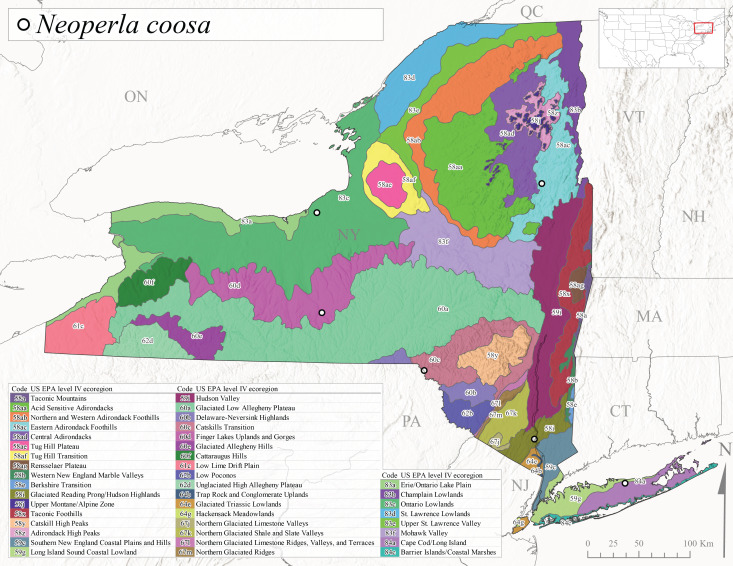
Neoperlacoosa

**Figure 36d.**
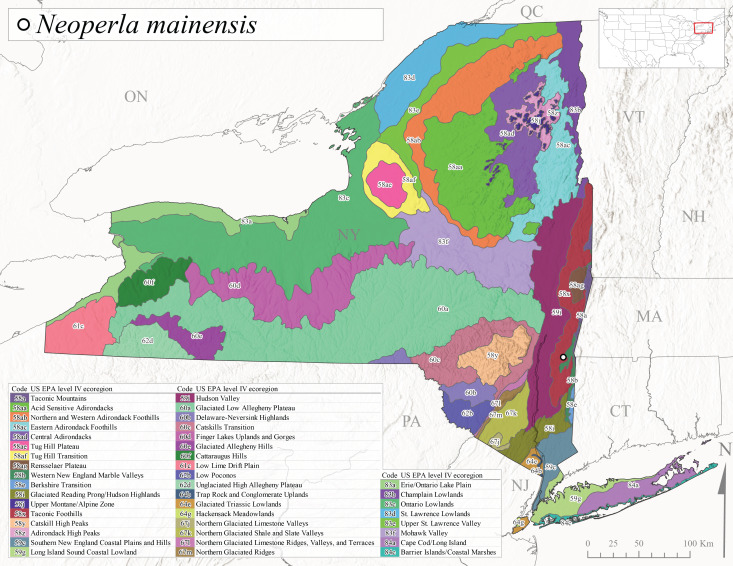
Neoperlamainensis

**Figure 36e.**
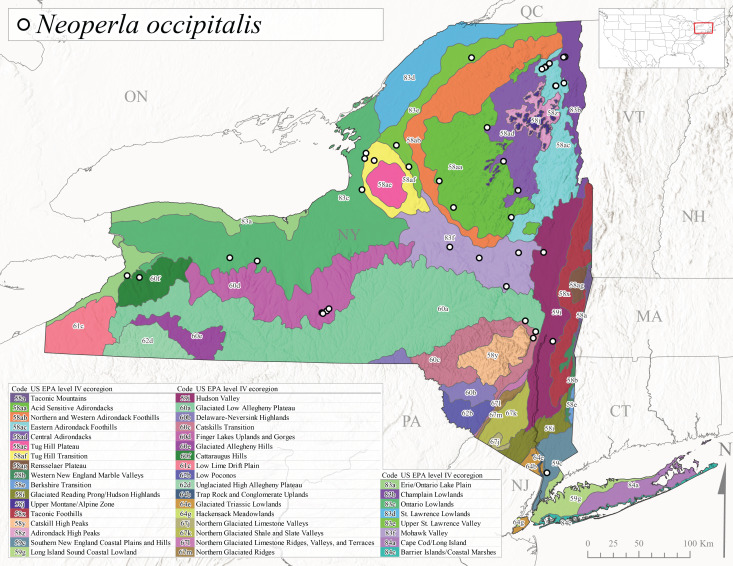
Neoperlaoccipitalis

**Figure 36f.**
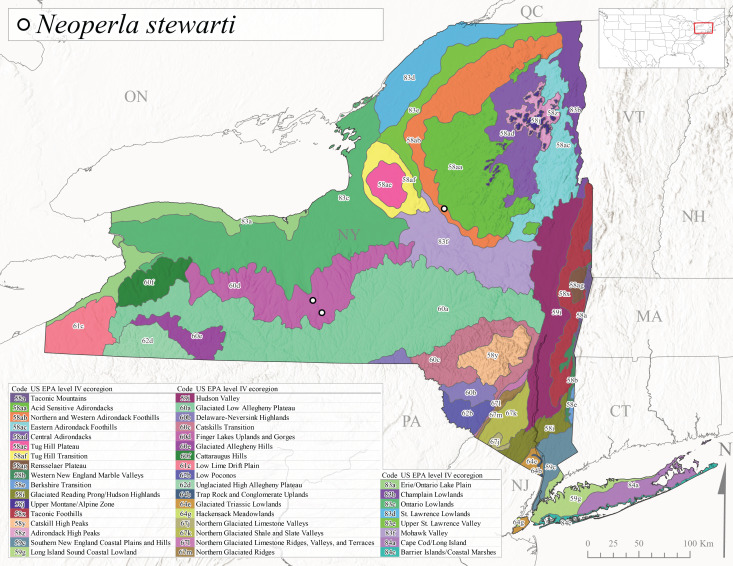
Neoperlastewarti

**Figure 37a.**
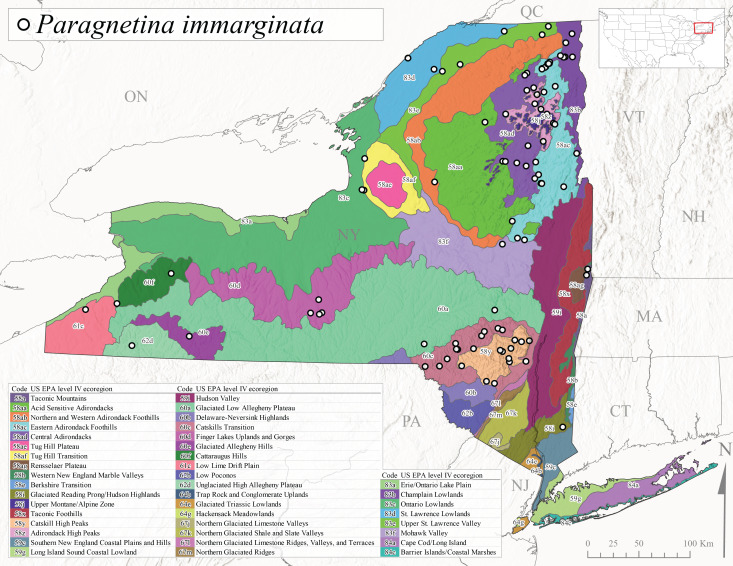
Paragnetinaimmarginata

**Figure 37b.**
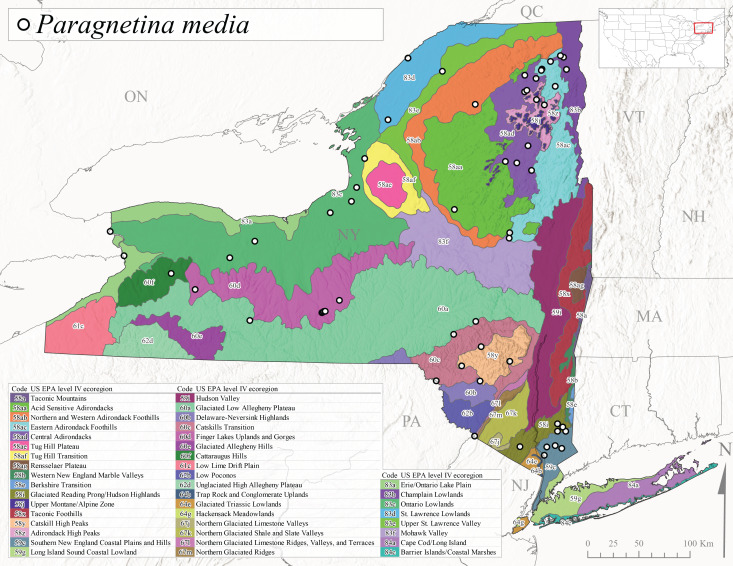
Paragnetinamedia

**Figure 37c.**
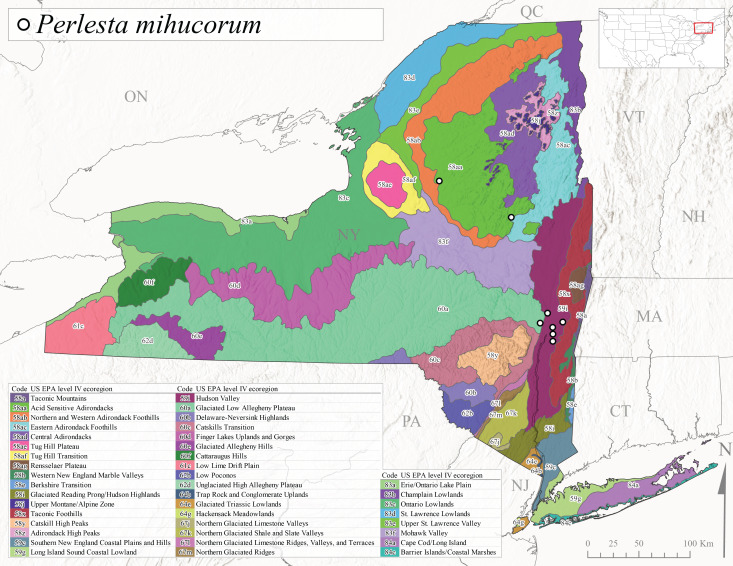
Perlestamihucorum

**Figure 37d.**
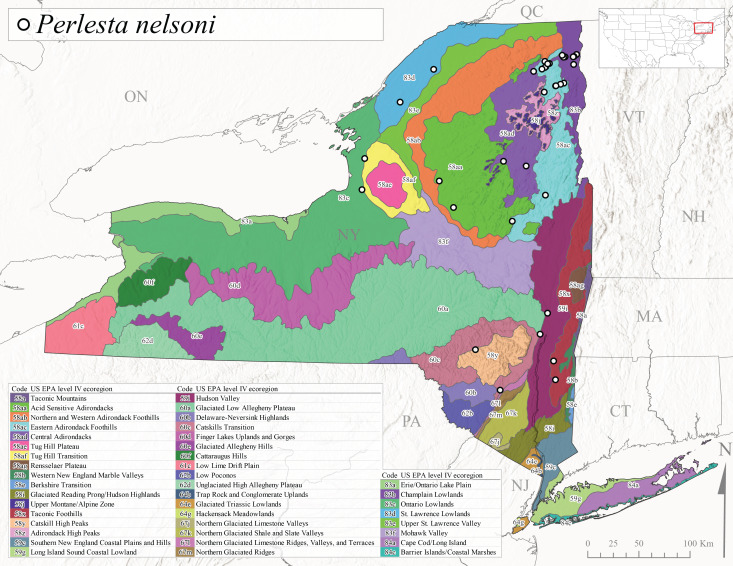
*Perlestanelsoni* Stark, 1989

**Figure 37e.**
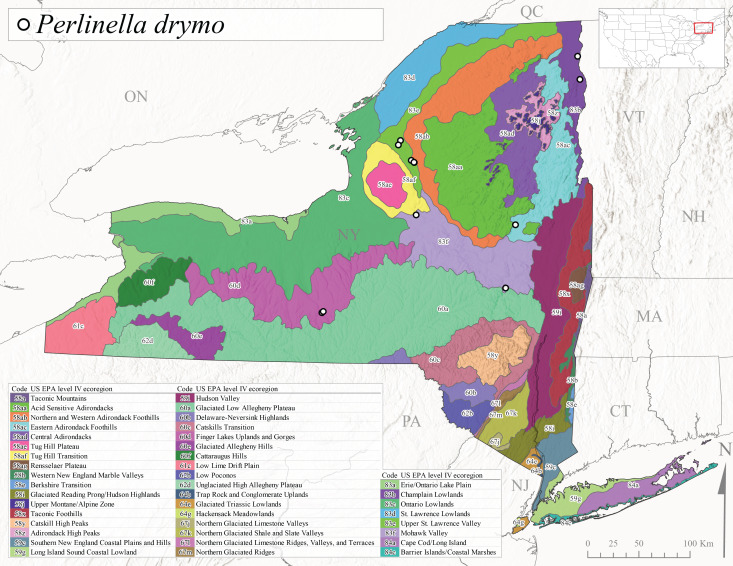
Perlinelladrymo

**Figure 37f.**
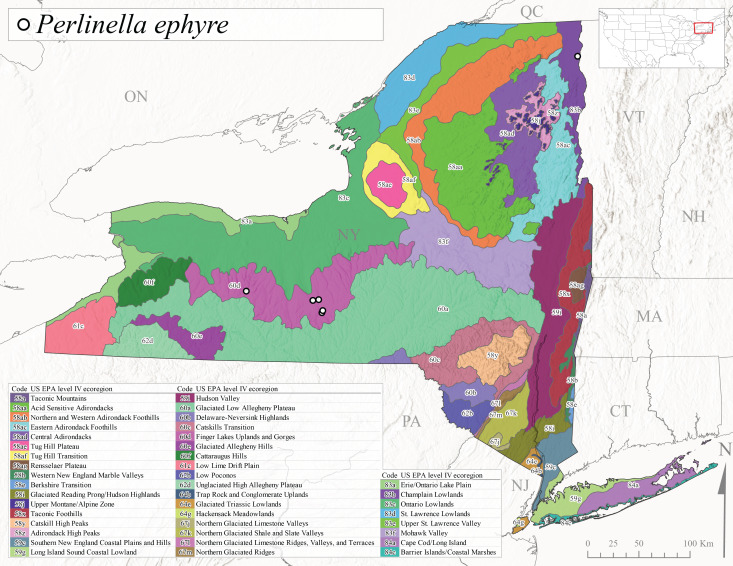
Perlinellaephyre

**Figure 40a.**
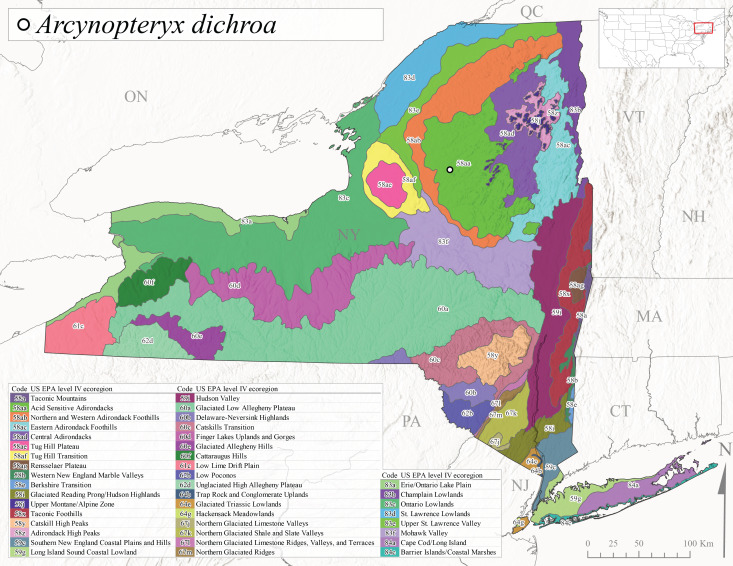
Arcynopteryxdichroa

**Figure 40b.**
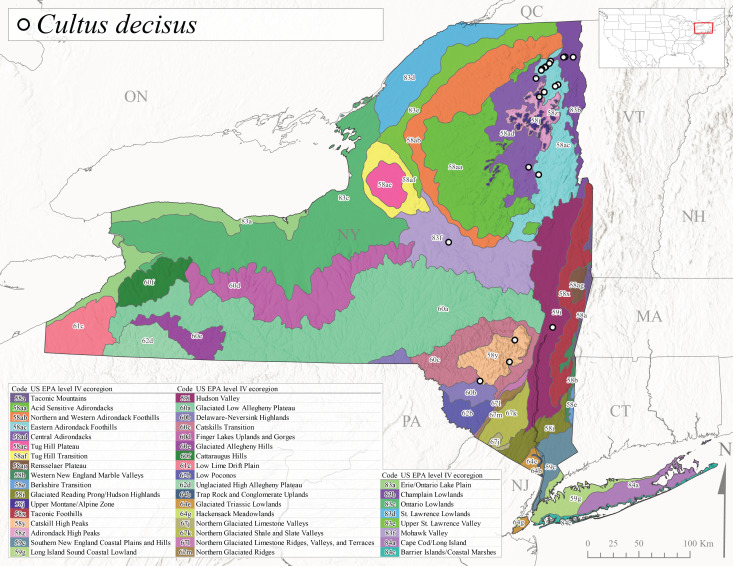
Cultusdecisusdecisus

**Figure 40c.**
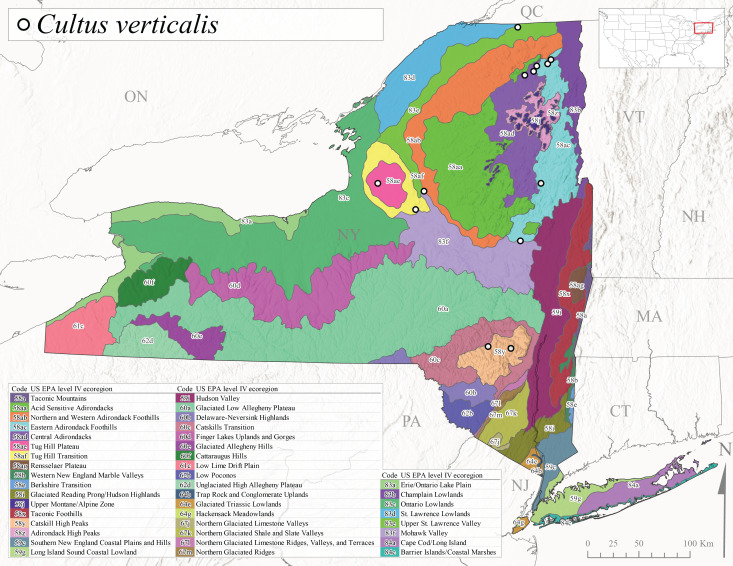
Cultusverticalis

**Figure 40d.**
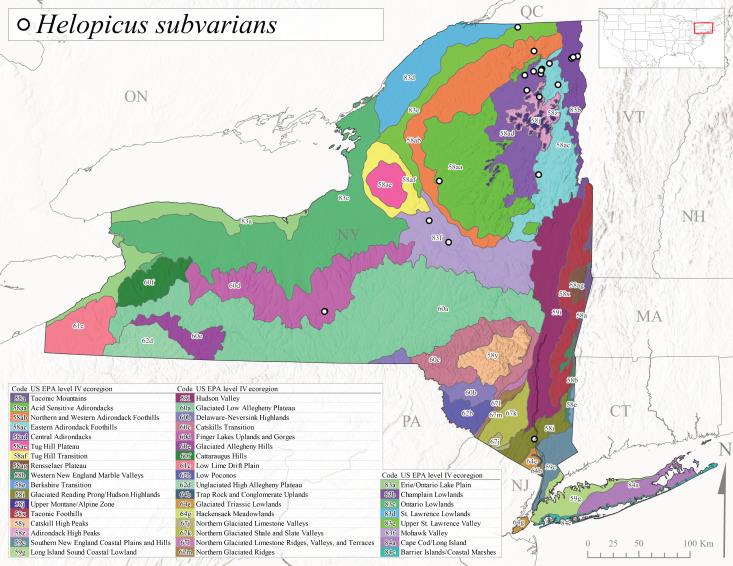
Helopicussubvarians

**Figure 40e.**
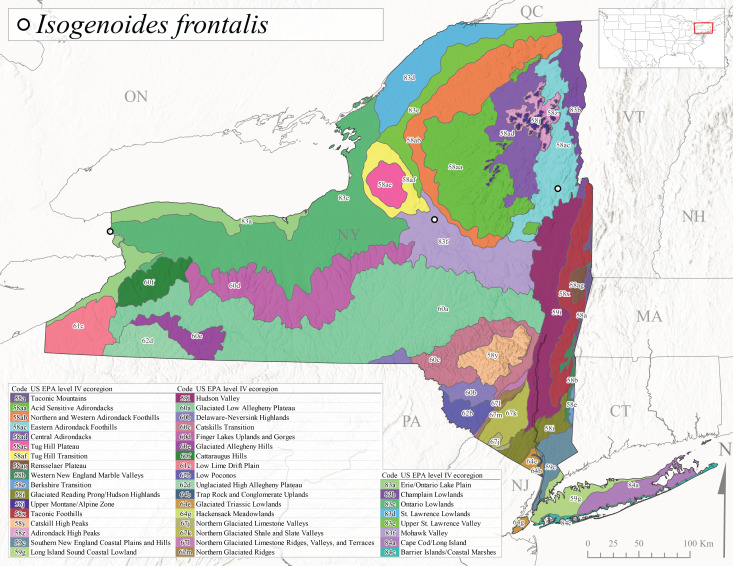
Isogenoidesfrontalis

**Figure 40f.**
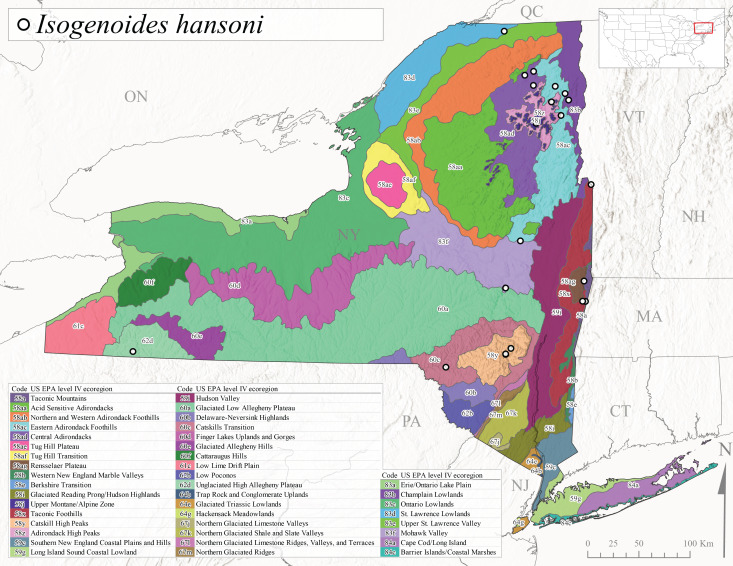
Isogenoideshansoni

**Figure 41a.**
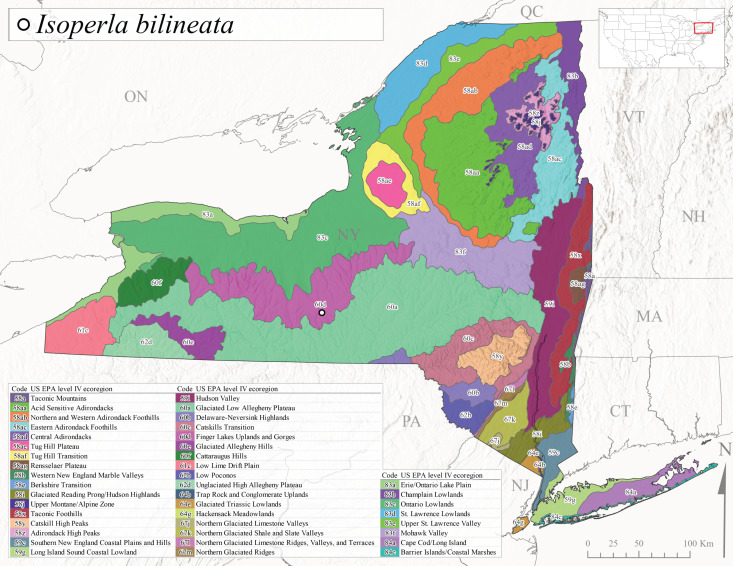
Isoperlabilineata

**Figure 41b.**
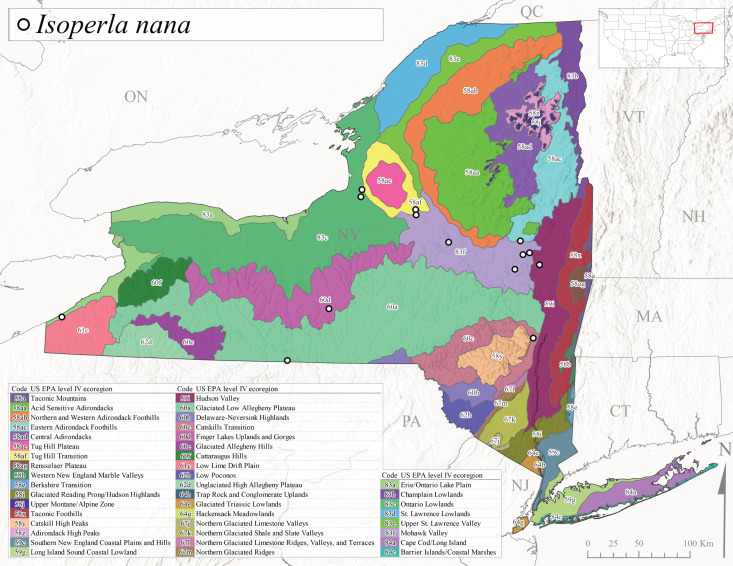
Isoperlacotta

**Figure 41c.**
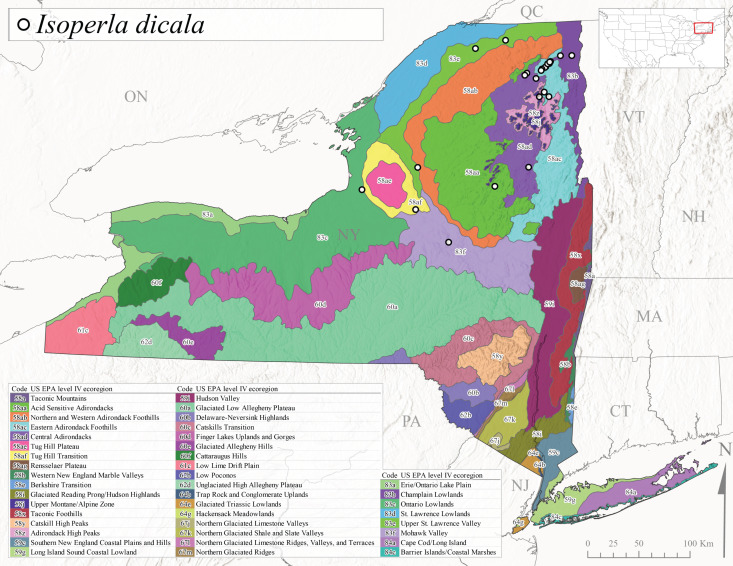
Isoperladicala

**Figure 41d.**
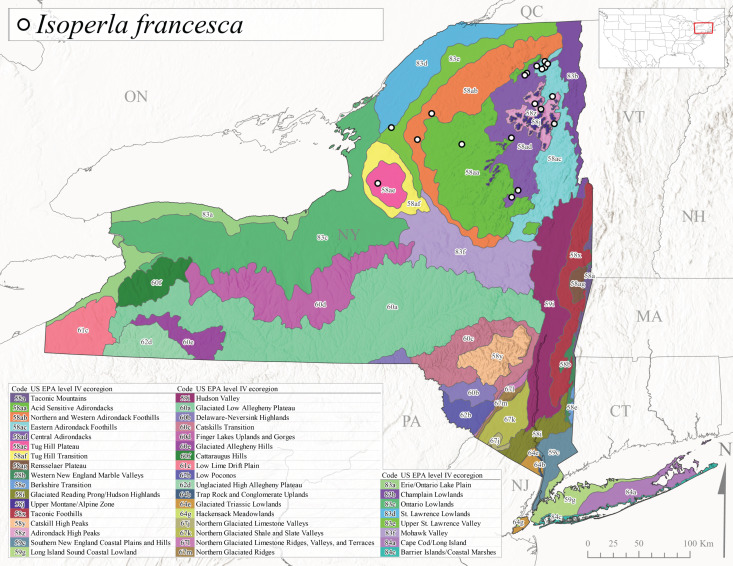
Isoperlafrancesca

**Figure 41e.**
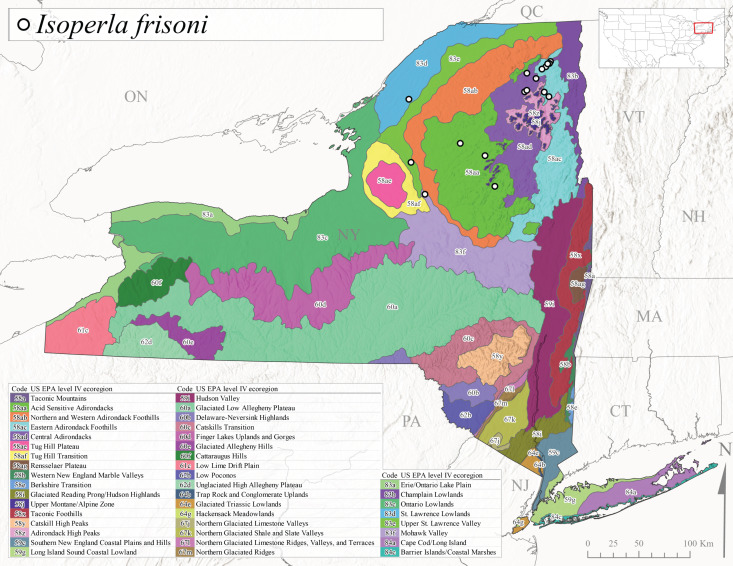
Isoperlafrisoni

**Figure 41f.**
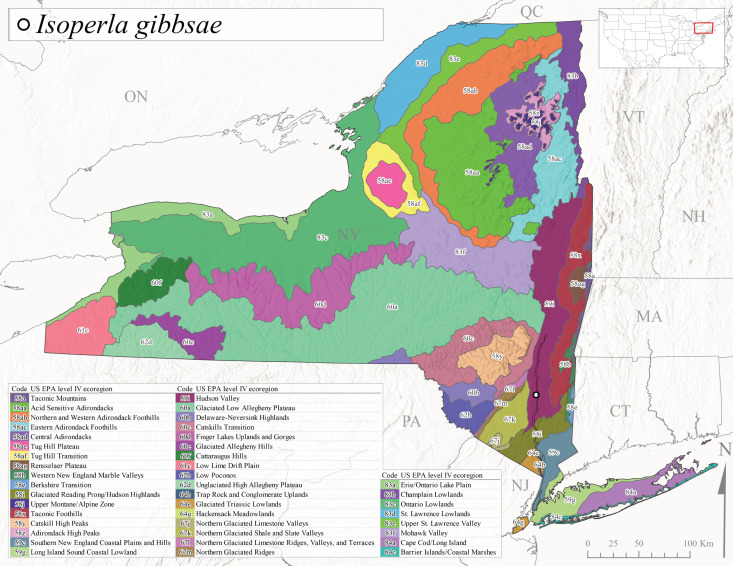
Isoperlagibbsae

**Figure 42a.**
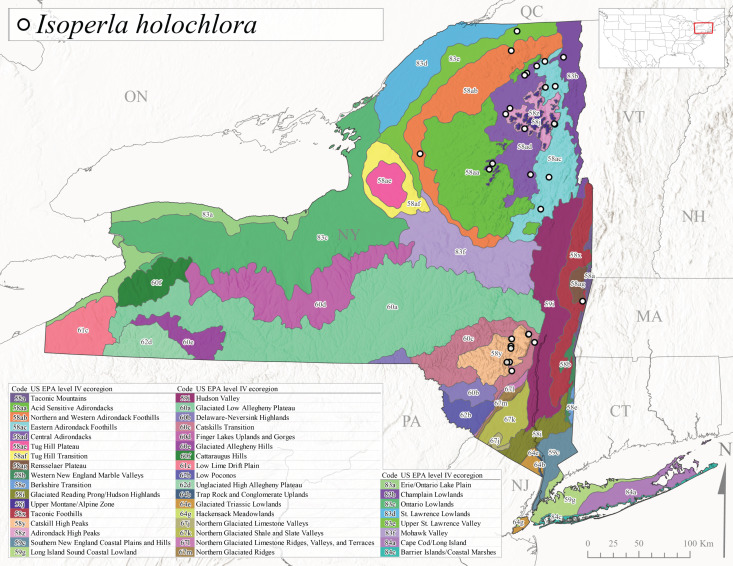
Isoperlaholochlora

**Figure 42b.**
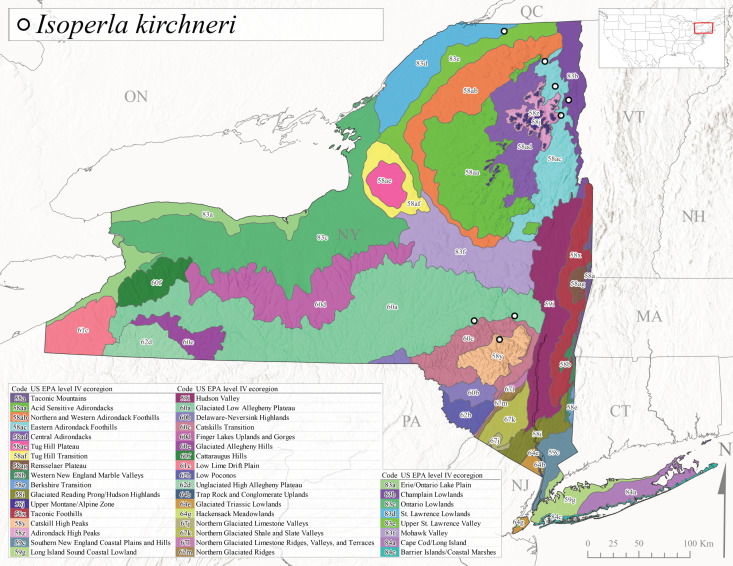
Isoperlakirchneri

**Figure 42c.**
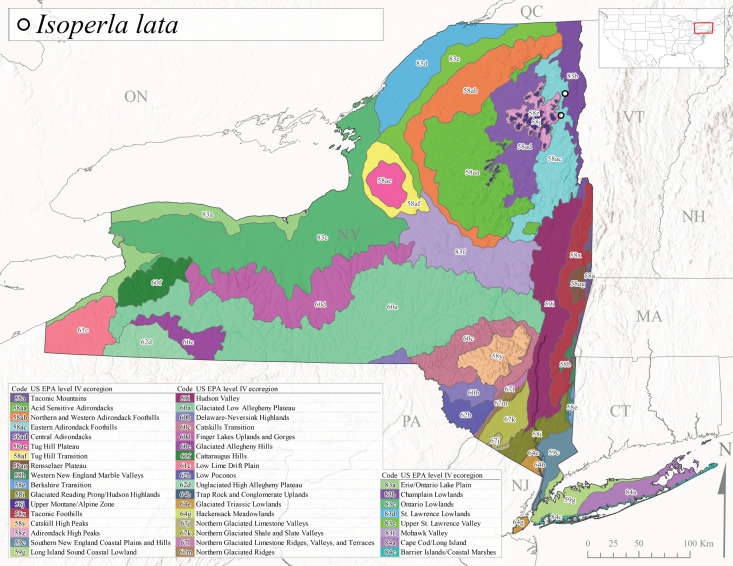
Isoperlalata

**Figure 42d.**
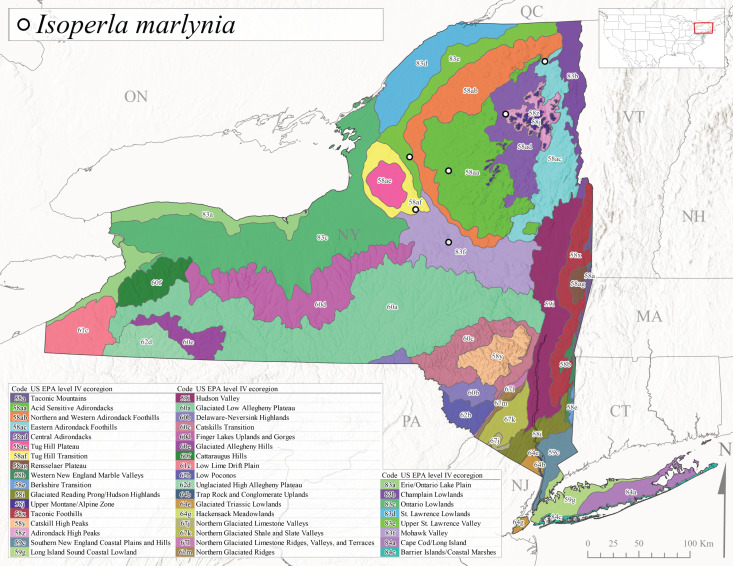
Isoperlamarlynia

**Figure 42e.**
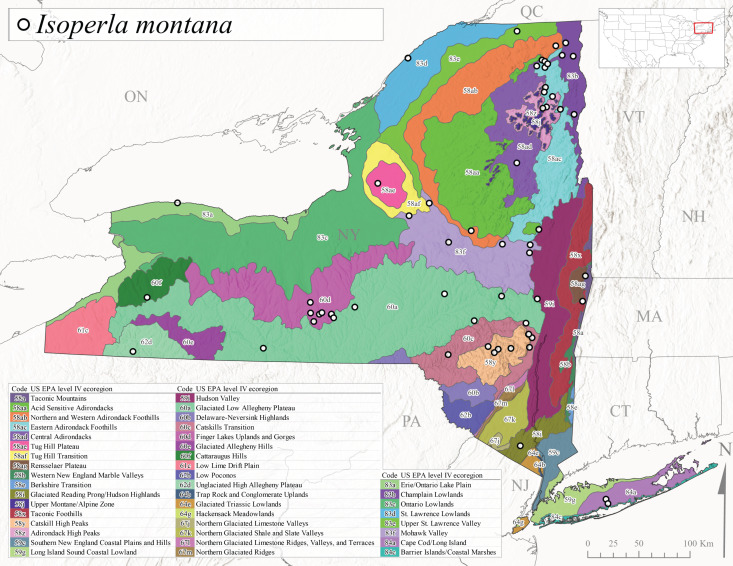
Isoperlamontana

**Figure 42f.**
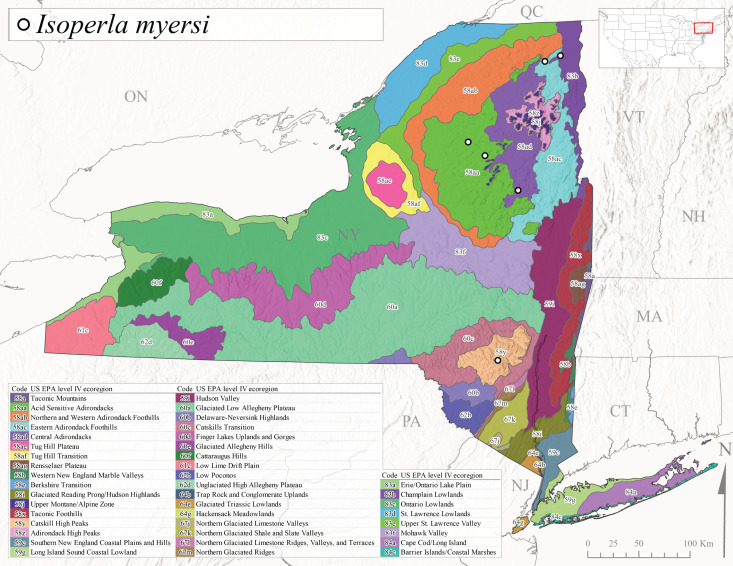
Isoperlamyersi

**Figure 43a.**
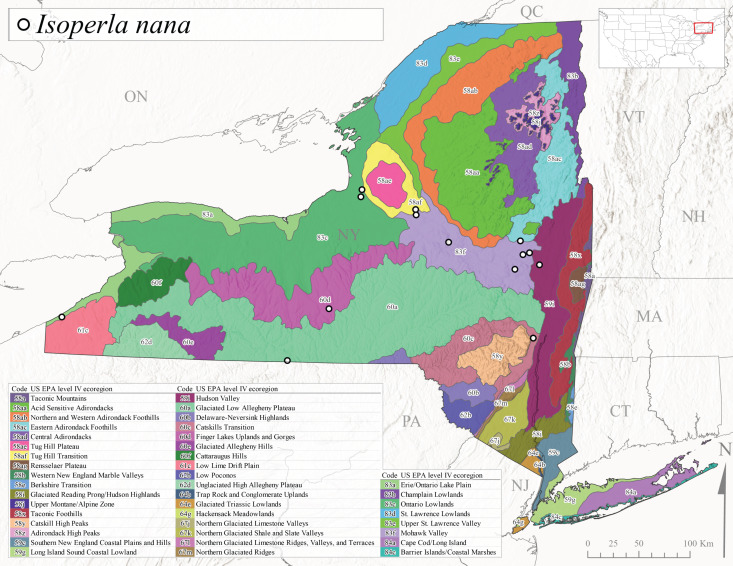
Isoperlanana

**Figure 43b.**
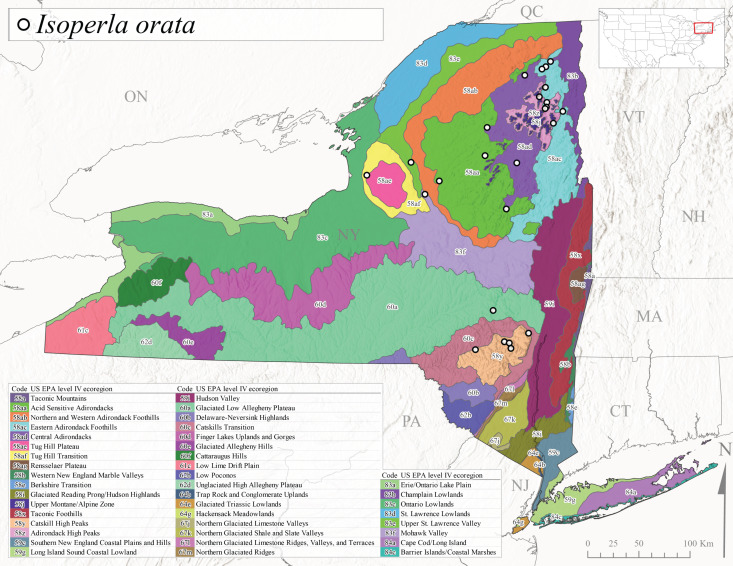
Isoperlaorata

**Figure 43c.**
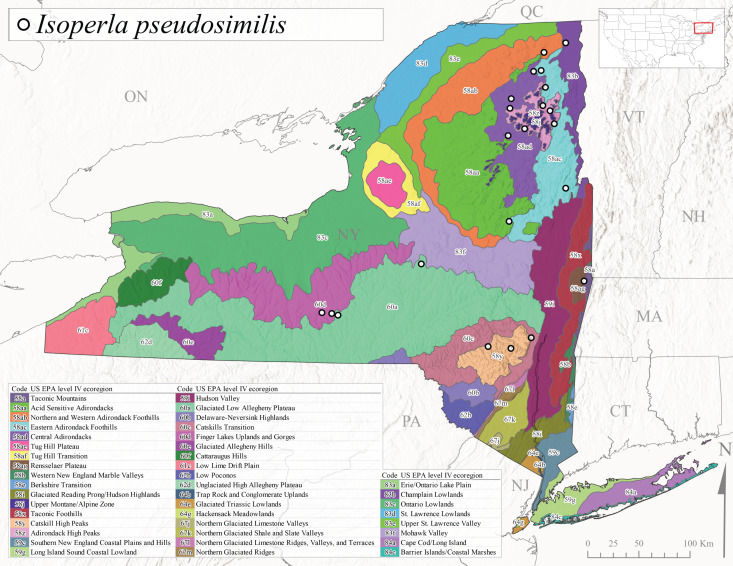
Isoperlapseudosimilis

**Figure 43d.**
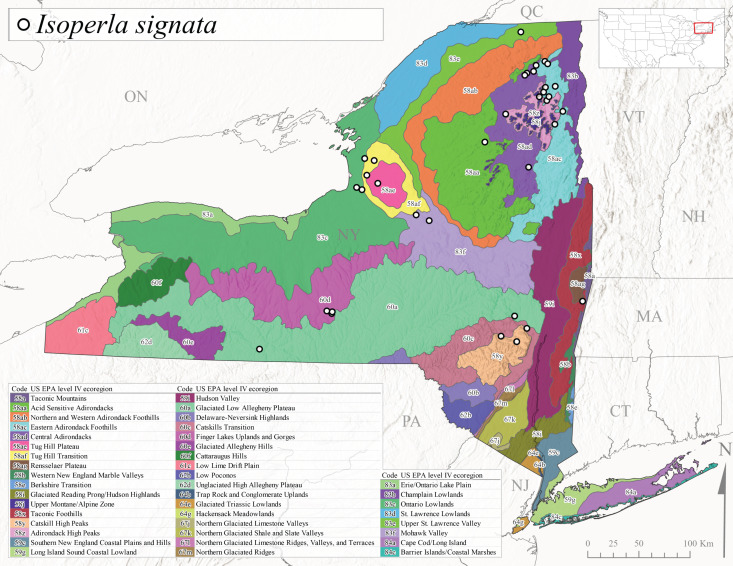
Isoperlasignata

**Figure 43e.**
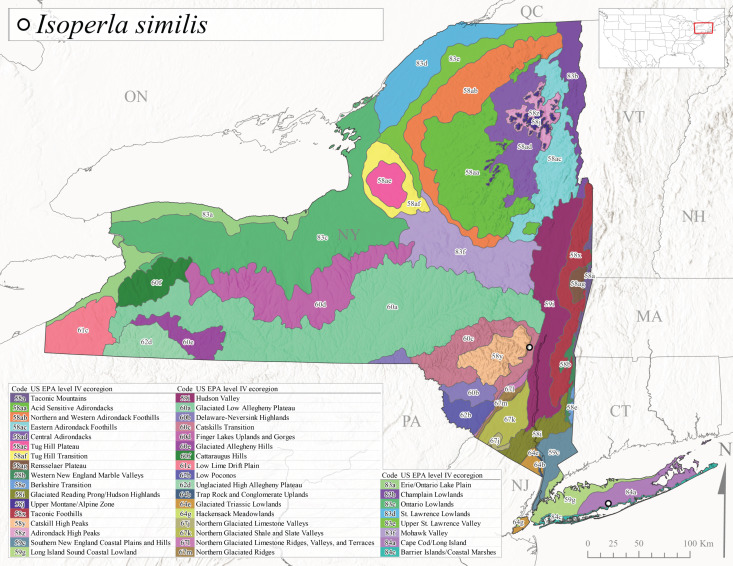
Isoperlasimilis

**Figure 43f.**
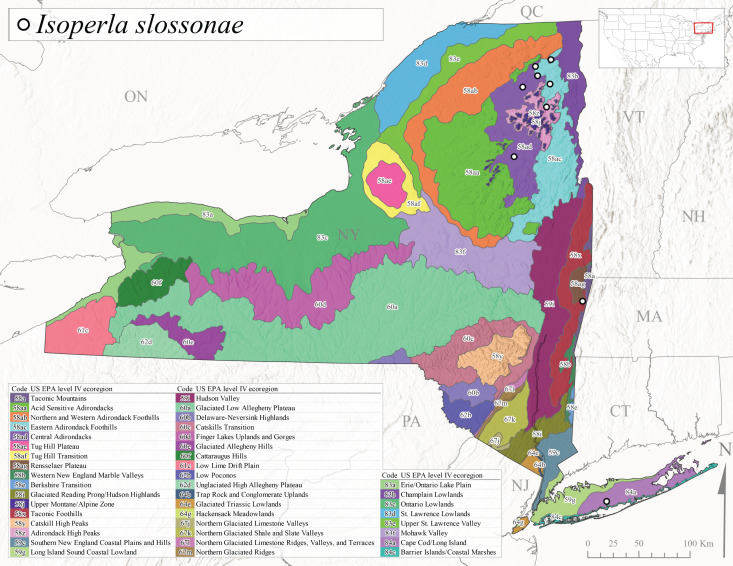
Isoperlaslossonae

**Figure 44a.**
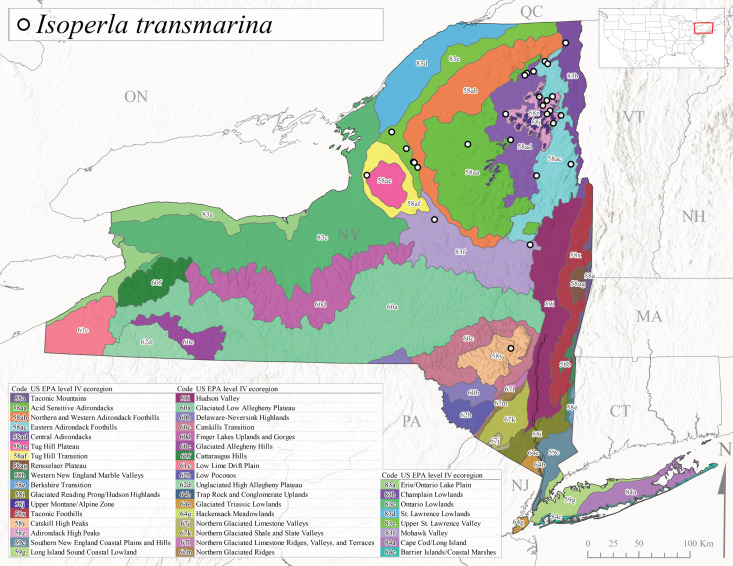
Isoperlatransmarina

**Figure 44b.**
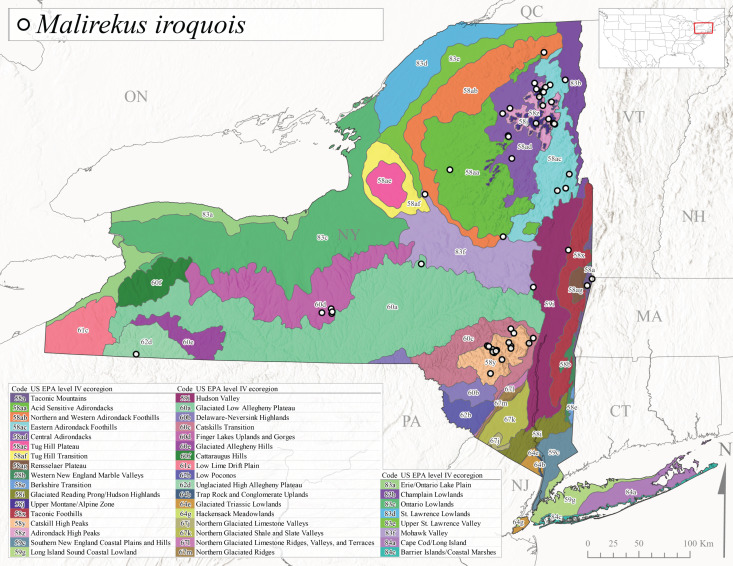
Malirekusiroquois

**Figure 44c.**
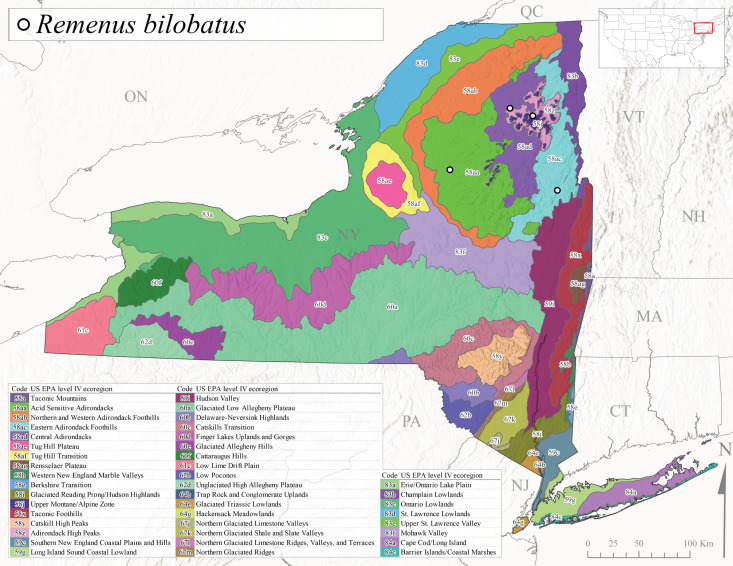
Remenusbilobatus

**Figure 44d.**
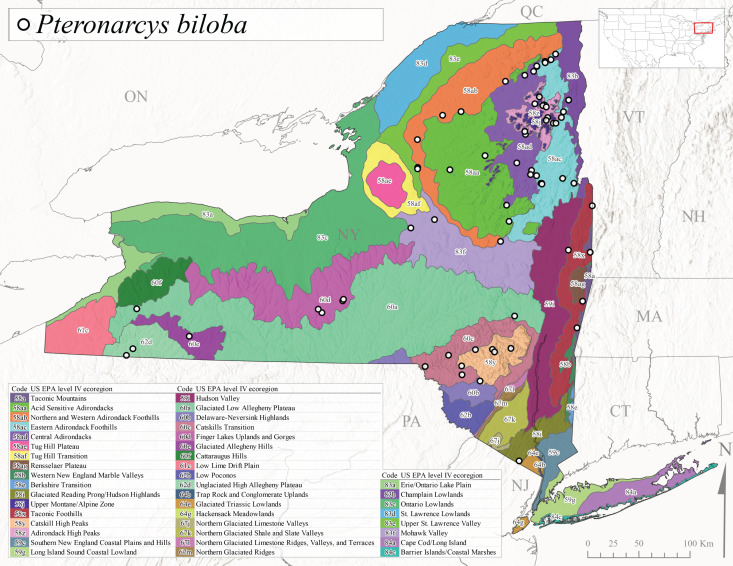
Pteronarcysbiloba

**Figure 44e.**
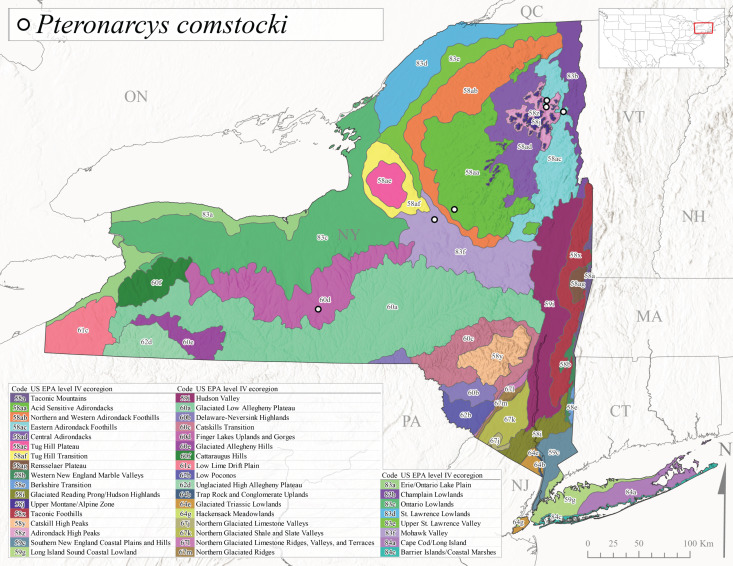
Pteronarcyscomstocki

**Figure 44f.**
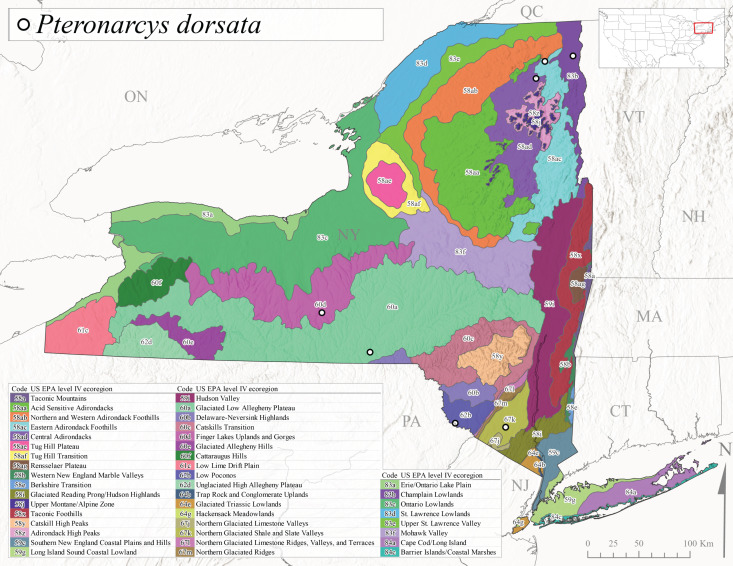
Pteronarcysdorsata

**Table 1.** List of Plecoptera with holotypes designated from New York State (*syntypes or other designated primary type, **lectotype). Current valid name provided.

Current valid name	County	Locality
*Allocapniagranulata*	Fulton	Johnstown
**Allocapnianivicola	unspecified	New York State
*Allocapniapechumani*	Herkimer	Otsquago Creek, Starkville
*Allocapniarecta*	Tompkins	Ithaca
*Leuctraduplicata*	Cortland/Onondaga	Labrador Lake
*Leuctrasibleyi*	Tompkins	Moore's Brook, Ithaca
*Leuctratriloba*	Tompkins	McLean
*Leuctratruncata*	Herkimer	Old Forge
*Paraleuctrasara*	Tompkins	Ringwood Lloyd Preserve, near Ithaca
*Amphinemurawui*	Tompkins	Ithaca
*Ostrocercacomplexa*	Essex	Artist's Brook
*Prostoiasimilis*	Oneida	Clinton
*Soyedinavallicularia*	Tompkins	Ithaca
*Taeniopteryxnivalis*	unspecified	New York State, Missing Holotype
*Alloperlabanksi*	Montgomery	Flat Creek, Flat Creek Town
*Alloperlachloris*	Tompkins	Caroline Lloyd-Cornell Wildflower Preserve
*Suwalliamarginata	Erie	Colden
*Peltoperlaarcuata*	Tompkins	Ithaca
*Acroneuriaabnormis*	Herkimer/Oneida	Trenton Falls
*Acroneurialycorias*	Herkimer/Oneida	Trenton Falls
*Perlestamihucorum*	Columbia	Claverack Creek, Rt. 66 near Hudson
*Isogenoidesfrontalis*	Herkimer/Oneida	Trenton Falls
*Isoperlamyersi*	Ulster	Big Indian Hollow, Oliveria Rd.
*Isoperlapseudosimilis*	Franklin	Dutton Brook, Route 3 near Saranac Lake
*Isoperlatransmarina	Herkimer/Oneida	Trenton Falls
*Pteronarcysbiloba	Herkimer/Oneida	Trenton Falls
*Pteronarcyscomstocki*	Hamilton/Herkimer	Wilmurt
*Pteronarcysproteus	Herkimer/Oneida	Trenton Falls

**Table 2.** Institutional collections and organizations housing specimens examined for this study.

Museum Codon	Collection Name
BYUC	Brigham Young University Collection, Provo, Utah
CHNC	Charlie H. Nelson Personal Collection, Chattanooga, Tennessee
CLEV	Cleveland Museum of Natural History
CSUIC	C. P. Gillette Museum of Arthropod Diversity Colorado State University, Fort Collins
CUIC	Cornell University Insect Collections, Cornell University, Ithaca, New York
DEBU	University of Guelph, Ontario, Canada
FMNH	Chicago Field Museum of Natural History, Chicago, Illinois
INHS	Illinois Natural History Survey, Prairie Research Institue, University of Illinois at Urbana-Champaign
ISIC	Iowa State Insect Insect Collection, Ames, Iowa
LCRI	Lake Champlain Research Institute, SUNY Plattsburgh, Plattsburgh, New York
MRPC	Martin Rosenfeld Personal Collection
MSUC	Michigan State University, East Lansing
NDUC	University of Notre Dame, Museum of Biodiversity Arthropod Collection
NYCDEP	New York City Department of Environmental Protection
NYSM	New York State Museum, Albany, New York
OEPA	Ohio Environmental Protection Agency
PERC	Purdue University Entomology Research Collection, West Lafayette, Indiana
PNHC	Phillip N. Hogan Collection, University of Illinois, Urbana, Illinois
UMMZ	University of Michigan Museum of Zoology
UMSP	University of Minnesota St. Paul
UVM	University of Vermont Insect Collection, Burlington, Vermont
UWIRC	University of Wisconsin Insect Research Collection, Madison
WKUC	Western Kentucky University Collection, Bowling Green

**Table 3.** Chronological bibliography of Plecoptera records from New York State.

Newman (1838)	Johnson (1981)
Newman (1839)	Stark and Szczytko (1981)
Fitch (1847)	Stark (1983)
Hagen (1861)	Surdick (1985)
Banks (1897)	Stark (1986)
Needham and Betten (1901)	Bilger (1986)
Needham (1905)	Stark and Kondratieff (1987)
Banks (1911)	Kondratieff et al. (1988)
Smith (1913)	Stark et al. (1988)
Needham and Smith (1916)	Stark and Szczytko (1988)
Smith (1917)	Kondratieff and Kirchner (1993)
Claassen (1923)	Stanger and Baumann (1993)
Wu (1923)	Peckarsky (1994)
Claassen (1924)	Kondratieff and Nelson (1995)
Needham (1925)	Baumann (1996a)
Needham and Claassen (1925)	Alexander and Stewart (1999)
Needham and Claassen (1926)	Stark and Kyzar (2001)
Claassen (1931)	Stark and Baumann (2004)
Frison (1934)	Sandberg and Stewart (2005)
Claassen (1937)	Grubbs (2006)
Hanson (1938)	Baumann and Kondratieff (2009)
Ricker (1938)	Kondratieff and Kirchner (2009)
Frison (1942)	Myers and Kondratieff (2009)
Hanson (1942)	Harrison and Stark (2010)
Hanson (1943)	Stark and Kondratieff (2010)
Banks (1948)	Kondratieff and Myers (2011)
Ross and Ricker (1964)	Myers et al. (2011)
Ross and Freytag (1967)	Stark and Kondratieff (2012)
Hitchcock (1968)	Grubbs (2015)
Nelson and Hanson (1968)	Stark et al. (2016)
Ricker and Ross (1968)	Grubbs and Wei (2017)
Harper and Hynes (1971a)	Myers and Kondratieff (2017)
Nelson and Hanson (1971)	Verdone and Kondratieff (2017)
Ross and Ricker (1971)	Grubbs (2018)
Baumann (1974)	Grubbs and Singai (2018)
Ricker and Ross (1975)	Verdone and Kondratieff (2018)
Stark and Gaufin (1976a)	Grubbs and Baumann (2019)
Stark and Gaufin (1976b)	Grubbs et al. (2020)
Stark and Baumann (1978)	Grubbs and Baumann (2021)
Szczytko and Stewart (1978)	Grubbs and Baumann (2023)
Fullington and Stewart (1980)	Verdone et al. (2025)
